# Isolation and Structure Elucidation of New Metabolites from the Mariana-Trench-Associated Fungus *Aspergillus* sp. SY2601

**DOI:** 10.3390/molecules29020459

**Published:** 2024-01-17

**Authors:** Cangzhu Sun, Yura Ha, Xin Liu, Nan Wang, Xiao-Yuan Lian, Zhizhen Zhang

**Affiliations:** 1 Ocean College, Zhoushan Campus, Zhejiang University, Zhoushan 316021, China; cangzhusun@163.com (C.S.); solusean@gmail.com (Y.H.); liuxin66@zju.edu.cn (X.L.); 2 Hainan Institute of Zhejiang University, Sanya 572025, China; 3 College of Pharmaceutical Sciences, Zhejiang University, Hangzhou 310058, China; xylian@zju.edu.cn

**Keywords:** marine fungus, *Aspergillus* sp. SY2601, indolyl diketopiperazine, aspyrone analogue, putative meroterpenoid, antimicrobial activity

## Abstract

Fungi are important resource for the discovery of novel bioactive natural products. This study investigated the metabolites produced by Mariana-Trench-associated fungus *Aspergillus* sp. SY2601 in EY liquid and rice solid media, resulting in the isolation and structure determination of 28 metabolites, including five new compounds, asperindopiperazines A–C (**1**–**3**), 5-methoxy-8,9-dihydroxy-8,9-deoxyaspyrone (**21**), and 12*S*-aspertetranone D (**26**). Structures of the new compounds were elucidated based on extensive NMR spectral analyses, HRESIMS data, optical rotation, ECD, and ^13^C NMR calculations. The new compound 12*S*-aspertetranone D (**26**) exhibited antibacterial activity against both methicillin-resistant *Staphylococcus aureus* and *Escherichia coli* with MIC values of 3.75 and 5 μg/mL, respectively.

## 1. Introduction

Marine fungi are important resources for the discovery of novel bioactive natural products and drug lead compounds [1,2,3,4,5]. Among them, *Aspergillus* fungi have been proved to be one of the most abundant novel bioactive compound producers [3,4,5]. It was reported that a total of 512 new marine-derived natural products were isolated from *Aspergillus* fungal origins from 1992 to 2014, of which 36% exhibited diverse bioactivities [3]. Recent updates indicated that 361 new secondary metabolites were identified from the *Aspergillus* fungi from 1915 to 2020. Since then, more and more novel bioactive natural products have been continuously isolated from marine-derived *Aspergillus* species, including *p*-terphenyl derivatives of asperterphenyls A-N from *A.* sp. SCSIO41315 [6], cyclopentapeptides of pseudoviridinutans A-F from *A. pseudoviridinutans* TW58-5 [7], and indoloquinazoline alkaloids of clavutoines A-U from *A. clavutus* LZD32-24 [8].

The Mariana Trench is well known for being the deepest site in the Earth’s oceans, and a number of investigations showed that the Mariana Trench is rich in microorganisms [9,10,11,12]. Previously reported metabolites from the Mariana Trench microorganisms included phenazines [13,14], aniline-tetramic acids [15], phenylbutenote and nocapyrone [16], and *n*-acetylglutaminyl glutamine amide and desferrioxamine B [17]. Obviously, the diversity of chemical structures and bioactivities of the metabolites produced by the Mariana Trench microorganisms is unclear.

**Figure 1:**
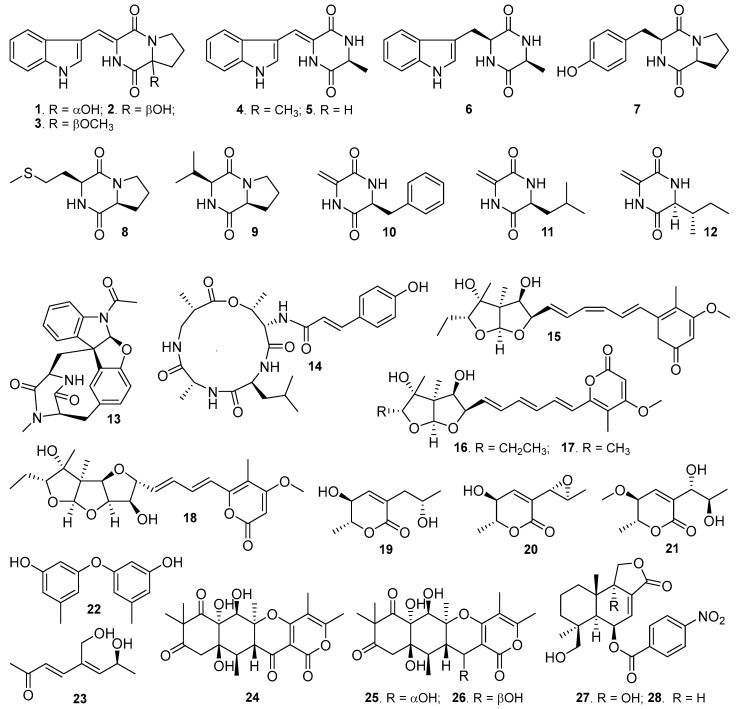
Structures of compounds **1**–**28** isolated from the marine fungus *Aspergillus* sp. SY2601.

Recently, we have conducted chemical investigations on the metabolites of several Mariana-Trench-associated microorganisms, leading to the isolation and structure elucidation of number of novel compounds, such as streptothiazolidine A, streptodiketopiperazines A and B, and (*S*)-1-(3-ethylphenyl)-1,2-ethanediol [18]. Streptothiazolidine A and streptodiketopiperazines A and B had antifungal activity against *Candida albicans* [18]. In current study, we further investigated the metabolites produced by a Mariana-Trench-derived fungus *Aspergillus* sp. SY2601 cultured in EY liquid and rice solid media, resulting in the isolation and identification of twenty-eight metabolites (**1**–**28**, Figure 1), including five new compounds, asperindopiperazine A–C (**1**–**3**), 5-methoxy-8,9-dihydroxy-8,9-deoxyaspyrone (**21**), and 12*S*-aspertetranone D (**26**). Herein, we report the culture of strain SY2601 and the isolation, structure elucidation, and bioactive evaluation of these isolated compounds.

## 2. Results and Discussion

The isolated strain SY2601 (Figure S1, Supplementary Materials) was assigned as *Aspergillus* sp. SY2601 based on its ITS rDNA sequence (Figure S2), which was an over 99.8% match to those of eleven *Aspergillus* species (Table S1). The extracts prepared from the large-scale cultures of strain SY2601 in EY liquid and rice solid media were separated by column chromatography, followed by HPLC purification, to afford compounds **1**–**28**.

Based on their NMR spectroscopic analyses, optical rotation values, co-HPLC analysis with authentic samples, and comparison to reported data, 23 known compounds were identified: 2-deisoprenyl-neoechinulin A (**4**) [19], dipodazine (**5**) [20], cyclo-L-tryptophan-L-alanine (**6**) [21], cyclo-L-proline-L-tyrosine (maculosin, **7**) [22], cyclo-L-proline-L-methionine (**8**) [22], cyclo-L-proline-L-valine (**9**) [22], (6*S*)-3-methylene-6-benzyl-2,5-piperazinedione (**10**) [23], (6*S*)-3-methylene-6-(2-methylpropyl)-2,5-piperazinedione (**11**) [24], (6*S*,8S)-3-methylene-6-(1-methylpropyl)-2,5-piperazinedione (**12**) [25], azonazine (**13**) [26], aspergillipeptide A (**14**) [27], isoasteltoxin (**15**) [28], asteltoxin (**16**) [28], asteltoxins C (**17**) and B (**18**) [29], dihydroaspyrone (**19**) [30], aspyrone (**20**) [31], diorcinol (**22**) [32], aspinonediol (**23**) [30], aspertetranones A (**24**) and D (**25**) [33], insolicolide A (**27**) [34], and 9-deoxyinsolicolide (**28**) [34]. The ^13^C and ^1^H NMR data of these known compounds are listed in Tables S2–S11 in Supplementary Materials.

**Figure 2:**
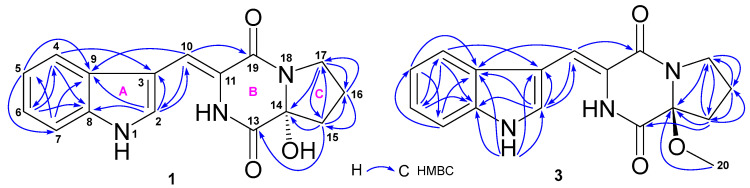
Key HMBC correlations of asperindopiperazines A (**1**) and C (**3**).

The HRESIMS spectrum of compound **1** showed ion peaks at *m*/*z* 298.1192 [M + H]^+^ (calcd. C_16_H_16_N_3_O_3_, 298.1192), 320.1011 [M + Na]^+^ (calcd. C_16_H_15_N_3_NaO_3_, 320.1011), and 617.2123 [2M + Na]^+^ (calcd. C_32_H_30_N_6_NaO_6_, 617.2125), corresponding to molecular formulate C_16_H_15_N_3_O_3_. Extensive NMR spectroscopic analyses showed that compound **1** is composed of an indole (A), 3-methylene-6-hydroxy-2,5-piperazinedione (B), and 2-hydroxypyrrolidine (C) (Figure 2) substructure. The presence of the indole group (A) was indicated by its characteristic NMR signals at *δ*_C_ 126.8 (CH, C-2), 108.1 (C, C-3), 118.1 (CH, C-4), 119.9 (CH, C-5), 122.0 (CH, C-6), 111.8 (CH, C-7), 135.7 (C, C-8), and 127.0 (C, C-9); and *δ*_H_ 11.67 (1H, s, H-1), 7.94 (1H, s, H-2), 7.66 (1H, d, 8.0 Hz, H-4), 7.10 (1H, t, 8.0 Hz, H-5), 7.16 (1H, t, 8.0 Hz, H-6), and 7.43 (1H, d, 8.0 Hz, H-7) (Table 1). Similarly, the 3-methylene-6-hydroxy-2,5-piperazinedione unit (B) was deduced from its NMR signals at *δ*_C_ 108.0 (CH, C-10), 123.7 (C, C-11), 166.1 (C, C-13), 86.5 (C, C-14), and 159.9 (C, C-19); and *δ*_H_ 7.02 (1H, s, H-10), 9.57 (1H, br s, H-12), and 6.75 (1H, br s, OH-14). The 2-hydroxypyrrolidine moiety (C) resonated at *δ*_C_ 86.5 (C, C-14), 35.7 (CH_2_, C-15), 19.4 (CH_2_, C-16), and 44.7 (CH_2_, C-17); and *δ*_H_ 2.12 (2H, m, H-15), 2.03 (1H, m, H-16a), 1.88 (1H, m, H-16b), 3.62 (1H, m, H-17a), 3.50 (1H, m, H-17b), and 6.75 (1H, br s, OH-14). As depicted in Figure 2, HMBC correlations of H-2 with C-10; H-10 with C-2, C-9, and C-19; H-15 with C-13 and C-14; and H-17 with C-14 established the linkage of the three groups. The absolute configuration at C-14, the only chiral carbon, was determined through optical rotation (OR) calculations [35,36]. The results showed a positive OR value (+85.6) for 14*R* (Table S12) and a negative OR value (–85.6) for 14*S* (Table S14). Accordingly, a 14*R* configuration was assigned for **1** because of its positive OR value (+78.7). Thus, the structure of **1** was elucidated as a new indolyl diketopiperazine, named asperindopiperazine A. Its ^13^C and ^1^H NMR data (Table 1) were assigned based on HMQC and HMBC correlations.

Compound **2** had the same molecular formulate as that of **1** based on its HRESIMS ion peaks at *m*/*z* 298.1191 [M + H]^+^, 320.1011 [M + Na]^+^, and 617.2122 [2M + Na]^+^, as well as ^13^C NMR data. Both **2** and **1** had very close UV absorptions. Detailed analysis of the ^13^C and ^1^H NMR spectra of **2** indicated that the chemical shifts of **2** bore a resemblance to those of **1**. However, compound **2** showed a negative OR value (–80.0). Thus, compound **2** should be an isomer of **1** with a 14*S* configuration. The structure of **2** was thus determined to be a new indolyl diketopiperazine, named asperindopiperazine B. Its ^13^C and ^1^H NMR data are reported in Table 1.

Compound **3** gave HRESIMS ion peaks at *m*/*z* 312.1348 [M + H]^+^ (calcd. C_17_H_18_N_3_O_3_, 312.1348), 334.1171 [M + Na]^+^ (calcd. C_17_H_17_N_3_NaO_3_, 334.1168), and 645.2427 [2M + Na]^+^ (calcd. C_34_H_34_N_6_NaO_6_, 645.2438), 14 mass units higher than those of **2** and **1**. Compound **3** also shared very similar UV absorptions as **2** and **1**, suggesting that **3** was an analogue of **2** and **1**. Detailed comparison of the ^13^C and ^1^H NMR data (Table 1) of **3** with those of **2** and **1** concluded that the chemical shifts of carbons and protons of the three compounds were almost superimposable, excepted for additional signals (*δ*_C_ 51.1; *δ*_H_ 3.16, 3H, s) for a methoxy group in **3**. HMBC correlation (Figure 2) of H-20 (*δ*_H_ 3.16) with C-14 (*δ*_C_ 91.3) established the position of this methoxy group at C-14. The downfield chemical shift (Δ 4.8 ppm) of C-14 in **3**, when compared to those in **2** and **1**, also supported the position of this methoxy group. The 14*S* configuration in **3** was assigned based on its negative OR value (–29.6). Therefore, the structure of **3** was elucidated as an analogue of compounds **2** and **1**, a new indolyl diketopiperazine, named asperindopiperazine C. The ^13^C and ^1^H NMR data of **3** are reported in Table 1.

**Figure 3:**
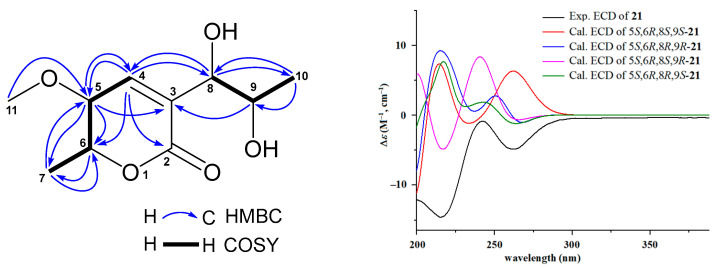
Key HMBC and COSY correlations of 5-methoxy-8,9-dihydroxy-8,9-deoxyaspyrone (**21**), the experimental ECD spectrum of 5-methoxy-8,9-dihydroxy-8,9-deoxyaspyrone (**21**), and the calculated ECD curves of the four model molecules at the b3lyp/6-311+g (d, p) level in MeOH.

Compound **21** showed HRESIMS ion peaks at *m*/*z* 217.1072 [M + H]^+^ (calcd for C_10_H_17_O_5_, 217.1076) and 239.0897 [M + Na]^+^ (calcd for C_10_H_16_NaO_5_, 239.0895), suggesting a molecular formula C_10_H_16_O_5_. Compound **21** shared similar UV absorptions with those of the known compounds **19** and **20**, implying they are aspyrone analogues. Interpretation of its ^13^C, ^1^H, HMQC, and HMBC NMR spectra indicated that **21** had one carbonyl (*δ*_C_ 163.2), two olefinic carbons (*δ*_C_ 146.2 and 128.2), four oxymethines (*δ*_C_ 82.1, 77.9, 68.0, and 66.7), one methoxy (*δ*_C_ 57.0), and two methyls (*δ*_C_ 18.5 and 17.6). Further comparison of its NMR data with those of **19** (Table S8) showed that both **21** and **19** exhibited very similar patterns of NMR chemical shifts, exception for that the methylene (*δ*_C_ 39.7; *δ*_H_ 2.44, 2.40) at C-8 in **19** was replaced by an oxymethine (*δ*_C_ 77.9; *δ*_H_ 4.15) in **21** and additional NMR signals (*δ*_C_ 57.0; *δ*_H_ 3.20) for a methoxy group were observed in the NMR spectra of **21**. HMBC correlation (Figure 3) of H-11 (*δ*_H_ 3.20) with C-5 (*δ*_C_ 82.1) determined this methoxy group at C-5 position. The absolute configurations of **21** were assigned based on the results from ECD and ^13^C NMR calculations [37,38]. Because all previously reported aspyrone analogues [30,31,39,40,41] including compounds **19** and **20** shared the same 5*S*,6*R*-configuration, only four model molecules of 5*S*,6*R*,8*S*,9*S*-**21**, 5*S*,6*R*,8*R*,9*R*-**21**, 5*S*,6*R*,8*S*,9*R*-**21**, and 5*S*,6*R*,8*R*,9*S*-**21** were applied for ECD calculations. The results (Figure 3) indicated that the experimental ECD spectrum was in agreement with the calculated ECD curve of the model molecule 5*S*,6*R*,8*S*,9*R*-**21**, suggesting a 5*S*,6*R*,8*S*,9*R*-configuration for **21**, which was further supported by the results from ^13^C NMR calculations. As shown in Table S24, the experimental ^13^C NMR data of **21** were close to those of the model molecule of 5*S*,6*R*,8S,9*R*-**21** with a DP4^+^ probability score of 98.87%. Therefore, the structure of **21** was identified as 5-methoxy-8,9-dihydroxy-8,9-deoxyaspyrone, a new member of the aspyrone family with a 5*S*,6*R*,8*S*,9*R*-configuration, which was the same as that of a reported compound of 8,9-dihydroxy-8,9-deoxyaspyrone [40,41]. The ^13^C and ^1^H NMR data (Table 2) of **21** were unambiguously assigned based on the HMQC, COSY, and HMBC correlations (Figure 3).

**Figure 4:**
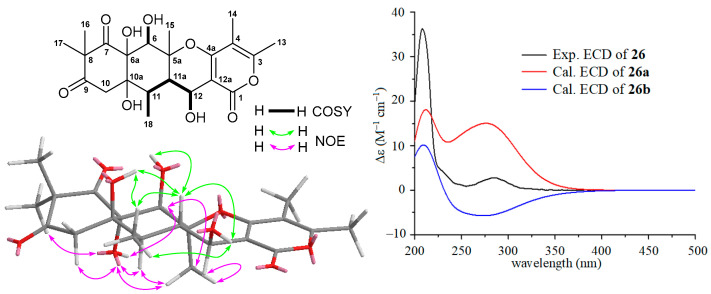
COSY and key NOE correlations of 12*S*-aspertetranone D (**26**), the experimental ECD spectrum of 12*S*-aspertetranone D (**26**), and the calculated ECD curves of the two model molecules of **26a** and **26b** at the b3lyp/6-311+g (d, p) level in MeOH.

The HRESIMS spectrum of compound **26** gave ion peaks at *m*/*z* 437.1821 [M + H]^+^ (calcd for C_22_H_29_O_9_, 437.1812) and 459.1631 [M + Na]^+^ (calcd for C_22_H_28_NaO_9_, 459.1631), corresponding to molecular formula C_22_H_28_O_9_, which is the same as that of aspertetranone D (**25**). Careful analyses of the ^1^H, ^13^C, COSY, and NOESY spectra of **26** and comparing its NMR data (Table 2) with those (Table S10) of **25** concluded that the only difference between **26** and **25** was the configuration at the C-12 position. The larger coupling constant of 9.0 Hz (^3^*J*_11a_-_12_) in **26** and the small coupling constant of 3.9 Hz (^3^*J*_11a_-_12_) in **25** suggested a *trans*-configuration between H-11a and H-12 in **26** compared to its counterpart with a *cis*-configuration in **25**. The relative configurations of **26** were further supported by NOE information. As depicted in Figure 4, NOE correlations of H-6 (*δ*_H_ 4.23) with OH-6a (*δ*_H_ 6.69) and H_3_-15 (*δ*_H_ 1.26), H-11 (*δ*_H_ 1.87) with OH-6a and H_3_-15, and H_3_-15 with OH-6a and H-12 (*δ*_H_ 4.36) suggested an α-orientation for these protons, while the β-orientations for OH-6, OH-10a, H-11a, OH-12, and H_3_-18 were indicated by NOE correlations of OH-6 (*δ*_H_ 6.62) with H-11a (*δ*_H_ 2.12), OH-10a (*δ*_H_ 4.86) with H-11a and H_3_-18 (*δ*_H_ 1.10), H-11a with OH-12 (*δ*_H_ 4.76) and H_3_-18, and OH-12 with H_3_-18. A combination of ECD and ^13^C NMR calculations was used to determine the absolute configuration of **26**. Two model molecules of 5a*S*,6*R*,6a*R*,10a*R*,11*R*,11a*S*,12*S* (**26a**) and 5a*R*,6*S*,6a*S*,10a*S*,11*S*,11a*R*,12*R* (**26b**) were applied for ECD and ^13^C NMR calculations, respectively. As shown in Figure 4, the experiment ECD spectrum of **26** was close to the calculated curve of the model molecule **26a**, indicating **26** had a 5a*S*,6*R*,6a*R*,10a*R*,11*R*,11a*S*,12*S*-configuration, which was further supported by the results of the ^13^C NMR calculations with a DP4^+^ probability score of 77.86% (Table S29). Therefore, the structure of **26** with a β-OH group at C-12, an analogue of **25**, was elucidated as a new putative meroterpenoid [33], named 12*S*-aspertetranone D. Its ^13^C and ^1^H NMR are reported in Table 2.

The antimicrobial activity of compounds **1**–**28** against methicillin-resistant *Staphylococcus aureus* (MRSA), *Escherichia coli*, and *Candida albicans* were evaluated by the micro-broth dilution method [42]. The results (Table 3) showed that new putative meroterpenoid 12*S*-aspertetranone D (**26**) exhibited antibacterial activity against both MRSA and *E. coli* with MIC values of 3.75 and 5 μg/mL, respectively. Known compound aspyrone (**20**) also had antibacterial activity, with MIC values of 40 μg/mL for MRSA and 21 μg/mL for *E. coli*; while cyclo-L-proline-L-valine (**9**), (6*S*)-3-methylene-6-(2-methylpropyl)-2,5-piperazinedione (**11**), aspergillipeptide A (**14**), and diorcinol (**22**) showed weak antifungal activity (MIC: 48–49 μg/mL) against *C. albicans*. In addition, diorcinol (**22**, 25 μg/mL) and insolicolide A (**27**, 4 μg/mL) displayed antibacterial activity against *E. coli*.

## 3. Materials and Methods

### 3.1. General Procedures

The materials for extraction, isolation, and bioactivity evaluation of compounds, and the instruments used for compound purification, optical rotation, UV, ECD, IR, NMR, and HRESIMS measurement were the same as our previous publication [18]. Culture media used in this study were prepared by the authors, including B solid medium (soluble starch 20.0 g, KNO_3_ 1.0 g, MgSO_4_⋅7H_2_O 0.5 g, NaCl 0.5 g, K_2_HPO_4_ 0.5 g, FeSO_4_ 0.01 g, agar 30.0 g, water 1 L), BY solid medium (B solid medium 1 L, sea salt 35.0 g), CA solid medium (glycerol 6 mL, arginine 1.0 g, K_2_HPO_4_ 1.0 g, MgSO_4_·7H_2_O 1.0 g, agar 30.0 g, water 1 L), CAY solid medium (CA solid medium 1 L, sea salt 35.0 g), D solid medium (potato dextrose broth 28.0 g, agar 30.0 g, water 1 L), DY solid medium (D solid medium 1 L, sea salt 35.0 g), E solid medium (yeast 1.0 g, tryptone 5.0 g, FeCl_3_⋅6H_2_O 0.17 g, KH_2_PO_4_ 0.12 g, agar 30.0 g, water 1 L), EY solid medium (E solid medium 1 L, sea salt 35.0 g), SC solid medium (peptone 5.0 g, lactose 4.0 g, Na_2_HPO_4_ 5.5 g, NaH_2_PO_4_ 4.5 g, NaHSeO_3_ 4.0 g, L-cystine 0.01 g, agar 30.0 g, water 1 L), and SCY solid medium (SC solid medium 1 L, sea salt 35.0 g).

### 3.2. Isolation and Identification of Strain SY2601

The strain SY2601 was isolated from sediment obtained from the Mariana Trench at a depth of 5842 m, as per the described procedure in our previous publication [18] by using ten different solid media (B, BY, CA, CAY, D, DY, E, EY, SC, SCY). The signal-purified colony of SY2601 was obtained on the E medium coated with a 10^–3^ g/mL sample suspension.

The strain SY2610 was identified by comparing its ITS rDNA sequence (accession number: OR646740) with the data of the GenBank. The ITS rDNA sequence analysis was performed by Legenomics (Hangzhou, China). The strain *Aspergillus* sp. SY2601 can be obtained from the Laboratory of Institute of Marine Biology and Pharmacology, Ocean College, Zhoushan Campus, Zhejiang University, China.

### 3.3. Mass Culture of Strain SY2601 in EY Liquid and Rice Solid Media

For strain SY1601 cultured in EY medium, a pure colony of the strain SY2601 picked from the E slant medium was transferred into a 250 mL EY liquid medium in a 500 mL Erlenmeyer flask and incubated at 28 °C for 3 days with shaking (180 rpm) to obtain a seed broth. The 5 mL seed broth was further transferred into 250 mL of an EY liquid culture medium in a 500 mL Erlenmeyer flask, and then statically incubated at 28 °C for 30 days. A total of 300 bottle cultures (75 L) were prepared for this study.

For strain SY2610 cultured in rice solid medium, the above prepared seed broth (5 mL) was transferred into a rice medium (40 g rice, 60 mL of 25 g/L sea salt solution) in a 500 mL Erlenmeyer flask and then incubated at 28 °C for 24 days. A total of 200 bottles of rice medium cultures were prepared for this study.

### 3.4. Extraction and Isolation of Compounds ***1**–**28***

Compounds **1**–**17** were isolated from the cultures of strain SY 2601 in EY liquid medium. The 75 L cultures of strain SY 2601 were filtered to give filtrate and mycelia. The filtrate was extracted with EtOAc three times to give EtOAc extract (2.95 g), and the mycelia were extracted with MeOH three times to give MeOH extract (20.01 g). The combination (22.96 g) of the two extracts dissolved in MeOH was mixed with silica gel (25 g). After removal of the solvent, the dried mixture was separated using a column of silica gel (350 g), successively eluting with a mixture of petroleum ether and EtOAc (10:1, 5:1,1:1, each 2 L) and a mixture of EtOAc and MeOH (10:1, 5:1, 1:1, 0:1, each 2 L) to furnish 28 fractions (Frs. 1–28, each 500 mL). Based on the results of HPLC analyses, the 28 fractions were combined into six fractions of Fr.A (Frs.1–5), Fr.B (Frs.6–9), Fr.C (Frs.10–13), Fr.D (Frs.14–17), Fr.E (Frs.18–20), and Fr.F (Frs.21–28).

Fr.D (0.7 g) was fractionated on a column of ODS (70 g), successively eluting with 30, 50, 70, and 100% MeOH (each 270 mL) to give eight subfractions (SFrs.D1–D8, each 135 mL). Compounds **10** (0.6 mg, t_R_ 37.9 min), **11** (2.2 mg, t_R_ 34.5 min), and **12** (0.5 mg, t_R_ 30.6 min) were obtained from SFr. D5 by HPLC separation using a Zorbax SB-C_18_ column (250 × 9.4 mm, 5 µm; mobile phase: ACN/H_2_O, 16/84; flow rate: 1.0 mL/min; UV detection: 210 nm).

Fr.E (5.5 g) was also fractionated on a column of ODS (150 g), successively eluting with 30, 50, 70, 85, and 100% MeOH (each 600 mL) to give 20 subfractions (Frs.1–20, each 150 mL). According to the results of HPLC analyses, different subfractions were combined into three fractions of SFr.Ea (Frs.2–3), SFr.Eb (Frs.4–6), and SFr.Ec (Frs.7–10). SFr.Ea was separated by prepared HPLC using a Fuji-C_18_ CT-30 column (280 × 30 mm, 10 µm; mobile phase: MeOH/H_2_O, 25/75, 0−49 min, 100/0, 49.01−59 min, 25/75, 59.01−69 min; flow rate: 6 mL/min; UV detection: 210 nm) to give compound **7** (10.1 mg, t_R_ 24.6 min), SFr.Ea2 (31.8 mg, t_R_ 31.2 min), SFr.Ea3 (20.8 mg, t_R_ 41.5 min), and SFr.Ea6 (6.5 mg, t_R_ 47.1 min). Further purification of SFr.Ea2, SFr.Ea3, and SFr.Ea6 was performed using the SB-C_18_ column (flow rate: 1.0 mL/min, UV detection: 210 nm) to furnish compounds **9** (11.5 mg, t_R_ 24.5 min, ACN/H_2_O, 15/85), **8** (8.8 mg, t_R_ 39.8 min, ACN/H_2_O, 10/90), and **6** (0.9 mg, t_R_ 50.0 min, ACN/H_2_O, 13/87), respectively. SFr.Ec was repeatedly separated on a column of ODS (100 g), successively eluting with 40, 50, 60, 70, and 100% MeOH (each 300 mL) to obtain 20 subfractions (Frs.1–20, each 75 mL) which were combined into four fractions of SFr.Ec1 (Frs.4–7), SFr.Ec2 (Fr.8), SFr.Ec3 (Frs. 9–10), and SFr.Ec4 (Fr. 11–15) based on the results of HPLC analyses. SFr.Ec1 was further separated on the Fuji-C_18_ CT-30 column (flow rate: 6 mL/min; mobile phase: MeOH/H_2_O, 48/52; UV detection: 210 nm) to obtain compounds **2** (2.8 mg, t_R_ 32.4 min) and **4** (10.7 mg, t_R_ 24.6 min). Using the SB-C_18_ column (flow rate: 1.0 mL/min; UV detection: 210 nm), compounds **5** (3.0 mg, t_R_ 41.8 min, ACN/H_2_O, 20/80) and **3** (2 mg, t_R_ 66.2 min, MeOH/H_2_O, 48/52) were purified from SFr.Ec2 and SFr.Ec3, respectively. Separation of SFr.Ec4 was performed using the SB-C_18_ column (flow rate: 1.0 mL/min; mobile phase: ACN/H_2_O, 34/66, UV detection: 210 nm) to give compounds **13** (8.1 mg, t_R_ 22.4 min), **17** (8.1 mg, t_R_ 40.0 min), **16** (0.4 mg, t_R_ 62.5 min), SFr.Ec4a (10.8 mg, t_R_ 27.2 min), and SFr.Ec4b (4.0 mg, t_R_ 72.4 min). Further purification of SFr.Ec4a and SFr.Ec4b used the SB-C_18_ column (flow rate: 1.0 mL/min; UV detection: 210 nm) to furnish compounds **14** (6.3 mg, t_R_ 88.1 min, ACN/H_2_O, 26/74) and **15** (1.3 mg, t_R_ 50.8 min, ACN/H_2_O, 30/70), respectively.

Similarly, Fr.F (13.1 g) was separated by a column of ODS (280 g), eluting with 30, 50, 70, and 100% MeOH (each 1080 mL) in turn to give 16 subfractions (SFr.F1–16, each 270 mL). SFr.F7 was further separated using the SB-C_18_ column (flow rate: 1.0 mL/min; mobile phase: ACN/H_2_O, 24/76; UV detection: 210 nm) to afford compound **1** (1.1 mg, t_R_ 33.1 min).

Compounds **13**, **14**, and **16**–**28** were isolated from the cultures of strain SY 2601 in rice medium. Each of the rice cultures in the 200 bottles was extracted by EtOAc three times (each 200 mL). The combined EtOAc extract solution was dried under reduced pressure to give a crude extract (100.75 g).

Initially, this crude extract was fractionated on a column of silica gel (1900 g) eluting with a mixture of petroleum ether and EtOAc (10:1, 5:1, 2:1, 1:1, each 11 L), and then a mixture of EtOAc and MeOH (0:1, 5:1, 1:1, 0:1, each 11 L) to give eight fractions (Frs.1–8, each 11 L), which were combined into five fractions of Fr.A (Frs.1–2), Fr. B (Frs.3–4), Fr. C (Fr.5), Fr. D (Fr. 6–7), and Fr.E (Fr. 8) based on the results of HPLC analyses.

Then, Fr. B (8.6 g) was further fractionated on a column of ODS (300 g), successively eluting with 25, 45, 65, and 100% MeOH (each 1.6 L) to give 16 subfractions (Frs.1–16, each 400 mL), which were further combined into three subfractions of SFr.Ba (Frs.1–2), SFr.Bb (Fr.3), and SFr.Bc (Fr.12). By using the SB-C18 column (flow rate: 1.0 mL/min; UV detection: 210 nm), compounds **20** (6.2 mg, t_R_ 25.3 min, ACN/H_2_O, 20/80), **21** (3.6 mg, t_R_ 29.3 min, MeOH/H_2_O, 24/76), and **22** (8.2 mg, t_R_ 49.4 min, MeOH/H_2_O, 60/40) were purified from SFr.Bb, SFr.Ba, and SFr.Bc, respectively.

Next, Fr. C (6.12 g) was also fractionated on the column of ODS (300 g), successively eluting with 30, 50, 70, 95, and 100% MeOH (each 1.4 L) to give 35 subfractions (Frs. 1–35, each 200 mL), which were combined into five subfractions of SFr.Ca (Fr. 3), SFr.Cb (Frs.12–15), SFr.Cc (Fr.16), SFr.Cd (Fr.17), and SFr.Ce (Fr.18) based on the results of HPLC analyses. Using the SB-C18 column (flow rate: 1.0 mL/min, UV detection: 210 nm), compounds **19** (4.5 mg, t_R_ 39.0 min, MeOH/H_2_O, 20/80) and **28** (2.3 mg, t_R_ 53.5 min, ACN/H_2_O, 35/65–60/40, 0–50 min, 100/0, 50.01–60 min) were purified from SFr.Ca and SFr.Ce, respectively; **25** (2.2 mg, t_R_ 38.4 min), **24** (9.6 mg, t_R_ 44.2 min), and **26** (6.7 mg, t_R_ 49.5 min, ACN/H_2_O, 25/75) from SFr.Cb; **16** (3.6 mg, t_R_ 46.2 min) and **27** (3.8 mg, t_R_ 64.3 min, ACN/H_2_O, 37/63) from SFr.Cc; and **17** (3.4 mg, t_R_ 33.7 min) and **18** (1.6 mg, t_R_ 31.4 min, ACN/H_2_O, 36/64) from SFr.Cd.

Finally, Fr.D (10.95 g) was fractionated on the column of ODS (300 g), successively eluting with 30, 50, 70, 90, and 100% MeOH (each 1.2 L) to give 20 subfractions (Frs.1–20, each 300 mL), which were combined into two subfractions of SFr.Da (Frs.1–3) and SFr.Db (Frs.9–11) based on the results of HPLC analyses. By HPLC purification on the SB-C18 column (flow rate:1.0 mL/min; UV detection: 210 nm), compound **23** (1.2 mg, t_R_ 43.9 min, MeOH/H_2_O, 20/80) was obtained from SFr.Da, and compounds **13** (2.1 mg, t_R_ 41.9 min) and **14** (1.6 mg, t_R_ 54.9 min, ACN/H_2_O, 28/72) were obtained from SFr.Db.

Asperindopiperazine A (**1**): White amorphous powder; molecular formula C_16_H_15_N_3_O_3_; [α]^20^_D_ +78.7° (*c* 0.1, MeOH); UV (MeOH) λ_max_ (log ε) 210 (4.78), 346 (4.43) nm; IR (ATR) ν_max_ 3387, 3051, 1690, 1651, 1622, 1489, 1457, 1395, 1232, 1185, 1133, 1083, 744 cm^–1^; ^13^C and ^1^H NMR data, see Table 1; HRESIMS *m*/*z* 298.1192 [M + H]^+^ (calcd. C_16_H_16_N_3_O_3_, 298.1192), 320.1011 [M + Na]^+^ (calcd. C_16_H_15_N_3_NaO_3_, 320.1011), 617.2123 [2M + Na]^+^ (calcd. C_32_H_30_N_6_NaO_6_, 617.2125).

Asperindopiperazine B (**2**): White amorphous powder; molecular formula C_16_H_15_N_3_O_3_; [α]^20^_D_ –80.0° (*c* 0.1, MeOH); UV (MeOH) λ_max_ (log ε) 210 (4.64), 350 (3.95) nm; IR (ATR) ν_max_ 3312, 2960, 1682, 1666, 1620, 1545, 1423, 1339, 1236, 1176, 1114, 1042, 755 cm^–1^; ^13^C and ^1^H NMR data, see Table 1; HRESIMS *m*/*z* 298.1191 [M + H]^+^ (calcd. C_16_H_16_N_3_O_3_, 298.1192), 320.1011 [M + Na]^+^ (calcd. C_16_H_15_N_3_NaO_3_, 320.1011), 617.2122 [2M + Na]^+^ (calcd. C_32_H_30_N_6_NaO_6_, 617.2125).

Asperindopiperazine C (**3**): White amorphous powder; molecular formula C_17_H_17_N_3_O_3_; [α]^20^_D_ –29.6° (*c* 0.1, MeOH); UV (MeOH) λ_max_ (log ε) 213 (4.64), 350 (4.17) nm; IR (ATR) ν_max_ 3274, 2960, 1686, 1653, 1616, 1530, 1431, 1388, 1240, 1184, 1137, 1109, 1056, 748 cm^–1^; ^13^C and ^1^H NMR data, see Table 1; HRESIMS *m*/*z* 312.1348 [M + H]^+^ (calcd. C_17_H_18_N_3_O_3_, 312.1348), 334.1171 [M + Na]^+^ (calcd. C_17_H_17_N_3_NaO_3_, 334.1168), 645.2427 [2M + Na]^+^ (calcd. C_34_H_34_N_6_NaO_6_, 645.2438).

5-Methoxy-8,9-dihydroxy-8,9-deoxyaspyrone (**21**): colorless oil; molecular formula C_10_H_16_O_5_; [α]^20^_D_ –80.4° (*c* 0.1, MeOH); UV (MeOH) λ_max_ (log ε) 225 (3.57) nm; ECD (*c* 1 mg/mL, MeOH) λ_max_ (Δε) 216 (–14.59), 242 (–0.87), 262 (–4.84) nm; IR (ATR) ν_max_ 3381, 2985, 2937, 1704, 1652, 1460, 1386, 1216, 1203, 1135, 1064 cm^–1^; ^13^C and ^1^H NMR data, see Table 2; HRESIMS *m*/*z* 217.1072 [M + H]^+^ (calcd for C_10_H_17_O_5_, 217.1076), 239.0897 [M + Na]^+^ (calcd for C_10_H_16_NaO_5_, 239.0895).

12*S*-Aspertetranone D (**26**): White crystal; molecular formula C_22_H_28_O_9_; [α]^20^_D_ +75.2° (*c* 0.1, MeOH); UV (MeOH) λ_max_ (log ε) 205 (5.10), 290 (3.98) nm; ECD (*c* 1 mg/mL, MeOH) λ_max_ (Δε) 208 (+36.28), 286 (+2.79) nm; IR (ATR) ν_max_ 3421, 2989, 2944, 1696, 1674, 1574, 1457, 1432, 1387, 1248, 1128, 1067, 1038, 996 cm^–1^; ^13^C and ^1^H NMR data, see Table 2; HRESIMS *m*/*z* 437.1821 [M + H]^+^ (calcd for C_22_H_29_O_9_, 437.1812), 459.1631 [M + Na]^+^ (calcd for C_22_H_28_NaO_9_, 459.1631).

### 3.5. Optical Rotation Calculations

Optical rotation (OR) calculations were conducted as per our previously described method [36].

### 3.6. ECD Calculations

The previously described method [18] was used for ECD calculations.

### 3.7. ^13^C NMR Calculations

^13^C NMR calculations were carried out referring to our previous publications [18].

### 3.8. Antimicrobial Activity Assay

The micro-broth dilution method as described in the previous study [42] was applied to determine the antimicrobial activities of all tested compounds against the growth of methicillin-resistant *Staphylococcus aureus* (MRSA), *Escherichia coli*, and *Candida albicans*. Gentamicin and amphotericin B were used as positive controls.

## 4. Conclusions

Chemical investigation on the metabolites produced by the Mariana-Trench-derived fungus *Aspergillus* sp. SY2601 in both EY liquid and rice solid media resulted in the isolation and identification of twenty-eight metabolites, including five new compounds, asperindopiperazines A–C (**1**–**3**), 5-methoxy-8,9-dihydroxy-8,9-deoxyaspyrone (**21**), and 12*S*-aspertetranone D (**26**). 12*S*-aspertetranone D (**26**) had activity in inhibiting the growth of methicillin-resistant *S. aureus* and *E. coli*. The twenty-eight isolated compounds belong to different structural types of indolyl diketopiperazines, diketopiperazines, merosesquiterpenoids, putative meroterpenoid, nitrobenzoyl sesquiterpenoids, peptides, aspyrones, and phenols. The data obtained from current study show that the fungus *Aspergillus* sp. SY2601 is able to produce abundance metabolites, which enriched the diversity of structures and bioactivities of the metabolites produced by the Mariana-Trench-associated microorganisms. 

## Figures and Tables

**Table 1:** ^13^C and ^1^H NMR data of asperindopiperazines A–C (**1**–**3**) (in DMSO-*d*_6_).

No.	1		2		3	
	*δ*_C_, Type	*δ*_H_, Multi. (*J* in Hz)	*δ*_C_, Type	*δ*_H_, Multi. (*J* in Hz)	*δ*_C_, Type	*δ*_H_, Multi. (*J* in Hz)
1	–	11.67, br s	–	11.69, br s	–	11.73, br s
2	126.8, CH	7.94, s	126.8, CH	7.95, s	127.2, CH	7.98, s
3	108.1, C	–	108.1, C	–	107.9, C	–
4	118.1, CH	7.66, d (8.0)	118.1, CH	7.66, d (8.0)	118.1, CH	7.66, d (8.0)
5	119.9, CH	7.10, t (8.0)	119.9, CH	7.10, t (8.0)	120.0, CH	7.11, t (8.0)
6	122.0, CH	7.16, t (8.0)	122.0, CH	7.16, t (8.0)	122.1, CH	7.17, t (8.0)
7	111.8, CH	7.43, d (8.0)	111.8, CH	7.43, d (8.0)	111.9, CH	7.43, d (8.0)
8	135.7, C	–	135.6, C	–	135.7, C	–
9	127.0, C	–	127.0, C	–	126.9, C	–
10	108.0, CH	7.02, s	108.0, CH	7.02, s	109.7, CH	7.07, s
11	123.7, C	–	123.7, C	–	123.0, C	–
12	–	9.57, br s	–	9.57, br s	–	9.94, br s
13	166.1, C	–	166.1, C	–	163.3, C	–
14	86.5, C	–	86.5, C	–	91.3, C	–
15	35.7, CH_2_	2.12, m	35.7, CH_2_	2.12, m	33.0, CH_2_	2.29, m; 2.06, m
16	19.4, CH_2_	2.03, m; 1.88, m	19.4, CH_2_	2.02, m; 1.89, m	19.3, CH_2_	1.91, m
17	44.7, CH_2_	3.62, m; 3.50, m	44.7, CH_2_	3.62, m; 3.50, m	45.2, CH_2_	3.63, m; 3.59, m
19	159.9, C	–	159.8, C	–	160.0, C	–
20	–	–	–	–	51.1, CH_3_	3.16, s
OH-14	–	6.75, br s	–	6.76, br s	–	–

**Table 2:** ^13^C and ^1^H NMR data of 5-methoxy-8,9-dihydroxy-8,9-deoxyaspyrone (**21**) and 12*S*-aspertetranone D (**26**) (in DMSO-*d*_6_).

No.	21		No.	26		No.	26	
	*δ*_C_, Type	*δ*_H_, Mult. (*J* in Hz)		*δ*_C_, Type	*δ*_H_, Mult. (*J* in Hz)		*δ*_C_, Type	*δ*_H_, Mult. (*J* in Hz)
2	163.2, C	–	1	162.3, C	–	11a	39.6, CH	2.12, dd (11.8, 9.0)
3	128.2, C	–	3	157.1, C	–	12	62.5, CH	4.36, dd (9.0, 4.3)
4	146.2, CH	6.64, s	4	106.5, C	–	12a	102.2, C	–
5	82.1, CH	3.90, d (7.5)	4a	162.1, C	–	13	17.0, CH_3_	2.18, s
6	68.0, CH	3.62, dq (7.5, 6.3)	5a	83.7, C	–	14	9.1, CH_3_	1.85, s
7	18.5, CH_3_	0.99, d (6.3)	6	72.5, CH	4.23, d (5.4)	15	17.5, CH_3_	1.26, s
8	77.9, CH	4.15, br s	6a	75.2, C	–	16	23.3, CH_3_	1.23, s
9	66.7, CH	4.15, br s	7	207.5, C	–	17	25.2, CH_3_	1.28, s
10	17.6, CH_3_	1.33, d (4.3)	8	54.4, C	–	18	10.8, CH_3_	1.10, d (6.5)
11	57.0, CH_3_	3.20, s	9	209.4, C	–	OH-6	–	6.62, d (5.4)
			10	45.2, CH_2_	αH: 2.65, dd (16.9, 1.9); βH: 2.57, d (16.9)	OH-6a	–	6.69, s
			10a	75.2, C	–	OH-10a	–	4.86, d (1.9)
			11	38.7, CH	1.87, dd (11.8, 6.5)	OH-12	–	4.76, d (4.3)

**Table 3:** Antimicrobial activity of tested compounds (MIC: μg/mL).

Compounds	MRSA	*E. coli*	*C. albicans*
**9**	NA	NA	49
**11**	NA	NA	49
**14**	NA	NA	48
**20**	40	21	NA
**22**	NA	25	48
**26**	3.75	5	NA
**27**	NA	4	NA
Gentamicin	0.78	0.40	NT
amphotericin B	NT	NT	0.03

NA: No activity at a concentration of 50 μg/mL; NT: No active test.

## Data Availability

All the data in this research are presented in the manuscript and Supplementary Material.

## Supplementary Materials

The following supporting information can be downloaded at: https://www.mdpi.com/article/10.3390/molecules29020459/s1. Figure S1: Colonies of fungus *Aspergillus* sp. SY2601 cultured in E medium; Figure S2: ITS rDNA sequence of *Aspergillus* sp. SY2601; Figures S3–S63: NMR, HRESIMS, IR, and UV spectra of asperindopiperazines A–C (**1**–**3**), 5-methoxy-8,9-dihydroxy-8,9-deoxyaspyrone (**21**), and 12*S*-aspertetranone D (**26**); Table S1: Sequences producing significant alignments of *Aspergillus* sp. strain SY2601; Tables S2–S11: ^13^C and ^1^H NMR data of known compounds **4**–**20**, **22**–**25**, **27**, and **28**; Tables S12–S15: Data of optical rotation calculations for asperindopiperazine A (**1**); Tables S16–S23: Data of ECD calculations for 5-methoxy-8,9-dihydroxy-8,9-deoxyaspyrone (**21**); Table S24: Data of ^13^C NMR calculations for 5-methoxy-8,9-dihydroxy-8,9-deoxyaspyrone (**21**); Tables S25–S28: Data of ECD calculations for 12*S*-aspertetranone D (**26**); Table S29: Data of ^13^C NMR calculations for 12*S*-aspertetranone D (**26**).
