# Supplementary material for: Isolation and Structure Elucidation of New Metabolites from the Mariana-Trench-Associated Fungus Aspergillus sp. SY2601
Source: Molecules. 2024 Jan 17;29(2):459. doi: 10.3390/molecules29020459 (PMC10819015; doi:10.3390/molecules29020459)
Supplement: Supplementary file 1 [file molecules-29-00459-s001.zip › molecules-2770160-supplementary.pdf]

SUPPLEMENTARY MATERIALS

Isolation and Structure Elucidation of New Metabolites from the Mariana-Trench-Associated Fungus *Aspergillus* sp. SY2601

Cangzhu Sun[1], Yura Ha[1], Xin Liu[1], Nan Wang[1,2,*], Xiao-Yuan Lian[3] and Zhizhen Zhang[1,*]

[1] Ocean College, Zhoushan Campus, Zhejiang University, Zhoushan 316021, China;

[2] Hainan Institute of Zhejiang University, Sanya 572025, China.

[3] College of Pharmaceutical Sciences, Zhejiang University, Hangzhou 310058, China

* Correspondence: n_wang@zju.edu.cn (N. W.); zzhang88@zju.edu.cn (Z.Z.); Tel: +86-13675859706 (Z.Z).

Content

Figure S1. Colonies of fungus *Aspergillus* sp. SY260l cultured in E medium……..………..…...……….4
Figure S2. ITS rDNA sequence of *Aspergillus* sp. SY2601…..…..4
Figures S3–S5. ^{1}H NMR spectrum of asperindopiperazine A (**1**)………..……………….……..……….…5
Figures S6–S8. ^{13}C NMR spectrum of asperindopiperazine A (**1**)………...6
Figures S9–S11. HMQC spectrum of asperindopiperazine A (**1**) ……………………….......................8
Figures S12–S14. HMBC spectrum of asperindopiperazine A (**1**) ………..…………………………..………9
Figure S15. HRESIMS spectrum of asperindopiperazine A (**1**) ………..…….……………………..……11
Figure S16. IR spectrum of asperindopiperazine A (**1**) ………..……………………...………………11
Figure S17. UV spectrum of asperindopiperazine A (**1**) ………..……………..………..……………11
Figures S18–S20. ^{1}H NMR spectrum of asperindopiperazine B (**2**) ………..………………………….…12
Figures S21–S23. ^{13}C NMR spectrum of asperindopiperazine B (**2**) ………...…………….…………...…13
Figure S24. HRESIMS spectrum of asperindopiperazine B (**2**) ………..………………………………..…15
Figure S25. IR spectrum of asperindopiperazine B (**2**) ………..………………………..…..…………15
Figure S26. UV spectrum of asperindopiperazine B (**2**) ………..……………………………………..…15
Figures S27–S29. ^{1}H NMR spectrum of asperindopiperazine C (**3**) ………..……………………………16
Figures S30–S32. ^{13}C NMR spectrum of asperindopiperazine C (**3**) ………..……………………………17
Figures S33–S35. HMQC spectrum of asperindopiperazine C (**3**) ………...………………………...…19
Figures S36–S38. HMBC spectrum of asperindopiperazine C (**3**) ……………...…………………………20
Figure S39. HRESIMS spectrum of asperindopiperazine C (**3**) ………..……………….……………..…22
Figure S40. IR spectrum of asperindopiperazine C (**3**) ………..………………………...………………22
Figure S41. UV spectrum of asperindopiperazine C (**3**)………………..…………………..……………22
Figures S42–S43. ^{1}H NMR spectrum of 5-methoxy-8,9-dihydroxy-8,9-deoxyaspyrone (**21**) ………...…23
Figure S44. ^{13}C NMR spectrum of 5-methoxy-8,9-dihydroxy-8,9-deoxyaspyrone (**21**) ……..……..……24
Figure S45. HMQC spectrum of 5-methoxy-8,9-dihydroxy-8,9-deoxyaspyrone (**21**) ……….........……24
Figure S46. COSY spectrum of 5-methoxy-8,9-dihydroxy-8,9-deoxyaspyrone (**21**) ……………...……25
Figures S47–S49. HMBC spectrum of 5-methoxy-8,9-dihydroxy-8,9-deoxyaspyrone (**21**) ………. ……25
Figure S50. HRESIMS spectrum of 5-methoxy-8,9-dihydroxy-8,9-deoxyaspyrone (**21**) ..… ……..……27
Figure S51. IR spectrum of 5-methoxy-8,9-dihydroxy-8,9-deoxyaspyrone (**21**) ..………………………27
Figure S52. UV spectrum of 5-methoxy-8,9-dihydroxy-8,9-deoxyaspyrone (**21**) ..………………..… …27
Figures S53–S55. ^{1}H NMR spectrum of 12*S*-aspertetranone D (**26**) …………v…..………..……………28
Figures S56–S58. ^{13}C NMR spectrum of 12*S*-aspertetranone D (**26**) ……………..………………...….…29
Figures S59–S60. COSY spectrum of 12*S*-aspertetranone D (**26**) ……………..……...………………...…31
Figure S61. HRESIMS spectrum of 12*S*-aspertetranone D (**26**) ………………..……………….………32
Figure S62. IR spectrum of 12*S*-aspertetranone D (**26**) ………………..…………….……………..……32
Figure S63. UV spectrum of 12*S*-aspertetranone D (**26**) …………………………………………...……32
Table S1. Sequences producing significant alignments of *Aspergillus* sp. strain SY2601……………..…33
Table S2. ^{13}C and ^{1}H NMR data (150 and 600 MHz) of 2-deisoprenyl-neoechinulin A (**4**), dipodazine (**5**), and cyclo-L-tryptophan-L-alanine (**6**) ……………………………………………………..……34
Table S3. ^{13}C NMR data (150 MHz) of cyclo-L-proline-L-tyrosine (7), cyclo-L-proline-L-methionine (**8**), cyclo-L-proline-L-valine (**9**), (6*S*)-3-methylene-6-benzyl-2,5-piperazinedione (**10**), (6*S*)-3-methylene-6-(2-methylpropyl)-2,5-piperazinedione (**11**), and (6*S,8S*)-3-methylene-6-(1-methylpropyl)-2,5-piperazinedione (**12**) …………………………………………………………35
Table S4. ^{1}H NMR data (600 MHz) of cyclo-L-proline-L-tyrosine (7), cyclo-L-proline-L-methionine (**8**),

cyclo-L-proline-L-valine (**9**), (6*S*)-3-methylene-6-benzyl-2,5-piperazinedione (**10**), (6*S*)-3-methylene-6-(2-methylpropyl)-2,5-piperazinedione (**11**), and (6*S,8S*)-3-methylene-6-(1-methylpropyl)-2,5-piperazinedione (**12**) ……36
Table S5. ^{13}C and ^{1}H NMR data (150 and 600 MHz, in DMSO-d_6) of azonazine (**13**) and aspergillipeptide A (**14**) ……37
Table S6. ^{13}C NMR data (150 MHz) of isoasteltoxin (**15**), asteltoxin (**16**), and asteltoxins C (**17**) and B (**18**) ……38
Table S7. ^{1}H NMR data (600 MHz) of isoasteltoxin (**15**), asteltoxin (**16**), and asteltoxins C (**17**) and B (**18**) ……39
Table S8. ^{13}C and ^{1}H NMR data (150 and 600 MHz) of dihydroaspyrone (**19**) and aspyrone (**20**) ……39
Table S9. ^{13}C and ^{1}H NMR data (150 and 600 MHz) of diorcinol (**22**) and aspinonediol (**23**) (in DMSO-d_6) ……40
Table S10. ^{13}C and ^{1}H NMR data (150 and 600 MHz) of aspertetranones A (**24**) and D (**25**) ……41
Table S11. ^{13}C and ^{1}H NMR data (150 and 600 MHz) of insolicolide A (**27**) and 9-deoxyinsolicolide (**28**) ……42
Table S12. Gibbs free energies and equilibrium populations of low-energy conformers of 14*R*-**1**……42
Table S13. Cartesian coordinates for the low-energy reoptimized MMFF conformers of 14*R*-**1** at B3LYP/6-311+G (d, p) level of theory in MeOH……42
Table S14. Gibbs free energies and equilibrium populations of low-energy conformers of 14*S*-**1**……48
Table S15. Cartesian coordinates for the low-energy reoptimized MMFF conformers of 14*S*-**1** at B3LYP/6-311+G (d, p) level of theory in MeOH……48
Table S16. Gibbs free energies and equilibrium populations of low-energy conformers of 5*S*,6*R*,8*S*,9*S*-**21**……51
Table S17. Cartesian coordinates for the low-energy reoptimized MMFF conformers of 5*S*,6*R*,8*S*,9*S*-**21** at B3LYP/6-31+G (d, p) level of theory in MeOH……51
Table S18. Gibbs free energies and equilibrium populations of low-energy conformers of 5*S*,6*R*,8*R*,9*R*-**21**……52
Table S19. Cartesian coordinates for the low-energy reoptimized MMFF conformers of 5*S*,6*R*,8*R*,9*R*-**21** at B3LYP/6-31+G (d, p) level of theory in MeOH……52
Table S20. Gibbs free energies and equilibrium populations of low-energy conformers of 5*S*,6*R*,8*S*,9*R*-**21**……56
Table S21. Cartesian coordinates for the low-energy reoptimized MMFF conformers of 5*S*,6*R*,8*S*,9*R*-21 at B3LYP/6-31+G (d, p) level of theory in MeOH……59
Table S22. Gibbs free energies and equilibrium populations of low-energy conformers of 5*S*,6*R*,8*R*,9*S*-**21**……59
Table S23. Cartesian coordinates for the low-energy reoptimized MMFF conformers of 5*S*,6*R*,8*R*,9*S*-**21** at B3LYP/6-31+G (d, p) level of theory in MeOH……62
Table S24. Experimental ^{13}C NMR data of **21** and calculated ^{13}C NMR data of 5*S*,6*R*,8*S*,9*S*-**21**, 5*S*,6*R*,8*R*,9*R*-**21**, 5*S*,6*R*,8*S*,9*R*-**21**, and 5*S*,6*R*,8*R*,9*S*-**21** and the results analyzed by the improved probability DP4^{+} method based on their ^{13}C NMR data (ppm)……63
Table S25. Gibbs free energies and equilibrium populations of low-energy conformers of **26a**……63
Table S26. Cartesian coordinates for the low-energy reoptimized MMFF conformers of **26a** at B3LYP/6-

31+G (d, p) level of theory in MeOH……………………………………………………………67
Table S27. Gibbs free energies and equilibrium populations of low-energy conformers of **26b**…………67
Table S28. Cartesian coordinates for the low-energy reoptimized MMFF conformers of **26b** at B3LYP/6-31+G (d, p) level of theory in MeOH………………………………………………………….…69
Table S29. Experimental ^{13}C NMR data of **26** and calculated ^{13}C NMR data of **26a** and **26b** and the results analyzed by the improved probability $DP4^{+}$ method based on their ^{13}C NMR data (ppm)……………70

Figure S1. Colonies of fungus *Aspergillus* sp. SY2601cultured in E medium

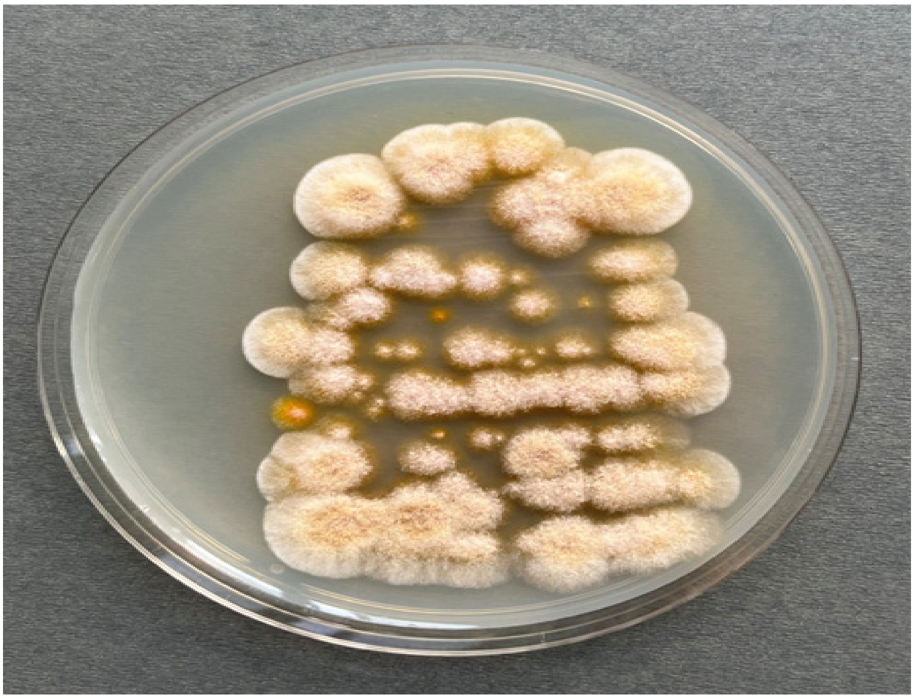

Figure S2. ITS rDNA sequence of *Aspergillus* sp. SY2601

TCTCCGGCGCGCTCGCCCGGCCCGAGGGGTTCGCCCCCGGCGGCCGCGCGAACGGCGGGCCCGCC
GAAGCAACTTGGTACAGTATACAAGGGTGGGAGGTTGGGCCCCGAAGGAACCCTCACTCAGTAATGAT
CCTTCCGTAGGGGAACCTGCGGAAGGATCATTACTGAGTGAGGGTTCCTTCGGGGCCCAACCTCCCAC
CCTTGTATACTGTACCAAGTTGCTTCGGCGGGCCCGCCGTTCGCGCGGCCGCCGGGGGGGAACCCCTC
CCCCCGGGCGAGCGCCCGCCGGAGACCCCAACGTGAACACTGTCTGAAGTTTTGTCGTCTGAGTTCGA
TTGTATCGCAATCAGTTAAAACTTTCAACAATGGATCTCTTGGTTCCGGCATCGATGAAGAACGCAGCG
AAATGCGATAATTAATGTGAATTGCAGAATTCAGTGAATCATCGAGTCTTTGAACGCACATTGCACCCCC
TGGTATTCCGGGGGGTATGCCTGTCCGAGCGTCATTGCTGCCCTCAAGCCCGGCTTGTGTGTTGGGTCC
TCGTCCCCCCCGGGGGACGGGCCCGAAAGGCAGCGGCGGCACCGCGTCCGGTCCTCGAGCGTATGGG
GCTTTGTCACACCGCTCTCGTAGGCCCGGCCGGCGCTGGCCGACGCTGAAAAGCAACCATTATTTCTCC
AGGTTGACCTCGGATCAGGTAGGGATACCCGCTGAA (bp719)

Figure S3. ^{1}H NMR spectrum of asperindopiperazine A (**1**)

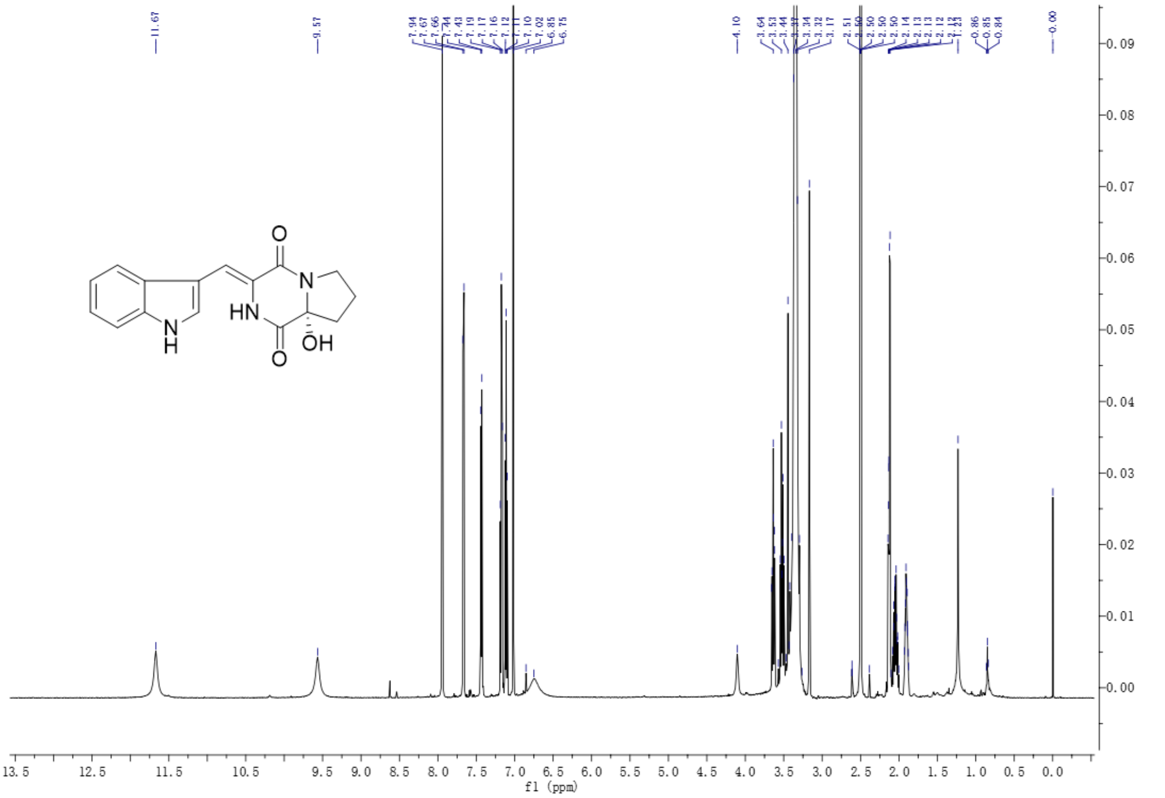

Figure S4. ^{1}H NMR spectrum of asperindopiperazine A (**1**)

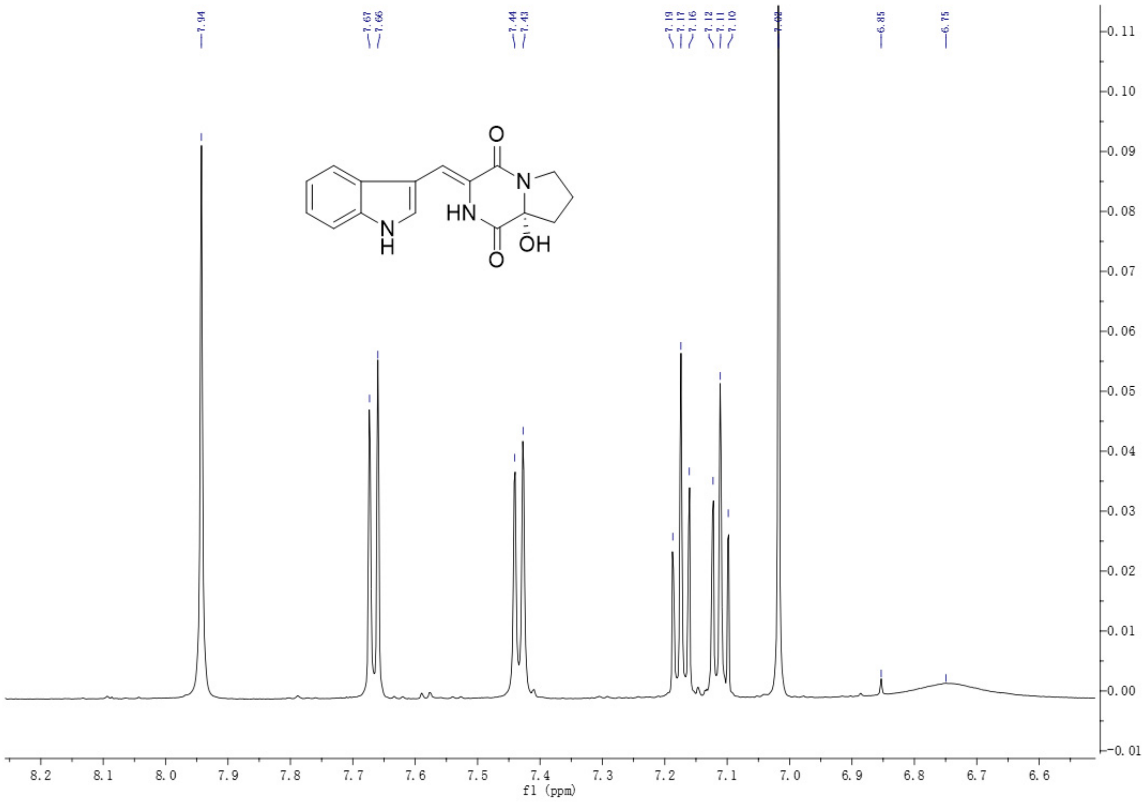

Figure S5. ^{1}H NMR spectrum of asperindopiperazine A (**1**)

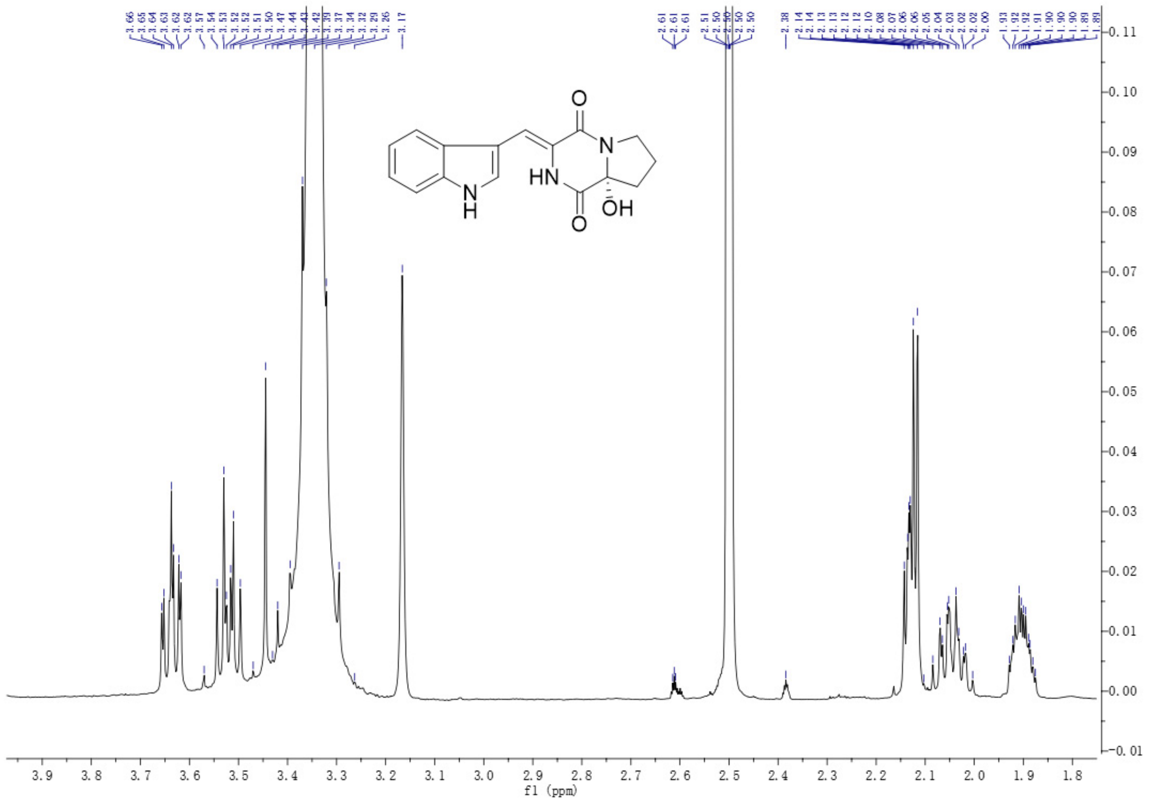

Figure S6. ^{13}C NMR spectrum of asperindopiperazine A (**1**)

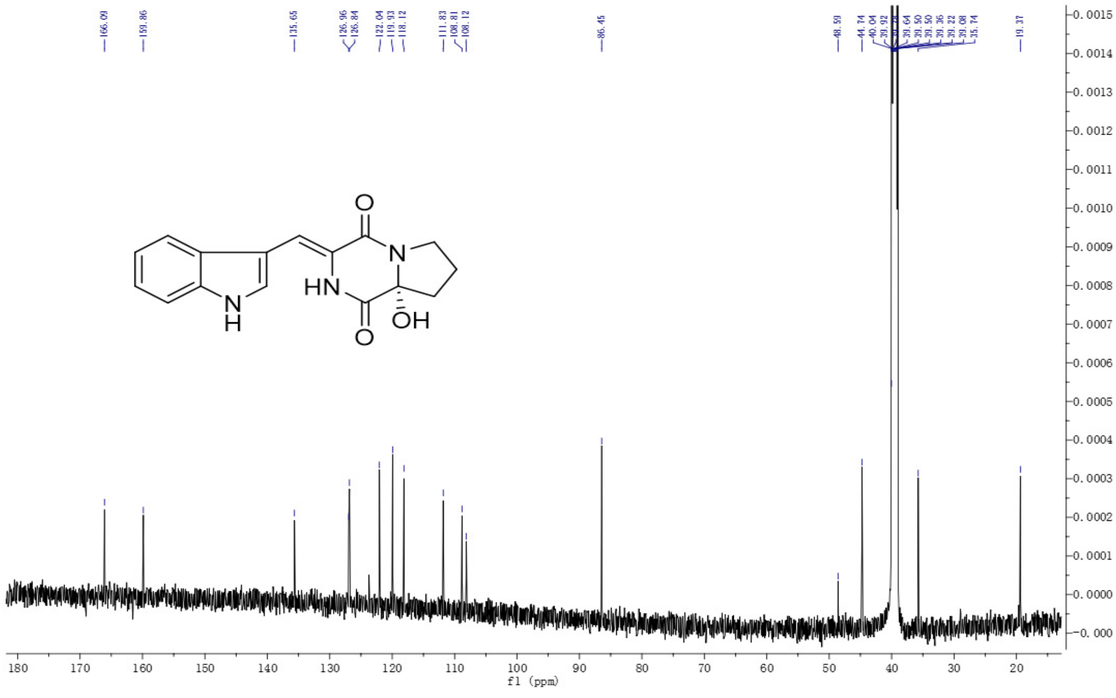

Figure S7. ^{13}C NMR spectrum of asperindopiperazine A (**1**)

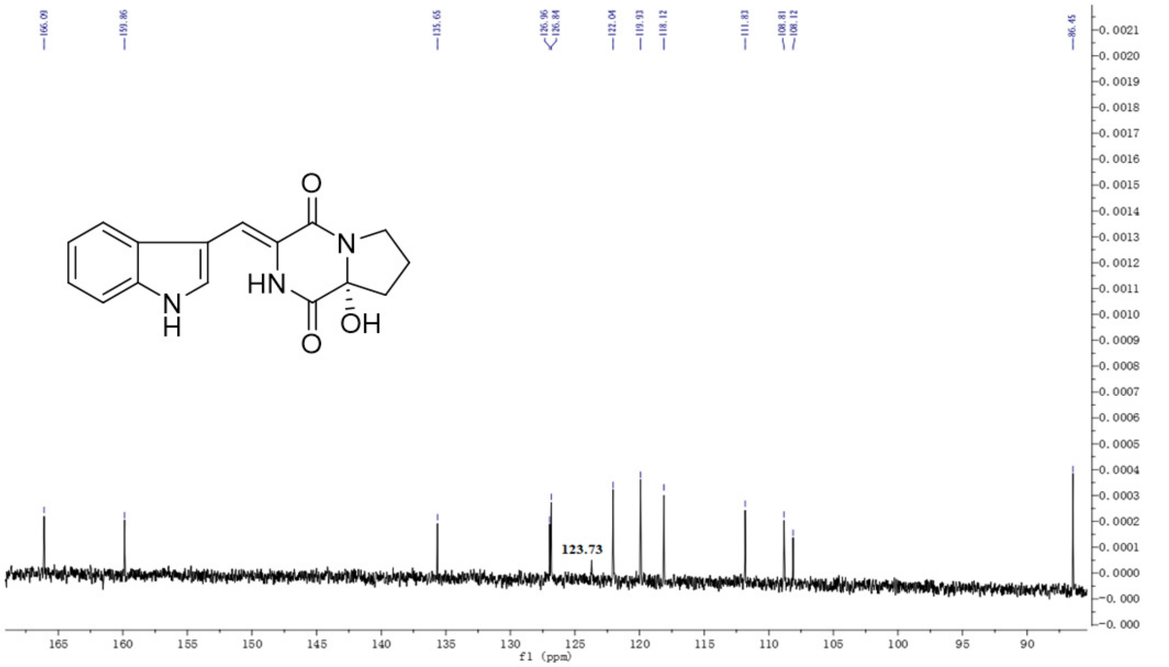

Figure S8. ^{13}C NMR spectrum of asperindopiperazine A (**1**)

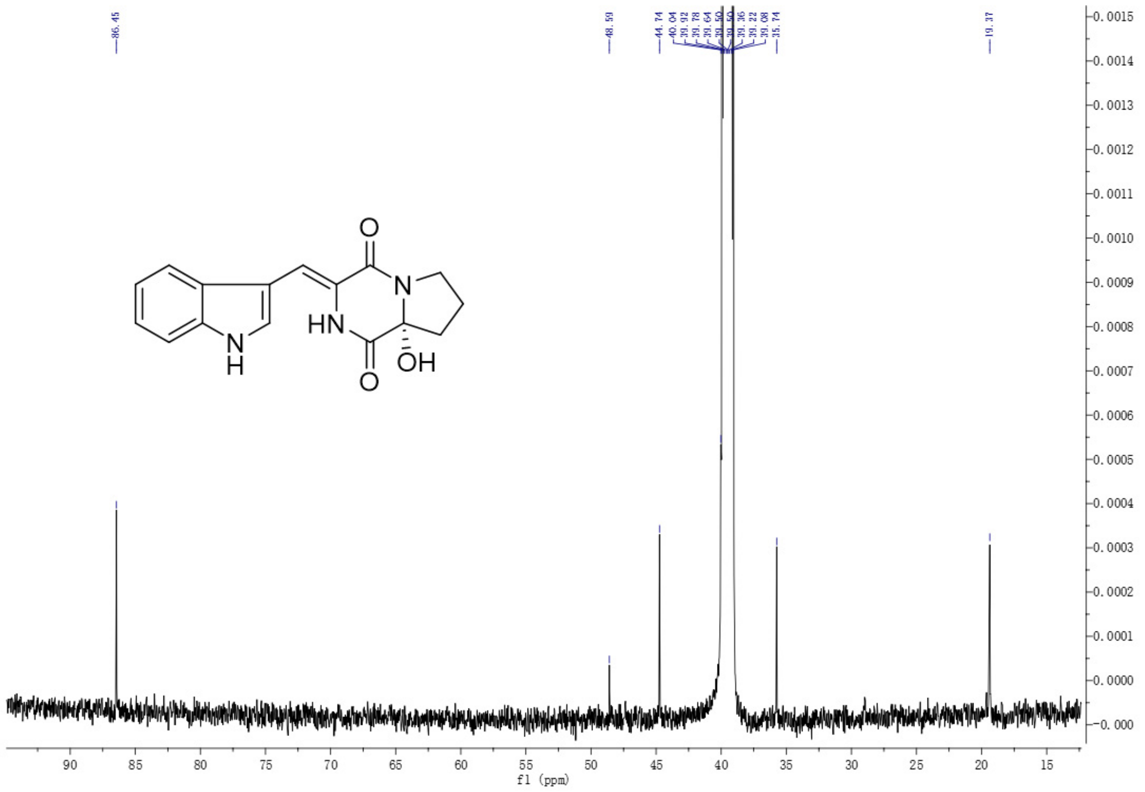

Figure S9. HMQC spectrum of asperindopiperazine A (**1**)

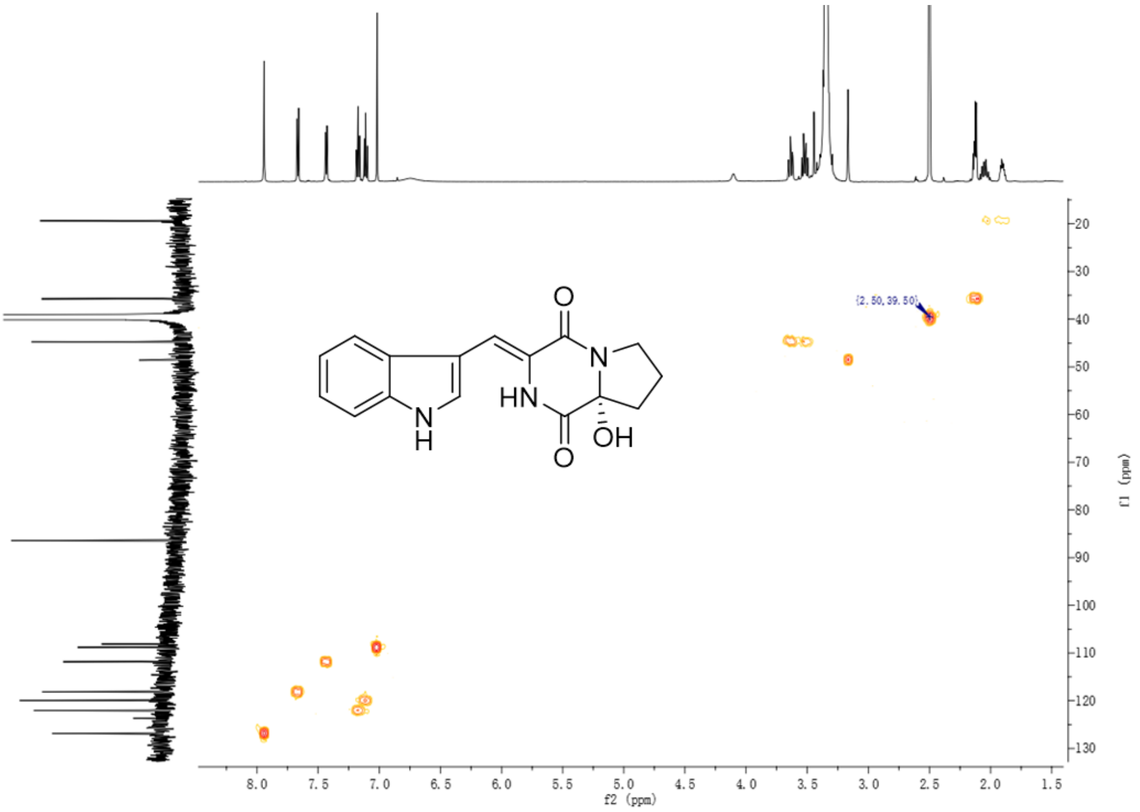

Figure S10. HMQC spectrum of asperindopiperazine A (**1**)

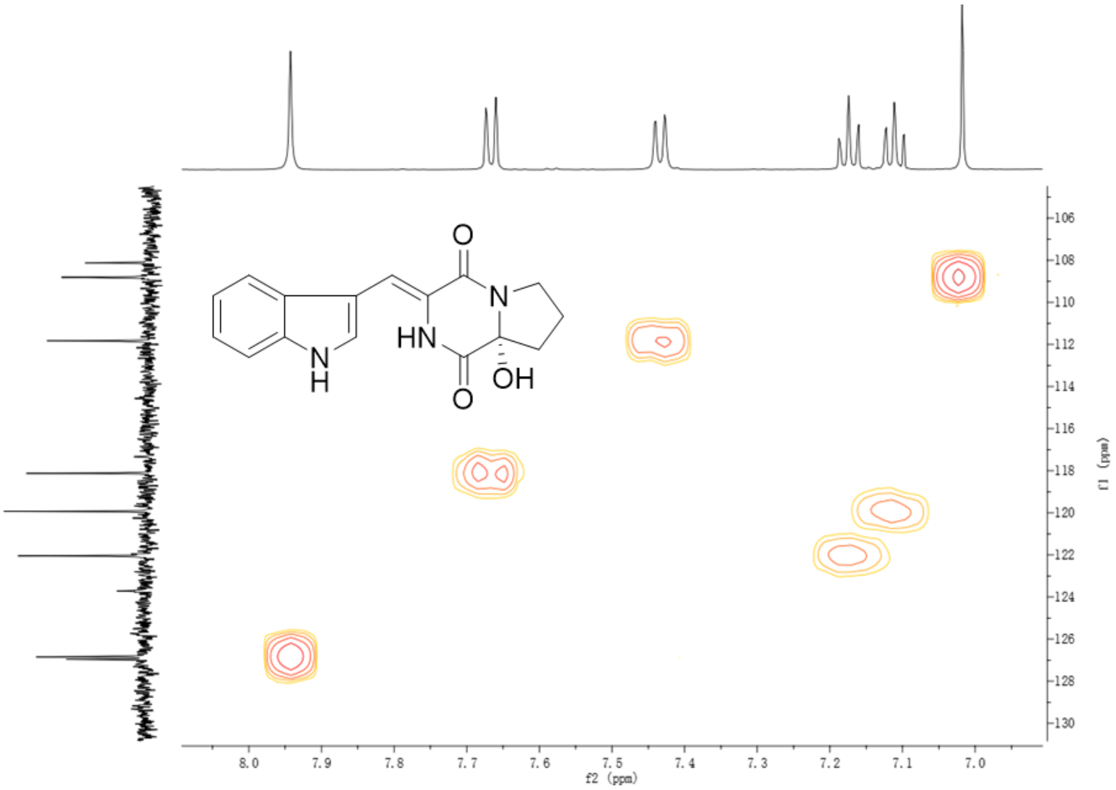

Figure S11. HMQC spectrum of asperindopiperazine A (**1**)

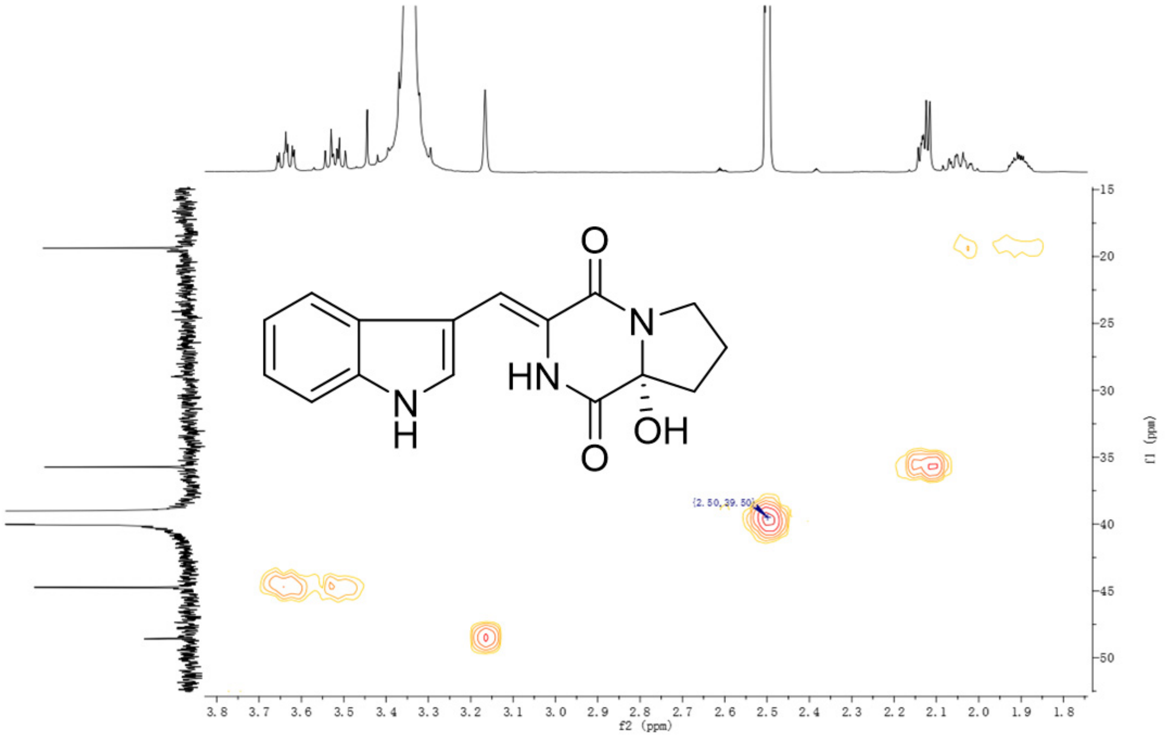

Figure S12. HMBC spectrum of asperindopiperazine A (**1**)

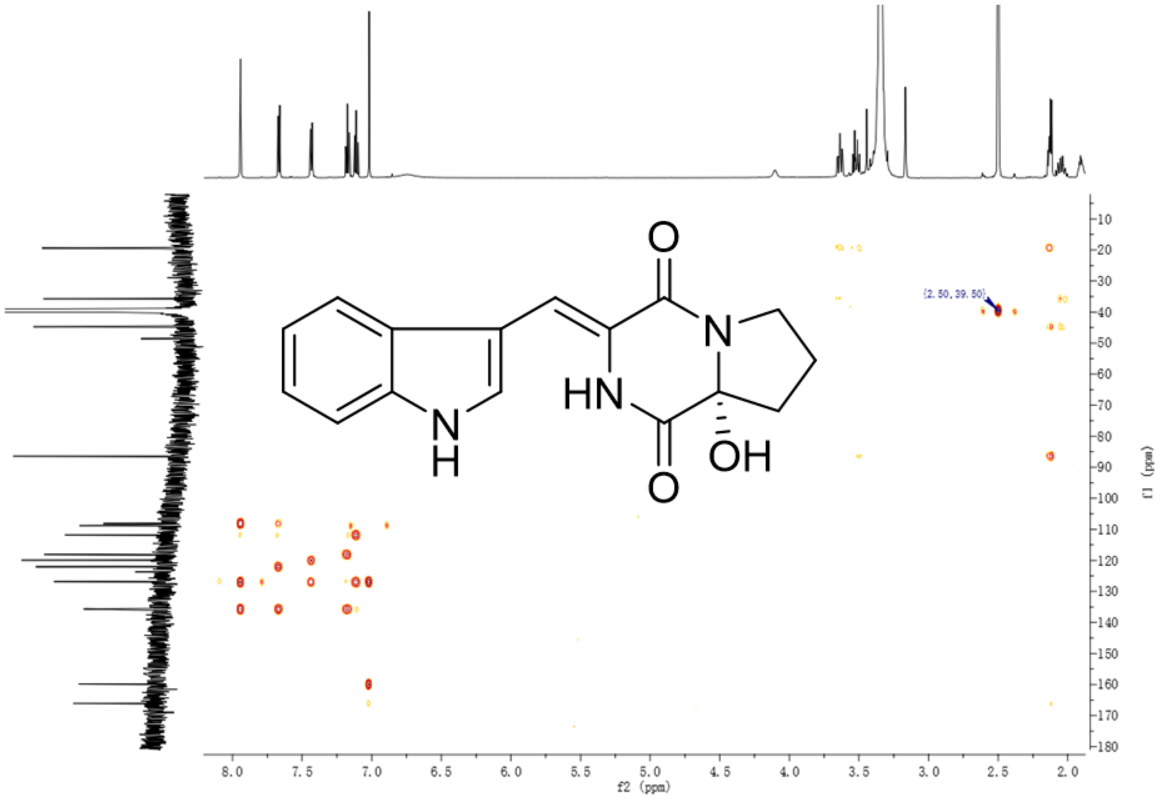

Figure S13. HMBC spectrum of asperindopiperazine A (**1**)

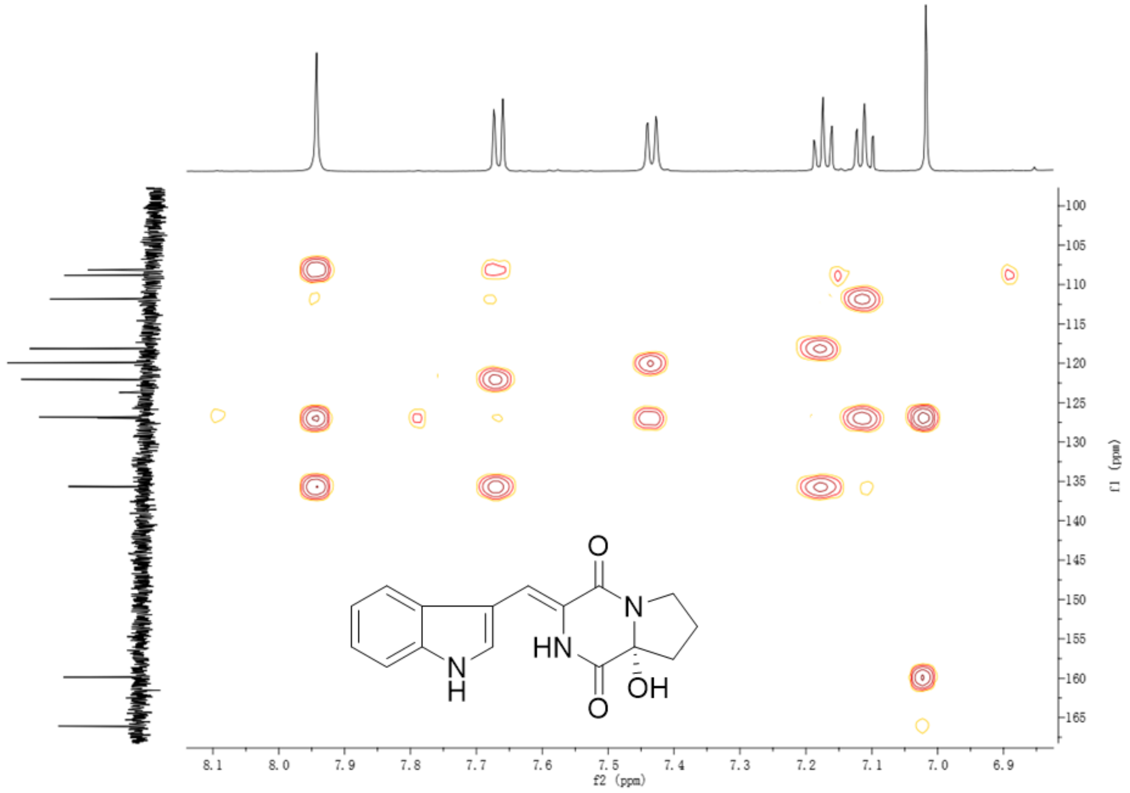

Figure S14. HMBC spectrum of asperindopiperazine A (**1**)

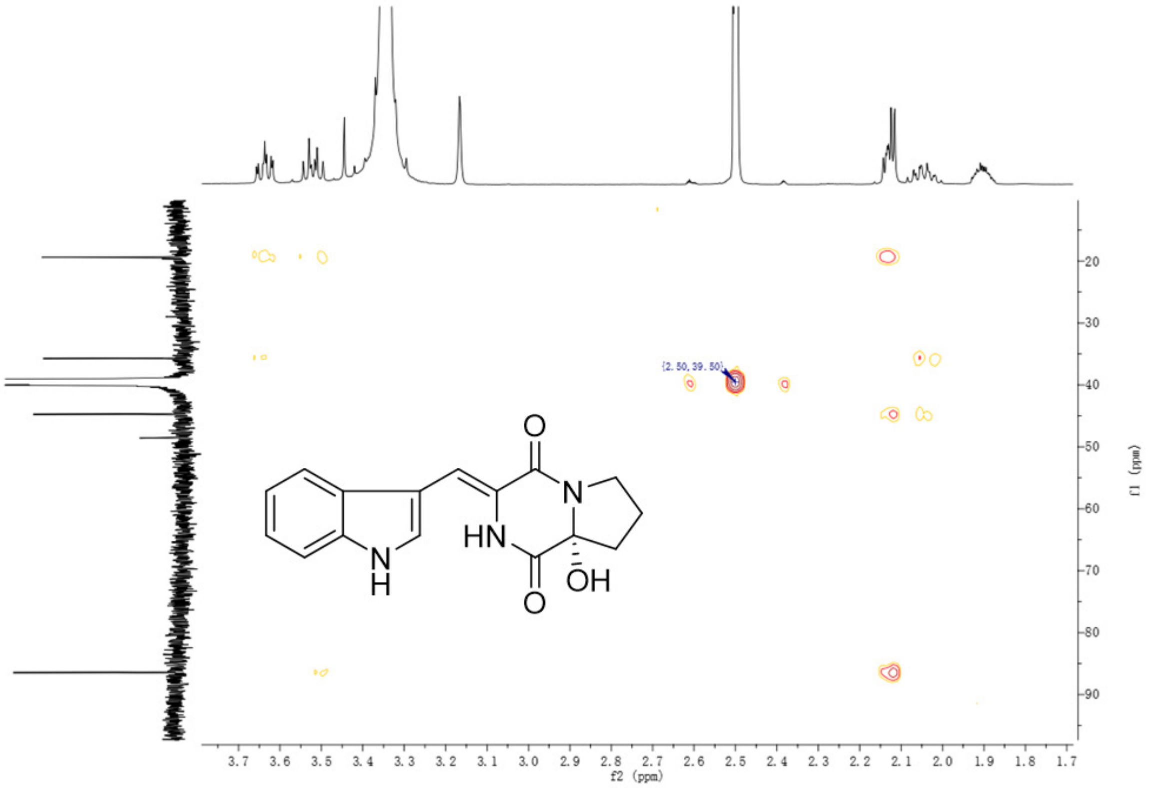

Figure S15. HRESIMS spectrum of asperindopiperazine A (**1**)

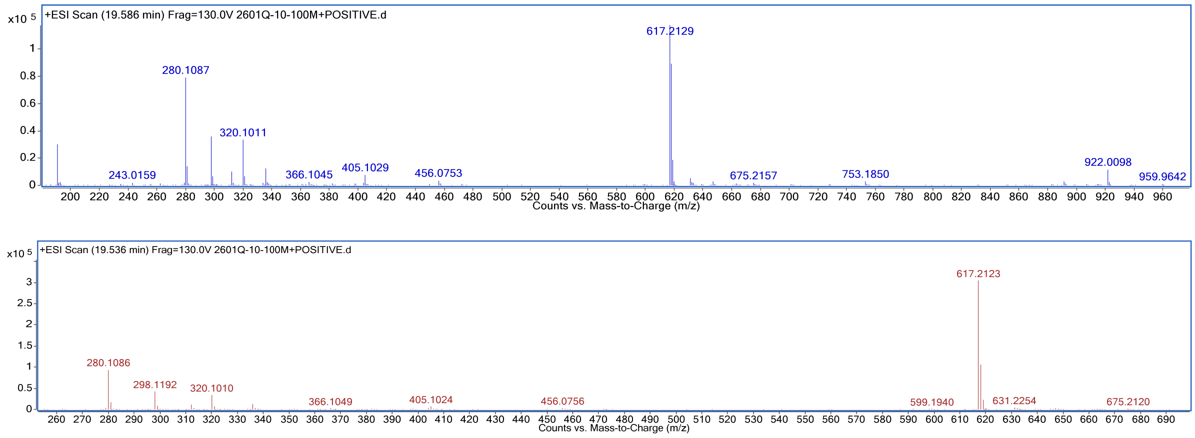

$[M+H]^+$: 298.1192 (calcd. $C_{16}H_{16}N_3O_3$, 298.1192), $[M+Na]^+$: 320.1011 (calcd. $C_{16}H_{15}N_3NaO_3$, 320.1011), and $[2M+Na]^+$: 617.2123 ((calcd. $C_{32}H_{30}N_6NaO_6$, 617.2125).

Figure S16. IR spectrum of asperindopiperazine A (**1**)

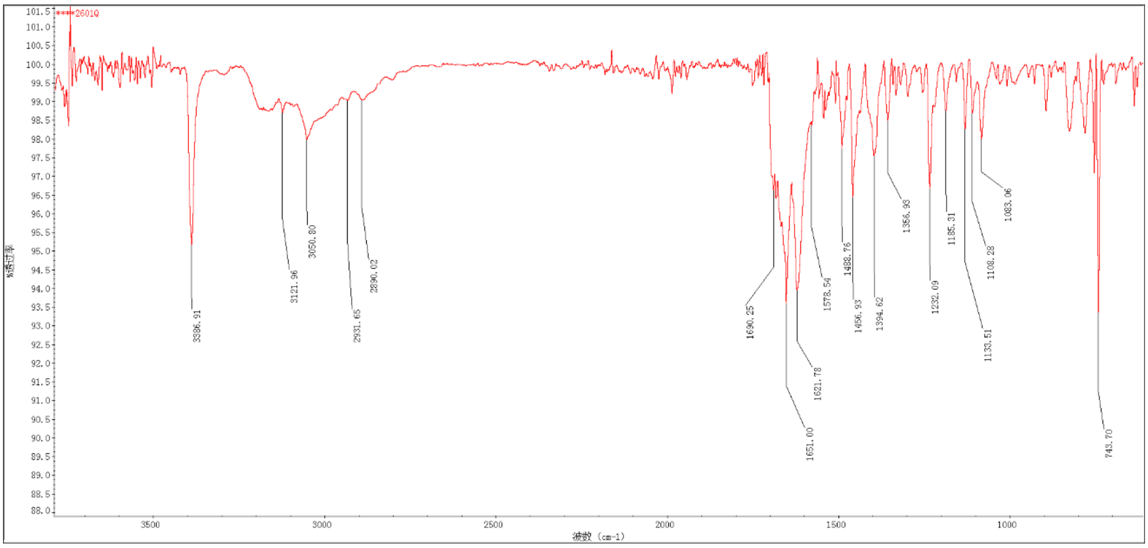

Figure S17. UV spectrum of asperindopiperazine A (**1**)

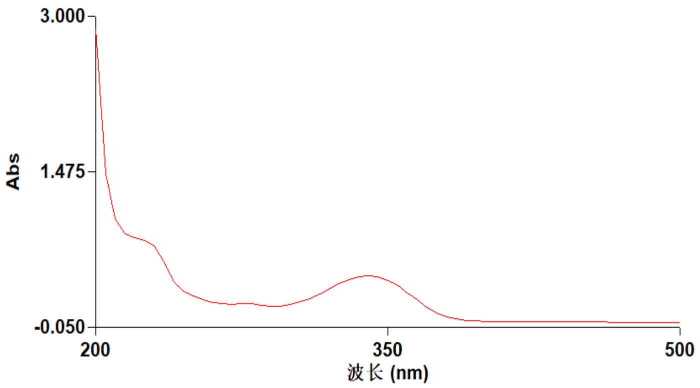

Figure S18. ^{1}H NMR spectrum of asperindopiperazine B (**2**)

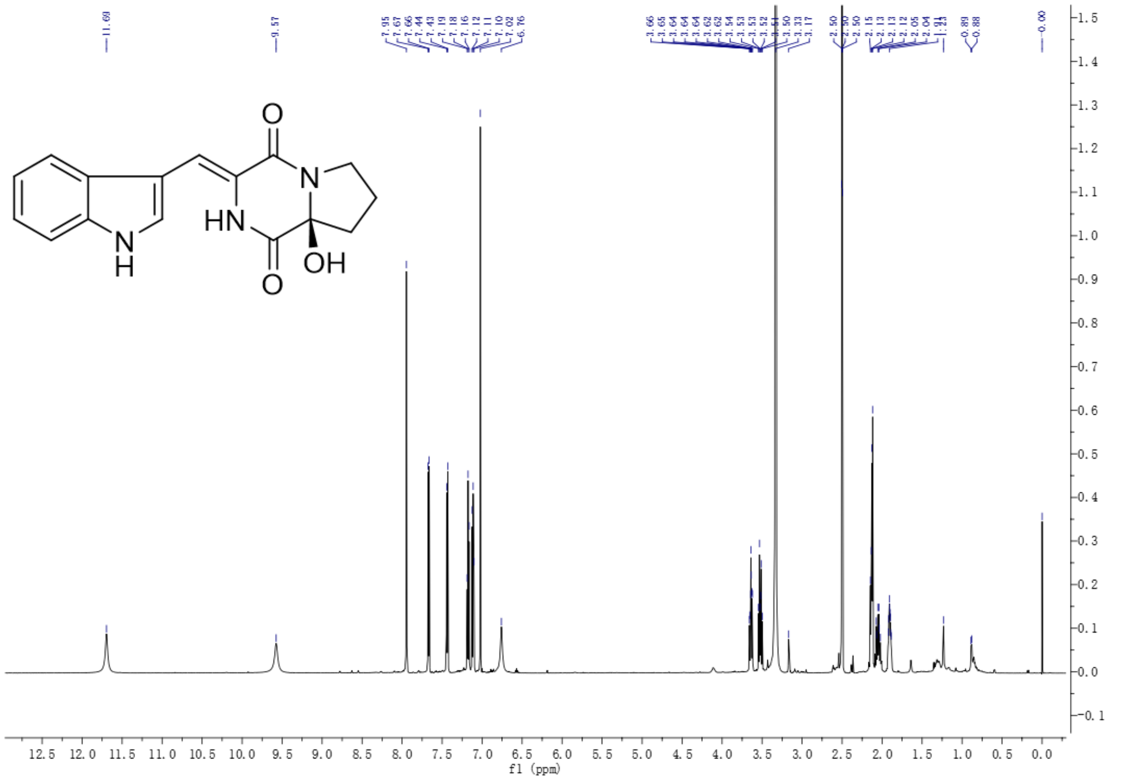

Figure S19. ^{1}H NMR spectrum of asperindopiperazine B (**2**)

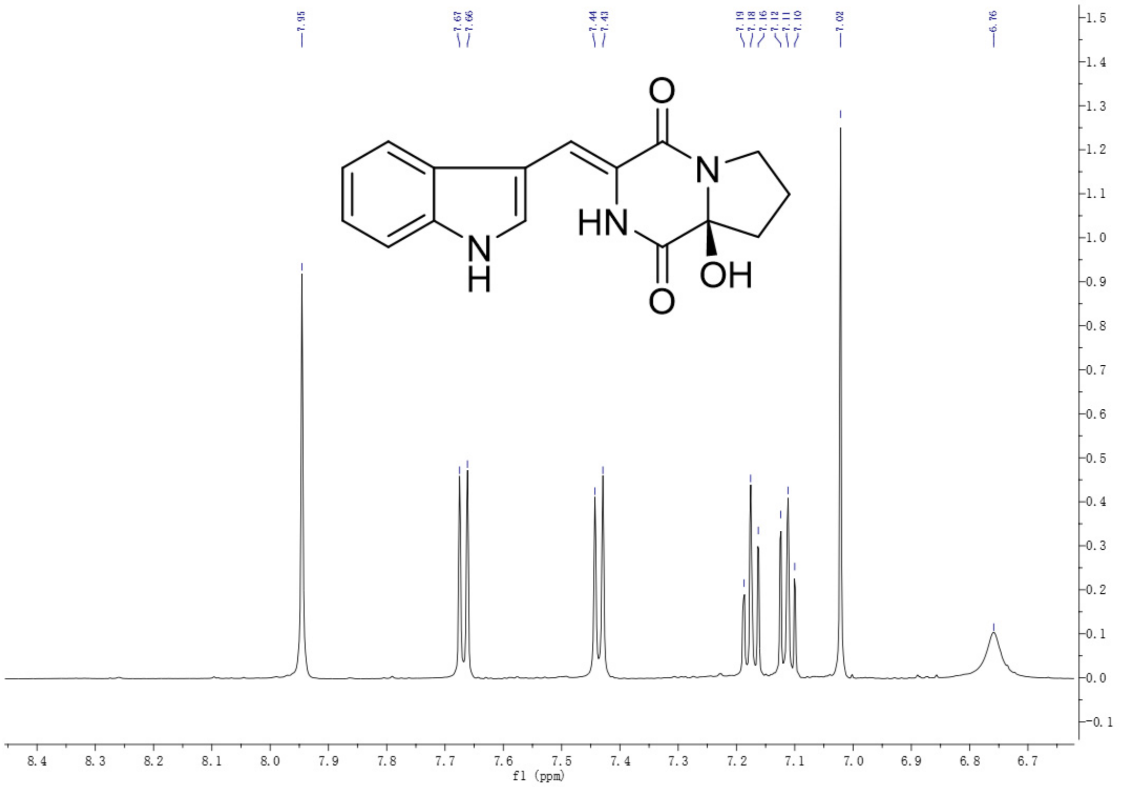

Figure S20. ^{1}H NMR spectrum of asperindopiperazine B (**2**)

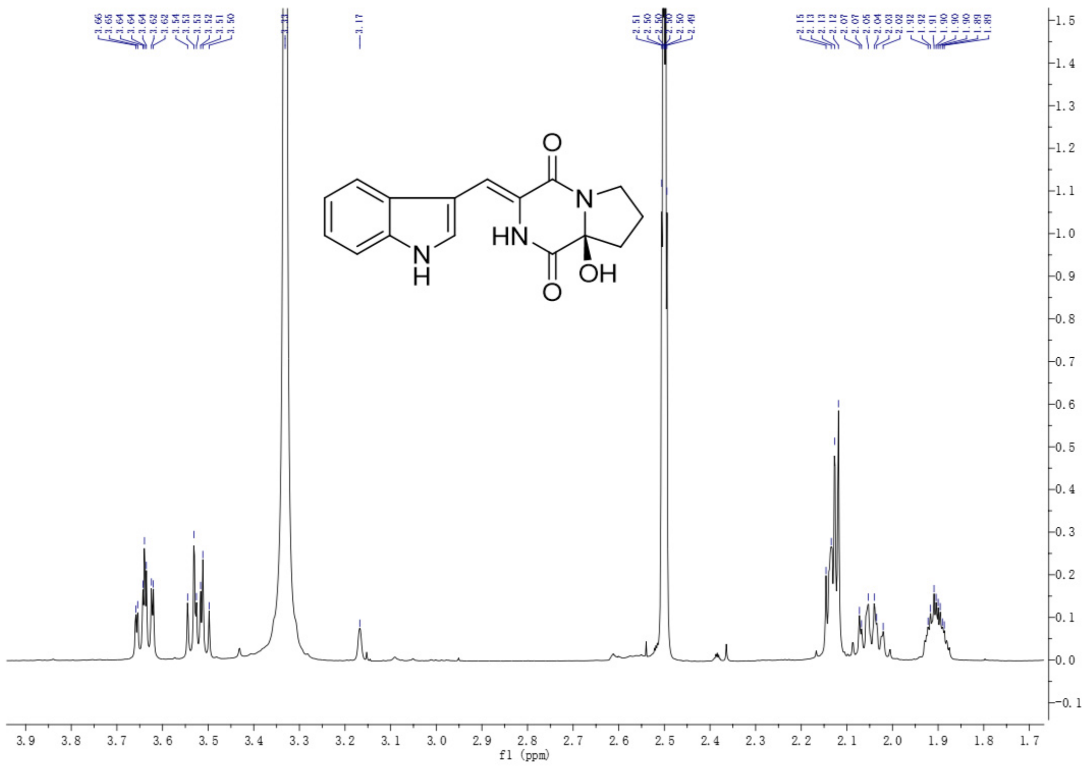

Figure S21. ^{13}C NMR spectrum of asperindopiperazine B (**2**)

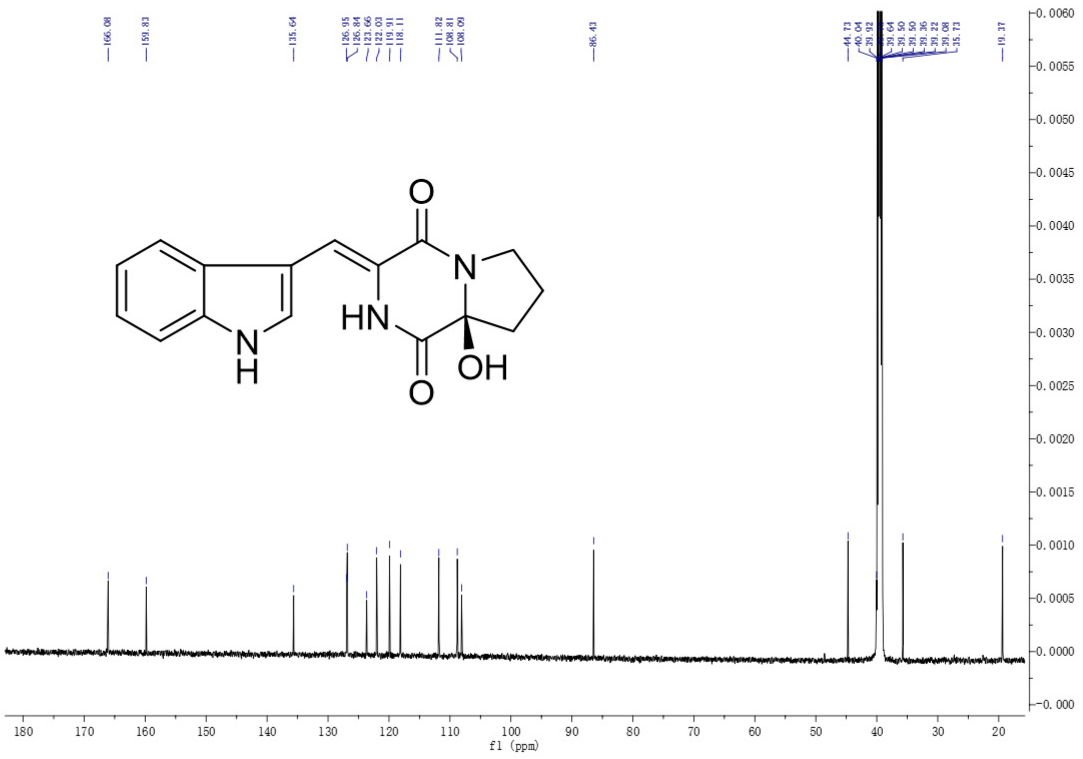

Figure S22. ^{13}C NMR spectrum of asperindopiperazine B (**2**)

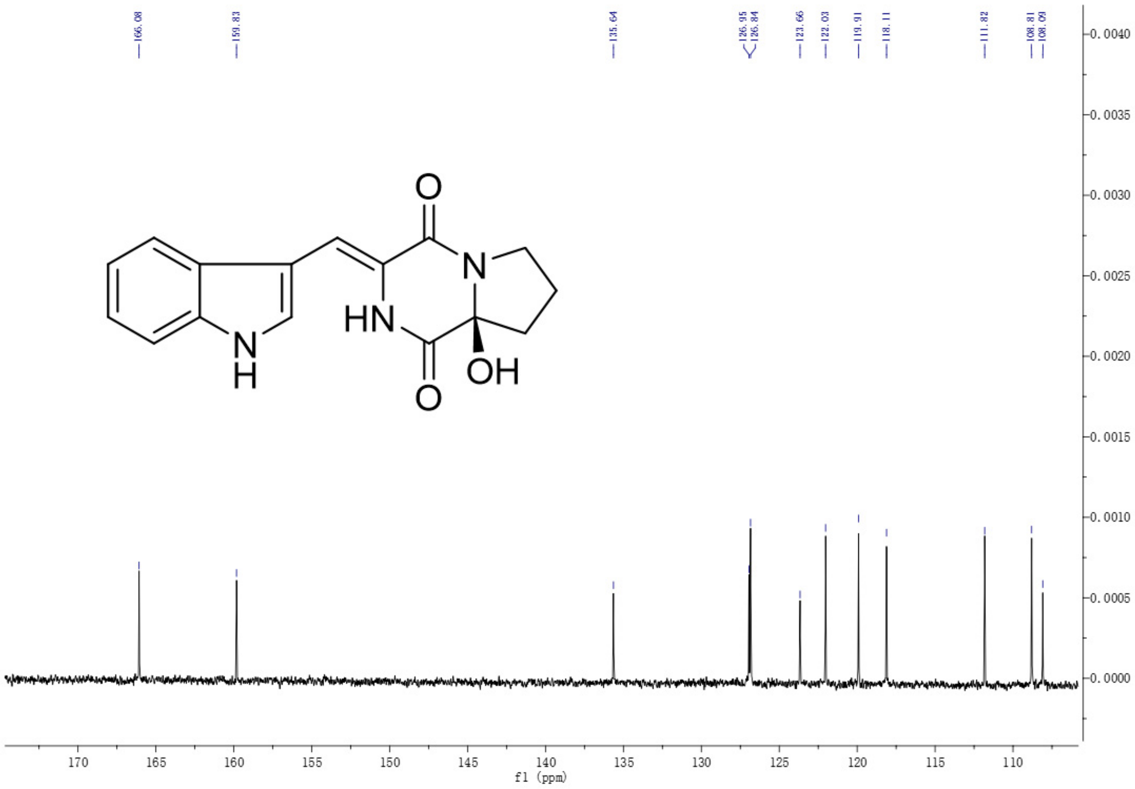

Figure S23. ^{13}C NMR spectrum of asperindopiperazine B (**2**)

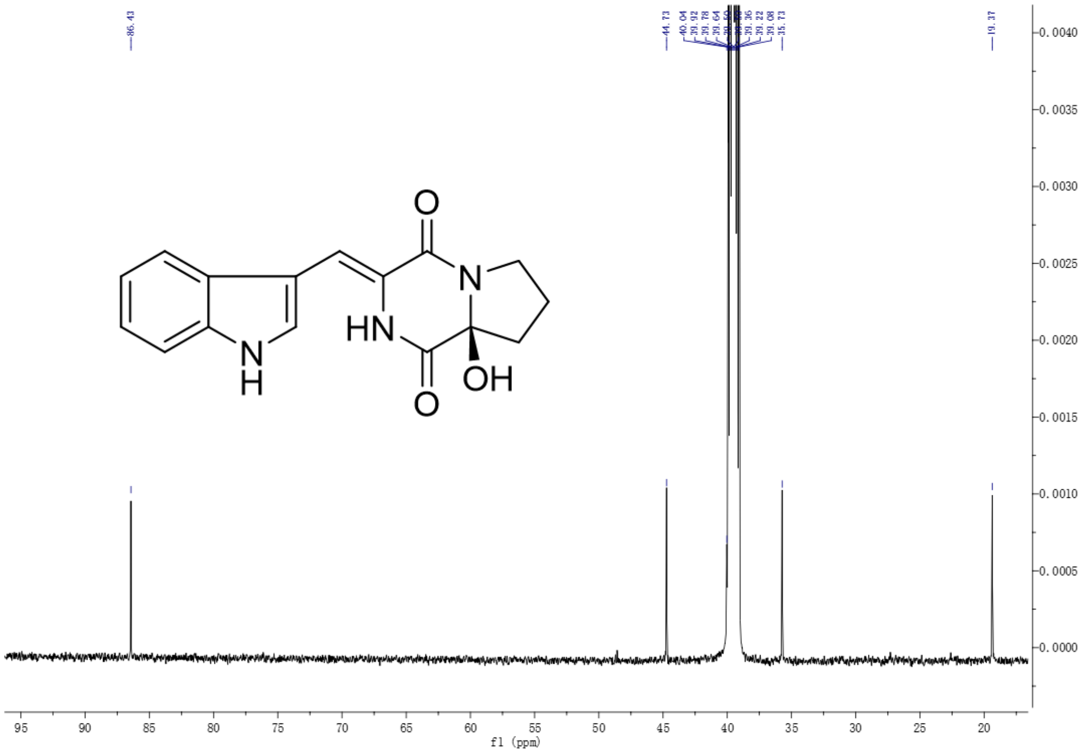

Figure S24. HRESIMS spectrum of asperindopiperazine B (**2**)

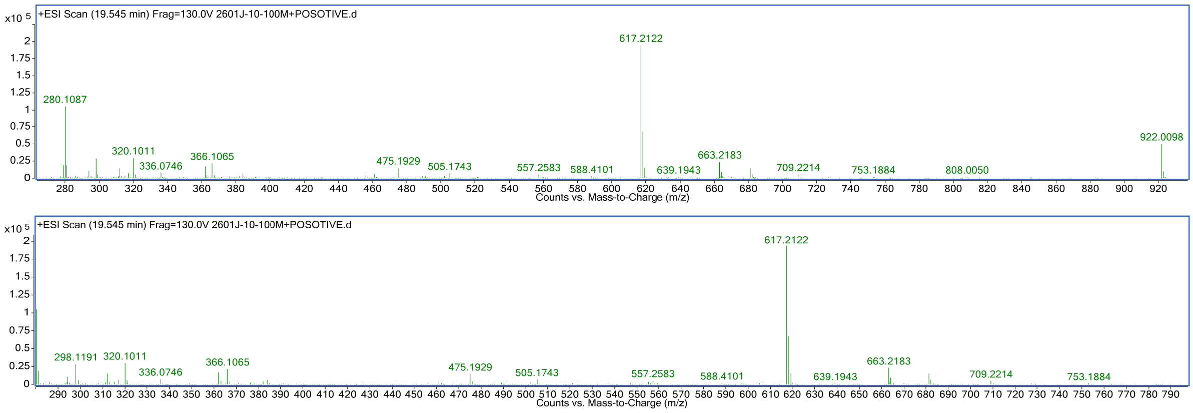

$[M+H]^+$: 298.1191 (calcd. $C_{16}H_{16}N_3O_3$, 298.1192), $[M+Na]^+$: 320.1011 (calcd. $C_{16}H_{15}N_3NaO_3$, 320.1011), and $[2M+Na]^+$: 617.2122 ((calcd. $C_{32}H_{30}N_6NaO_6$, 617.2125).

Figure S25. IR spectrum of asperindopiperazine B (**2**)

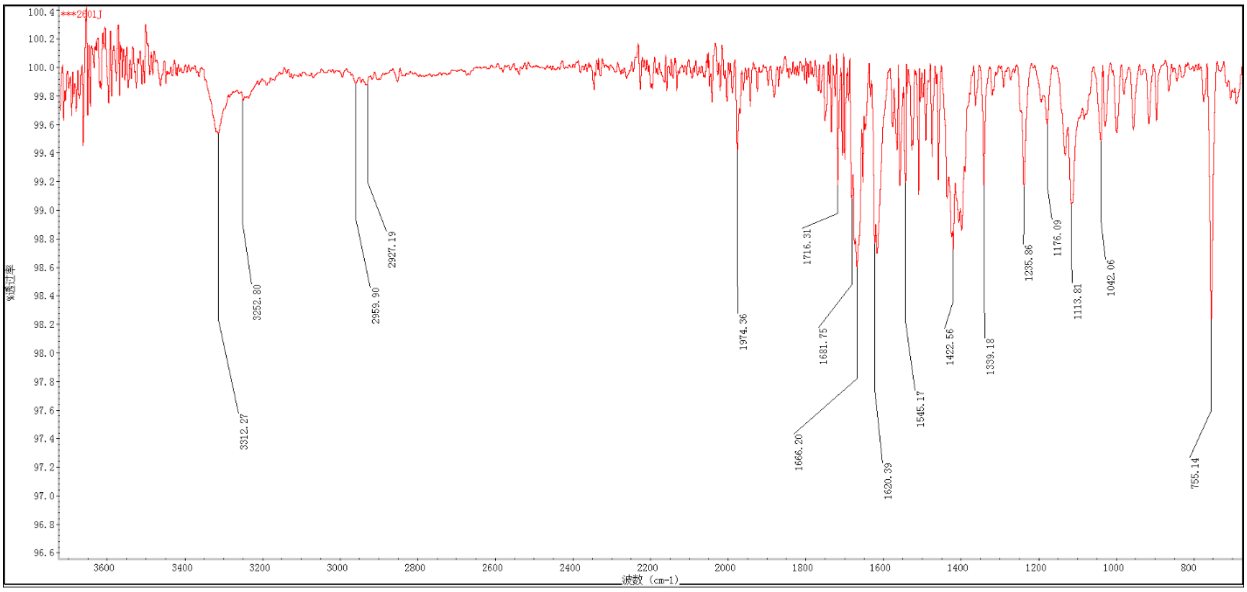

Figure S26. UV spectrum of asperindopiperazine B (**2**)

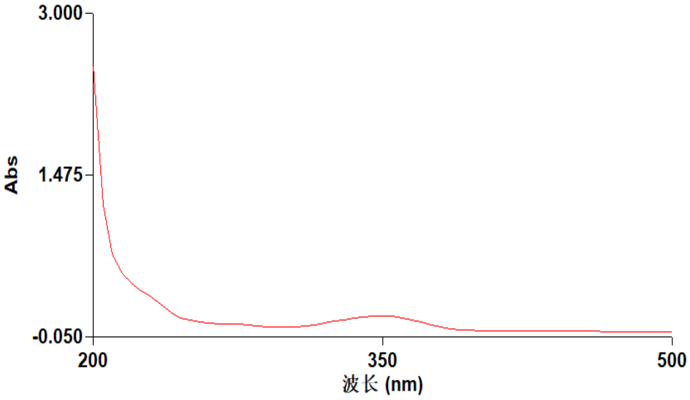

Figure S27. ^{1}H NMR spectrum of asperindopiperazine C (**3**)

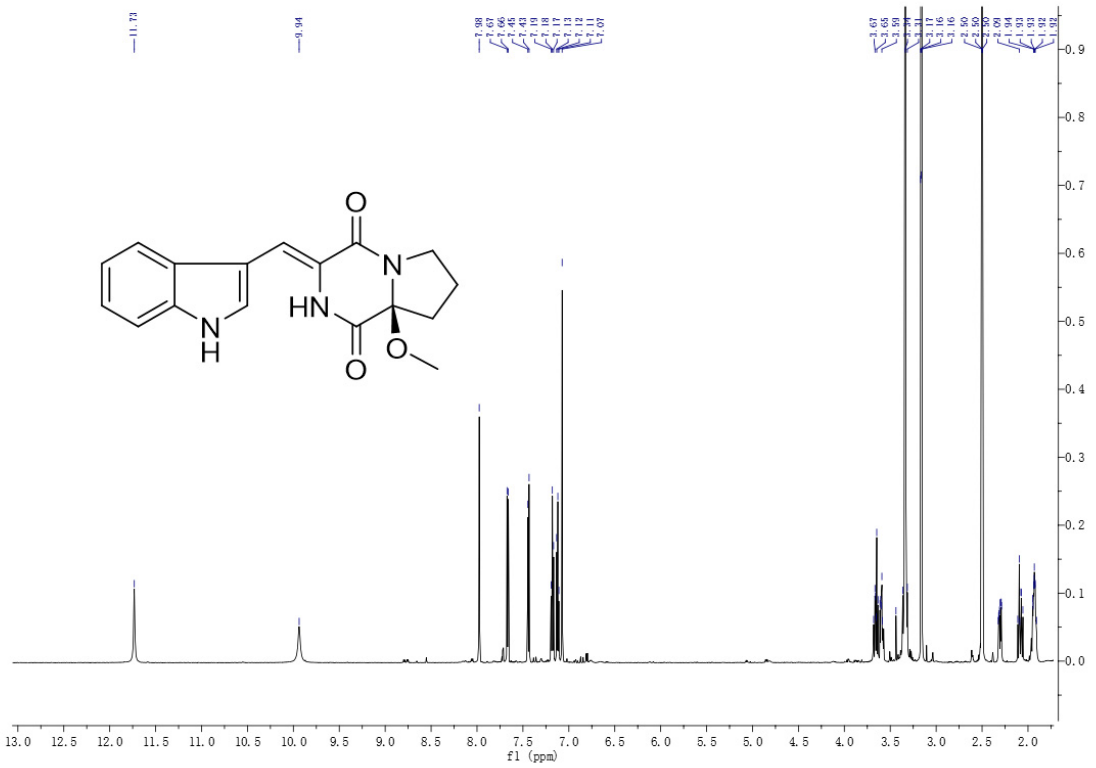

Figure S28. ^{1}H NMR spectrum of asperindopiperazine C (**3**)

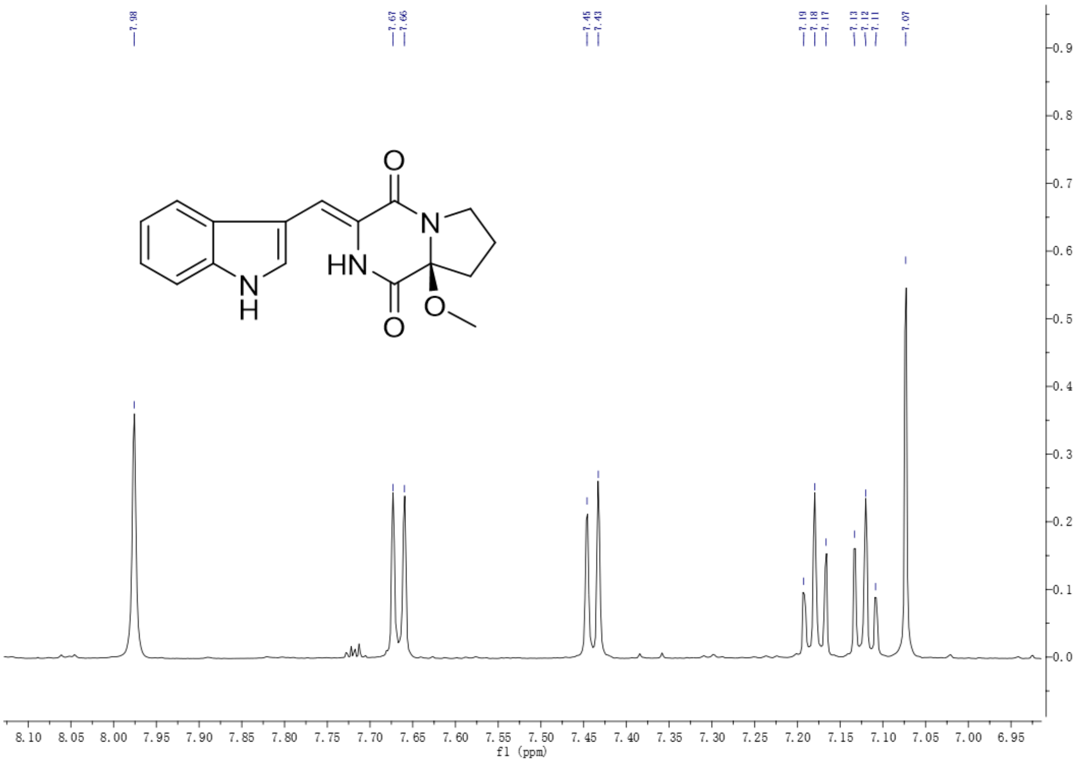

Figure S29. ^{1}H NMR spectrum of asperindopiperazine C (**3**)

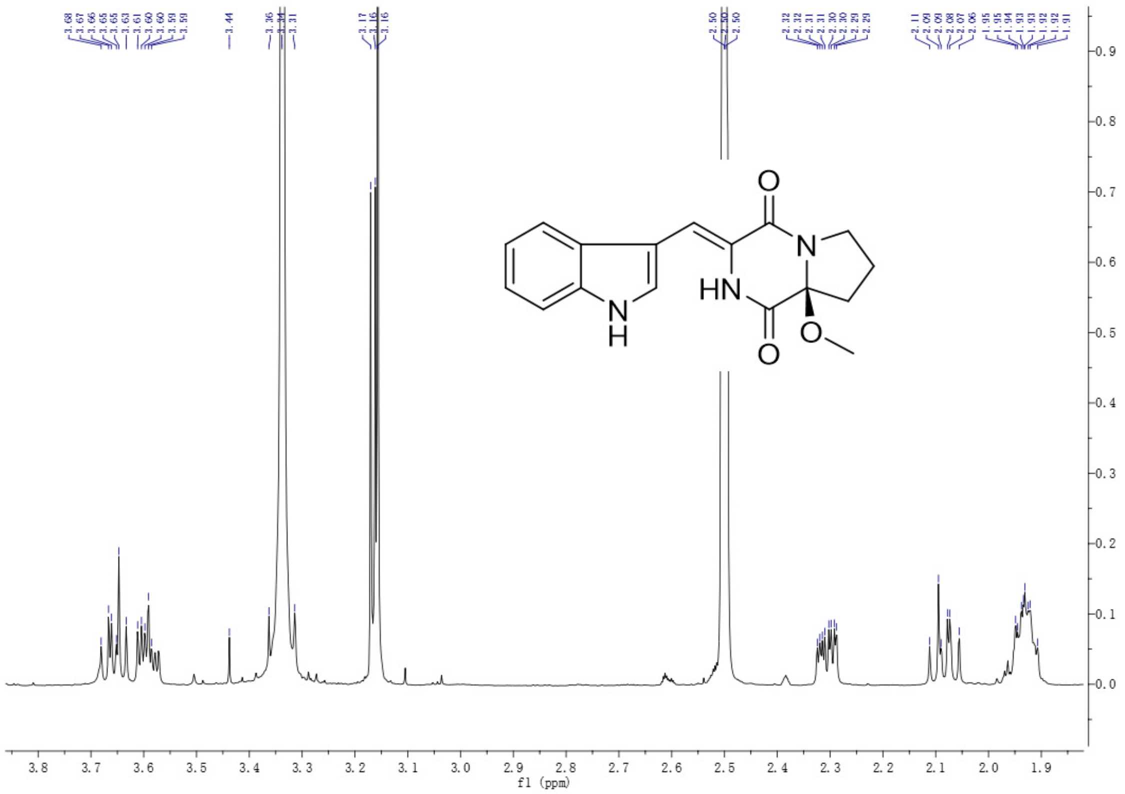

Figure S30. ^{13}C NMR spectrum of asperindopiperazine C (**3**)

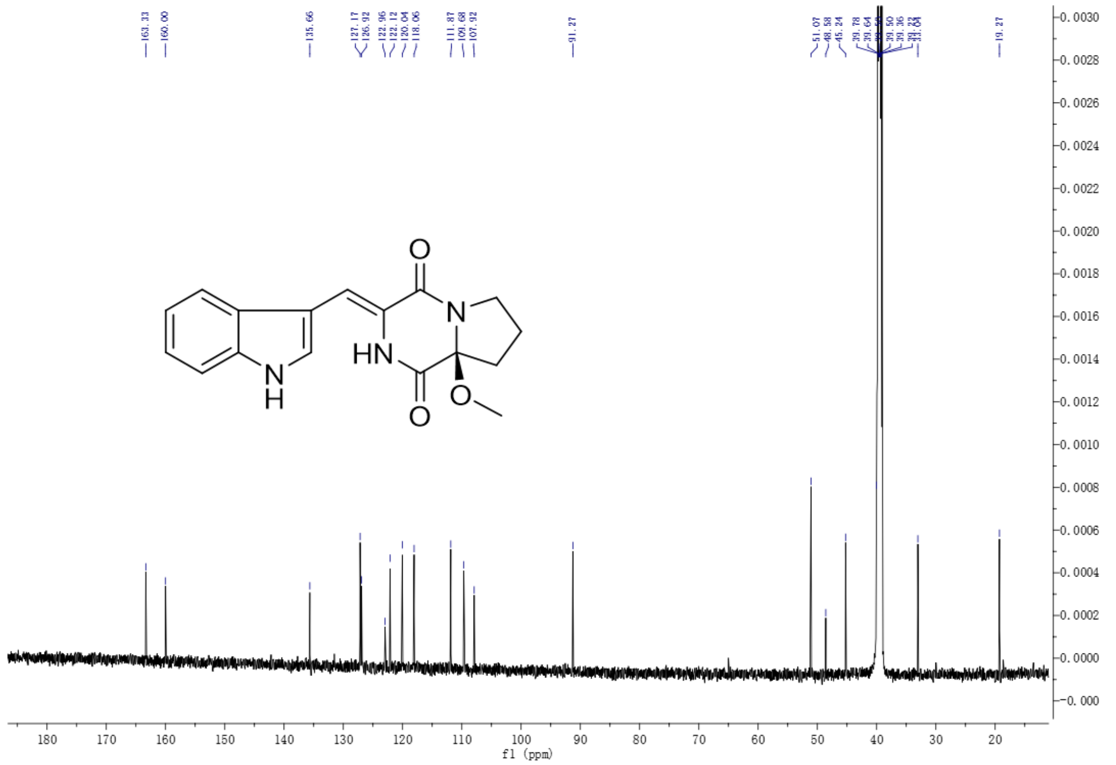

Figure S31. ^{13}C NMR spectrum of asperindopiperazine C (**3**)

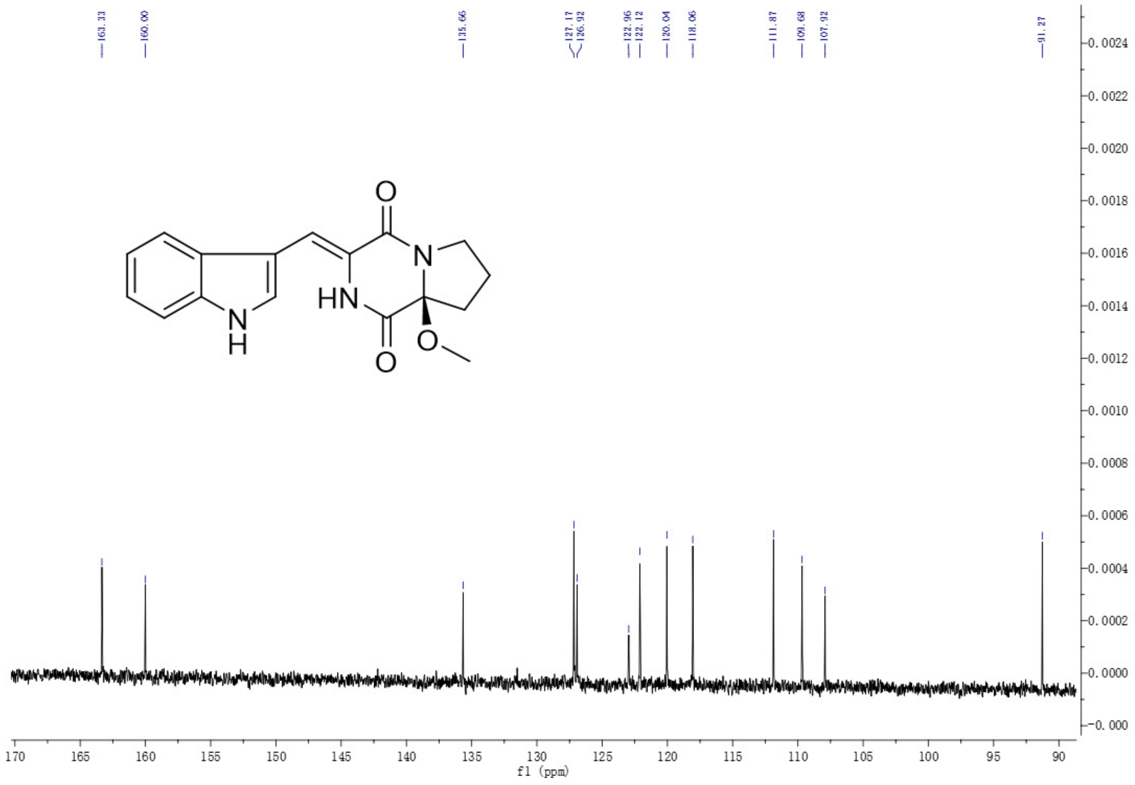

Figure S32. ^{13}C NMR spectrum of asperindopiperazine C (**3**)

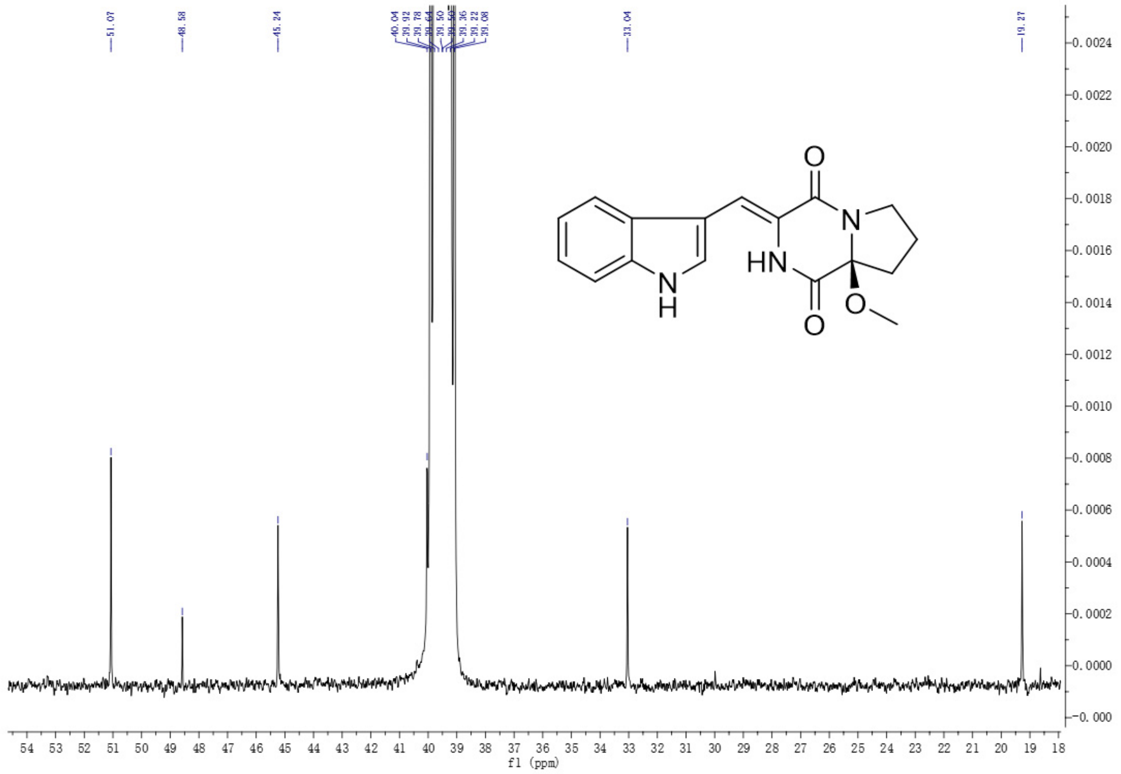

Figure S33. HMQC spectrum of asperindopiperazine C (**3**)

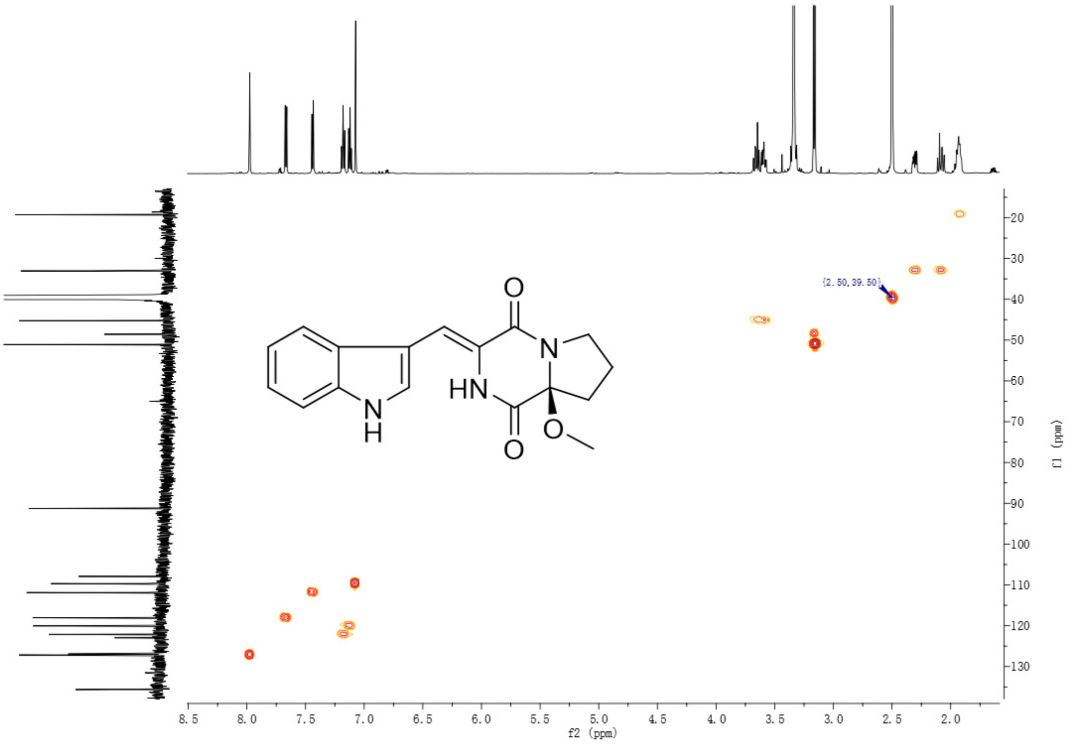

Figure S34. HMQC spectrum of asperindopiperazine C (**3**)

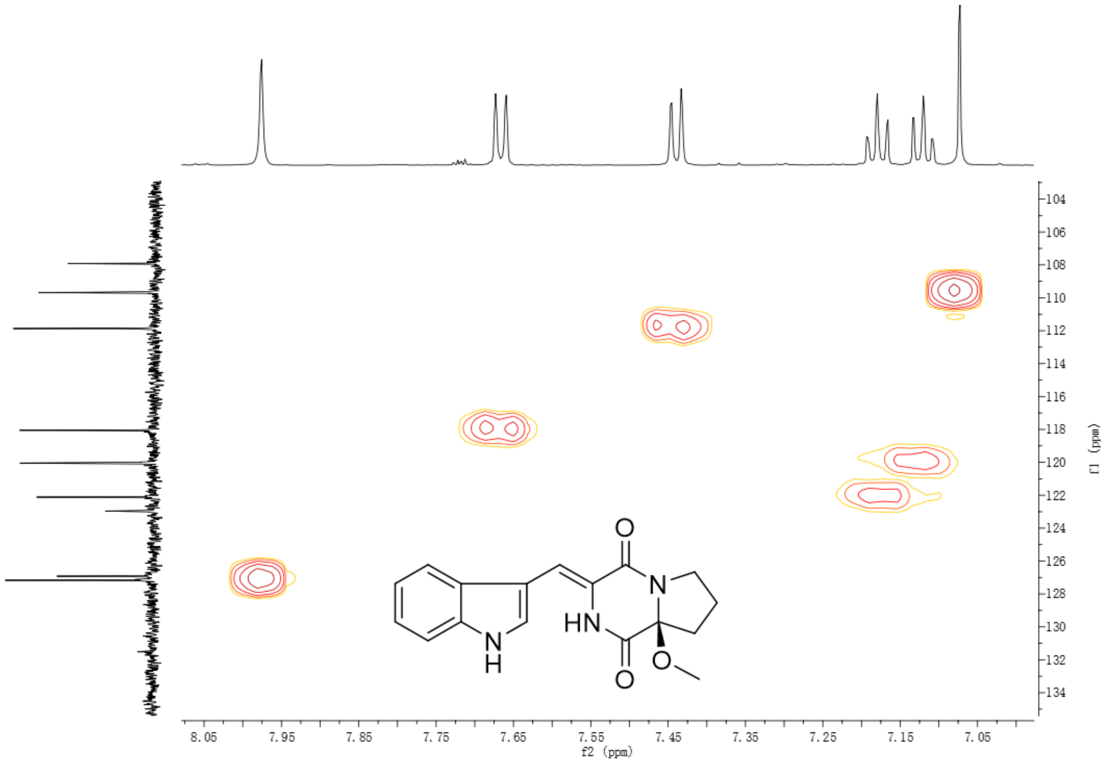

Figure S35. HMQC spectrum of asperindopiperazine C (**3**)

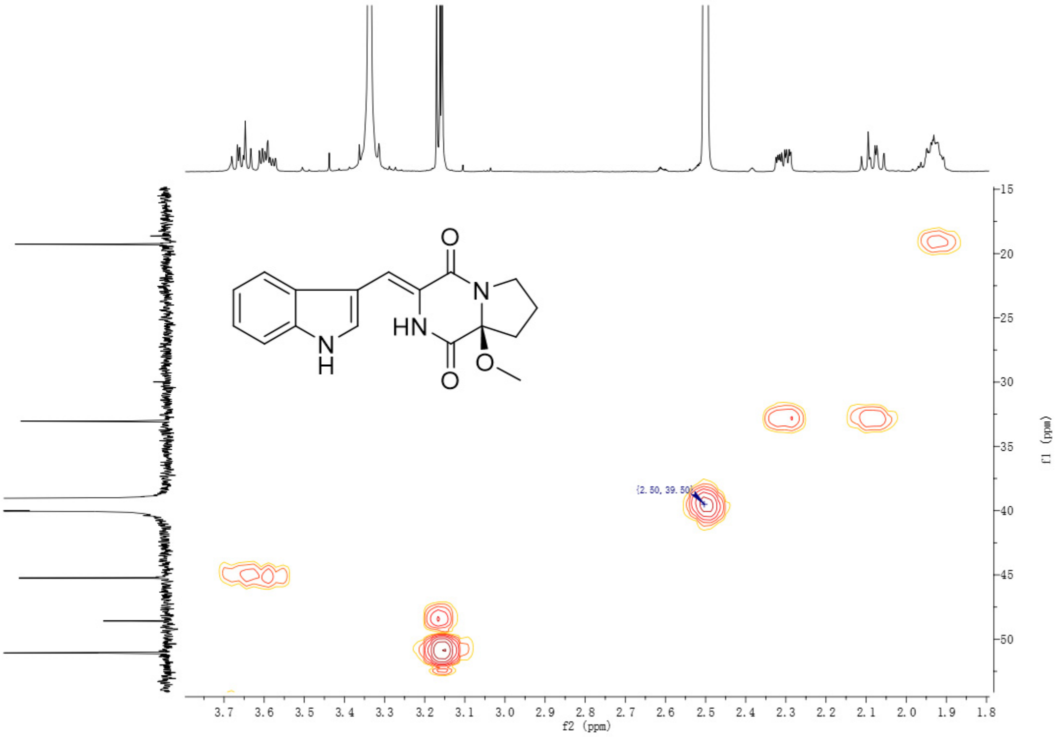

Figure S36. HMBC spectrum of asperindopiperazine C (**3**)

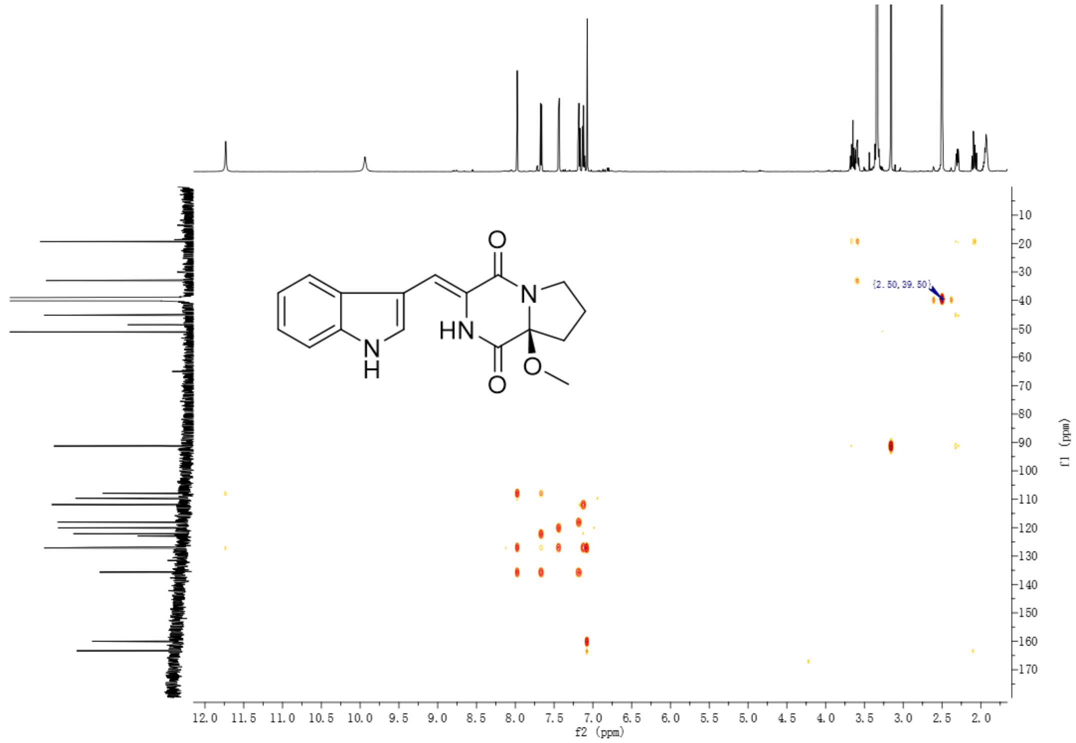

Figure S37. HMBC spectrum of asperindopiperazine C (**3**)

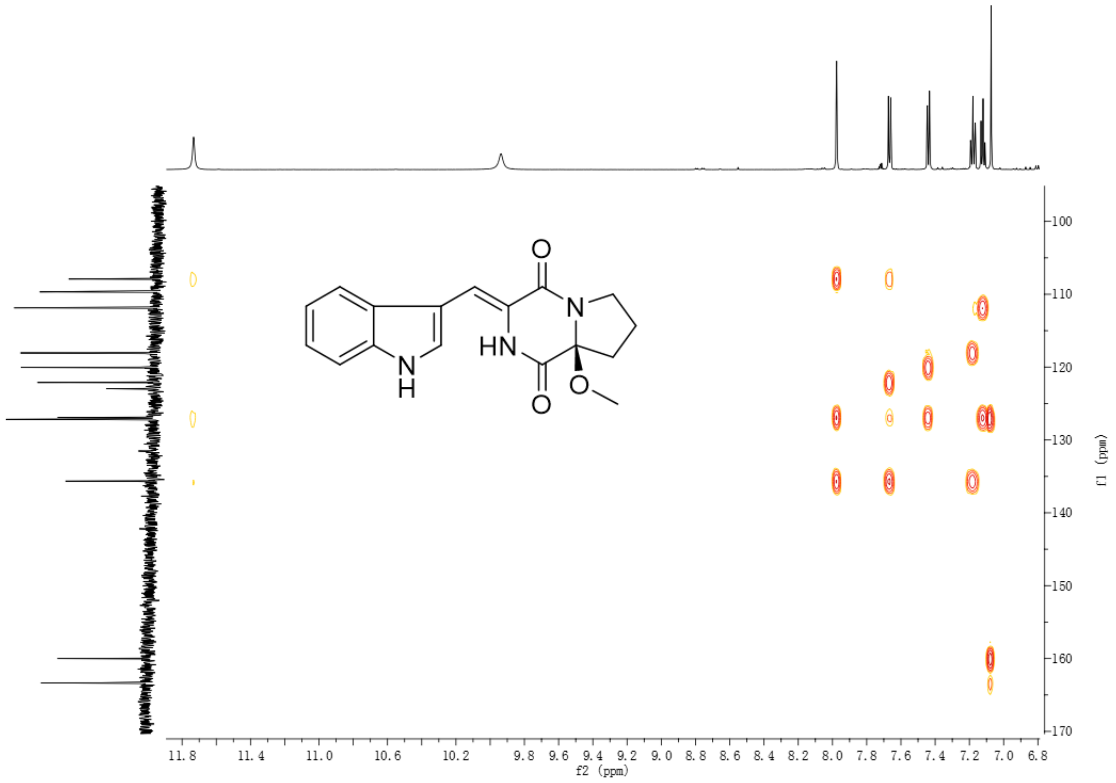

Figure S38. HMBC spectrum of asperindopiperazine C (**3**)

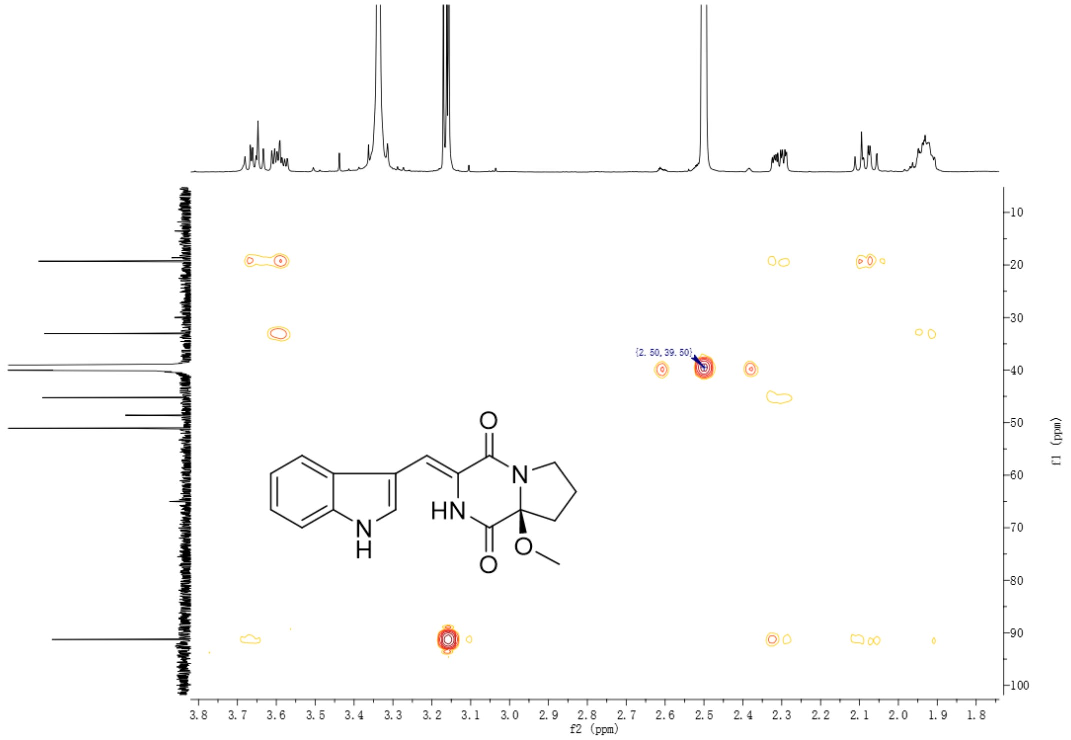

Figure S39. HRESIMS spectrum of asperindopiperazine C (**3**)

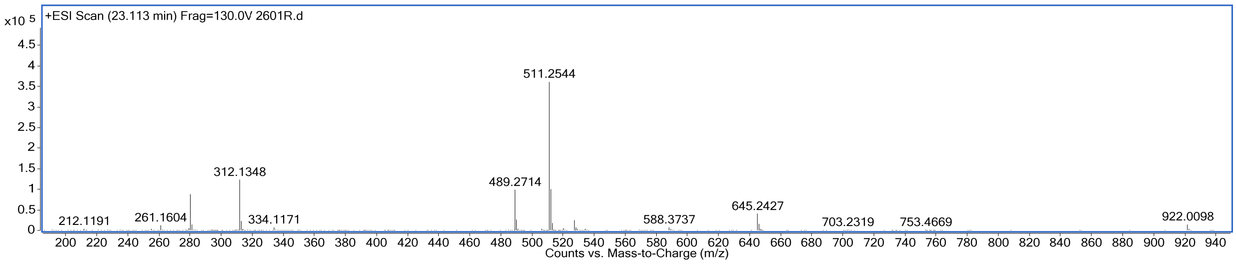

[M+H]$^+$: 312.1348 (calcd. $C_{17}H_{18}N_3O_3$, 312.1348), [M+Na]$^+$: 334.1171 (calcd. $C_{17}H_{17}N_3NaO_3$, 334.1168), and [2M+Na]$^+$: 645.2427 (calcd. $C_{34}H_{34}N_6NaO_6$, 645.2438).

Figure S40. IR spectrum of asperindopiperazine C (**3**)

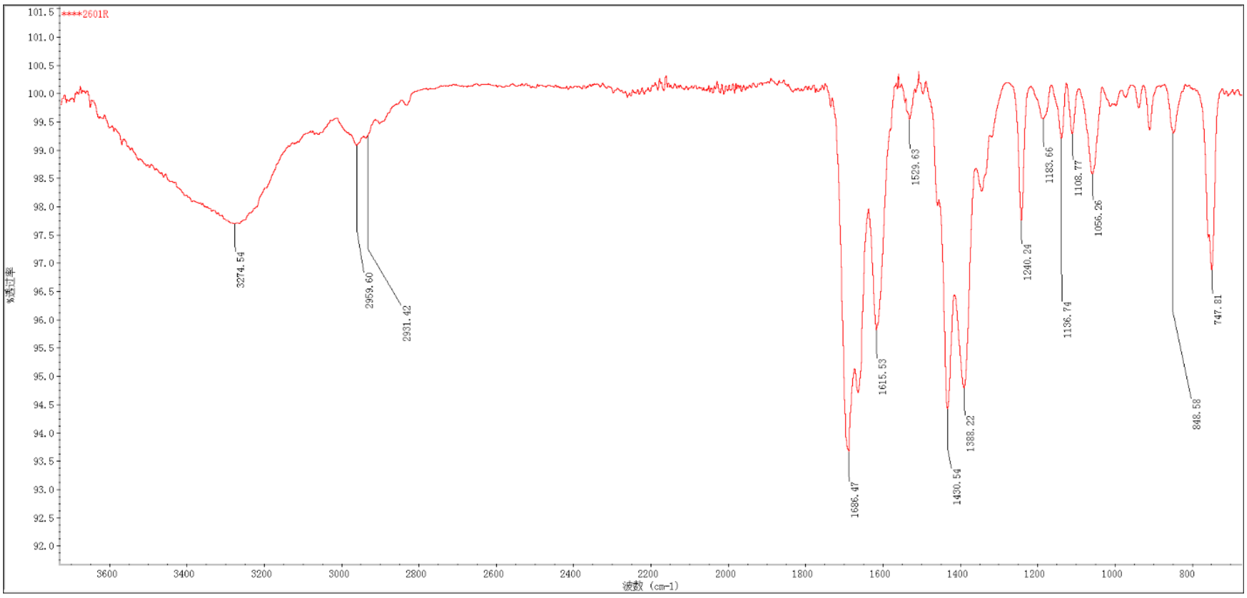

Figure S41. UV spectrum of asperindopiperazine C (**3**)

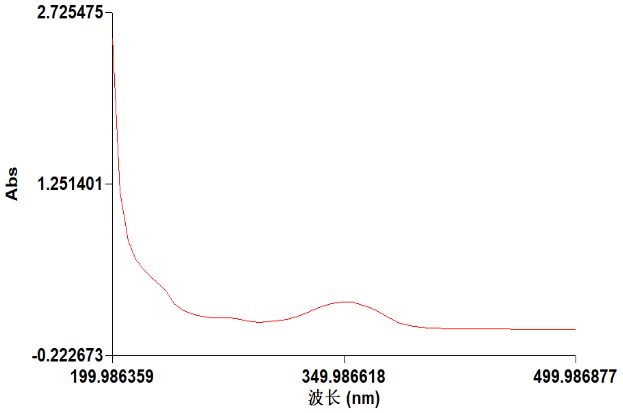

Figure S42. ^{1}H NMR spectrum of 5-methoxy-8,9-dihydroxy-8,9-deoxyaspyrone (**21**)

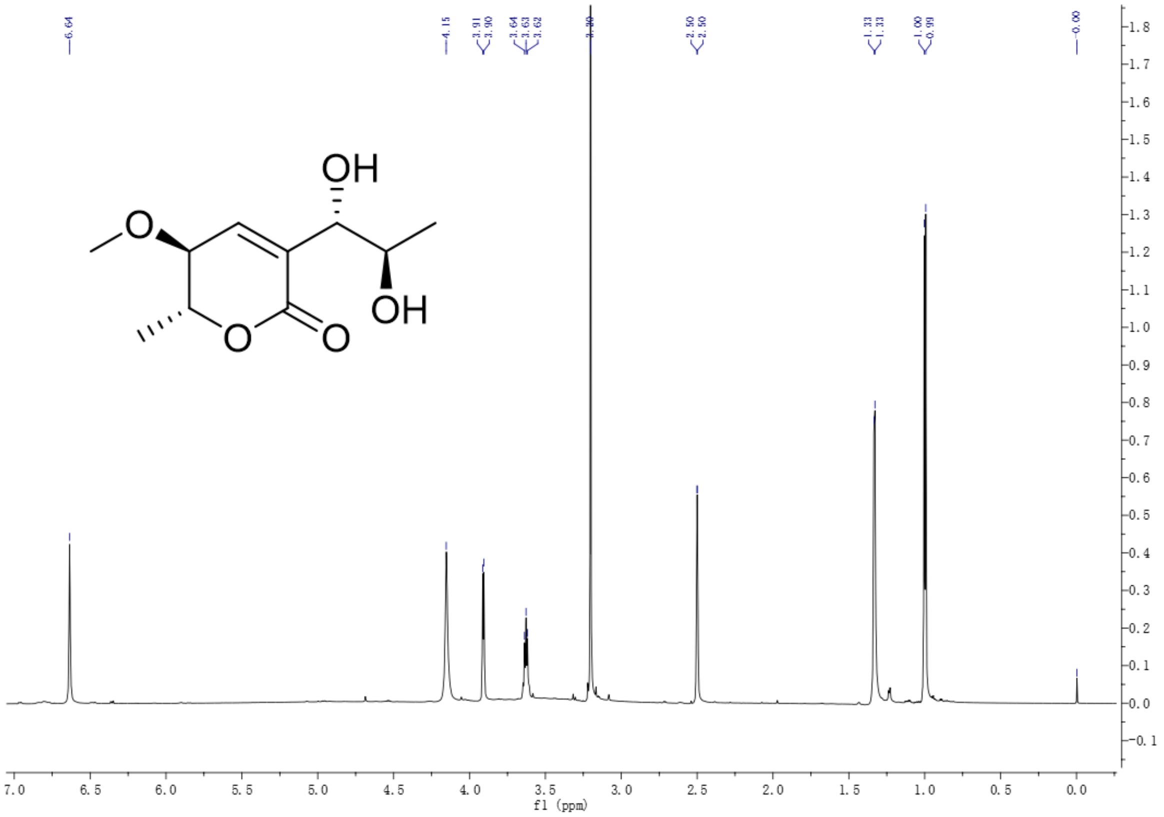

Figure S43. ^{1}H NMR spectrum of 5-methoxy-8,9-dihydroxy-8,9-deoxyaspyrone (**21**)

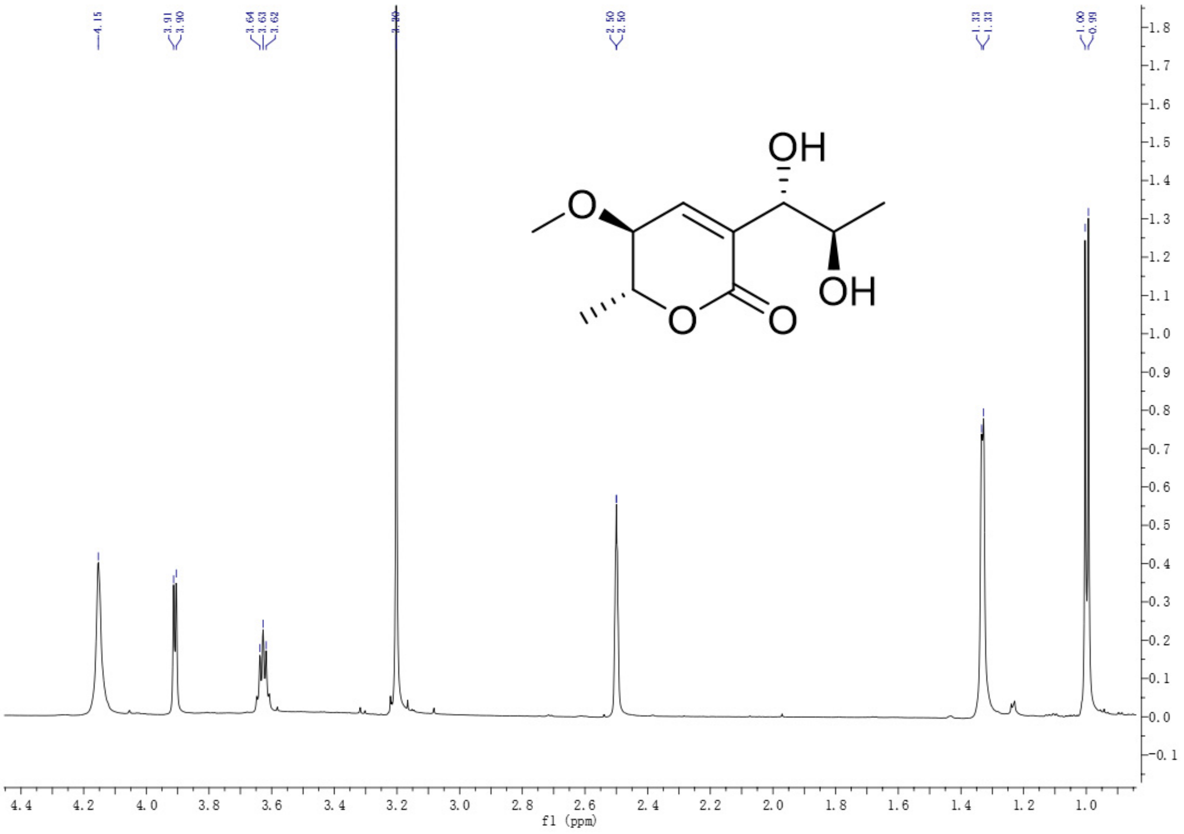

Figure S44. ^{13}C NMR spectrum of 5-methoxy-8,9-dihydroxy-8,9-deoxyaspyrone (**21**)

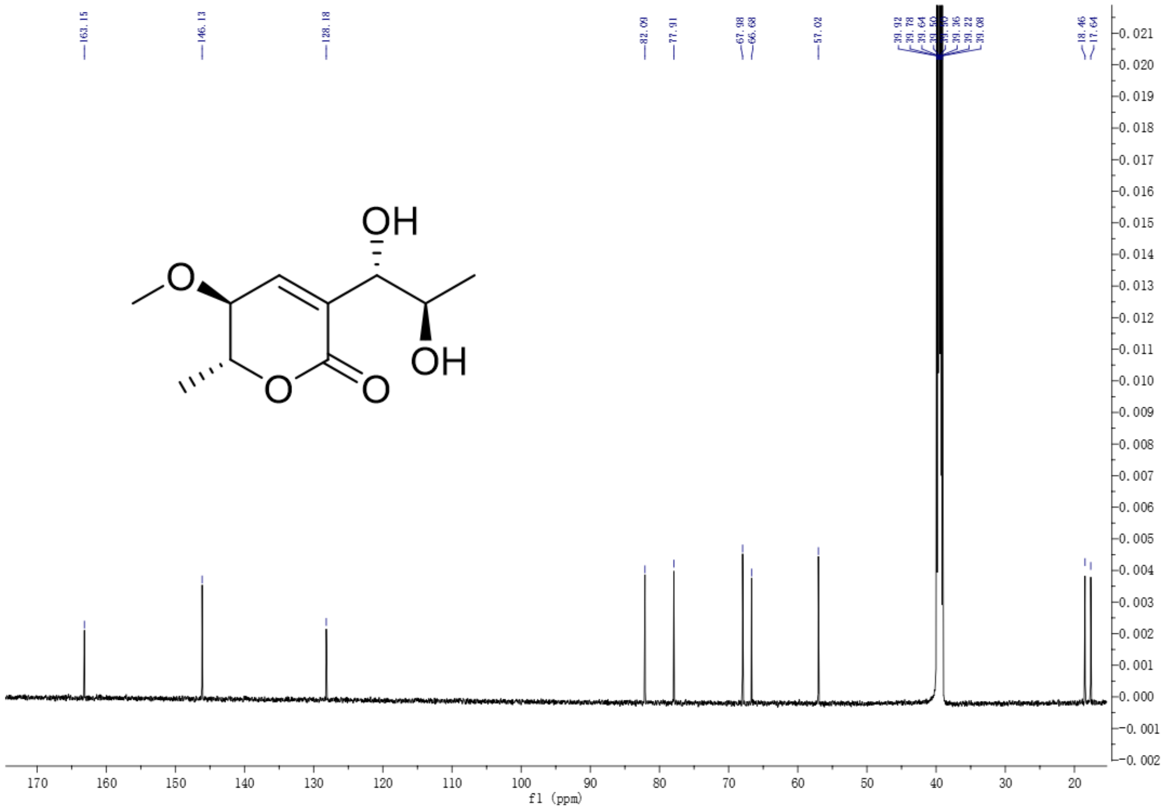

Figure S45. HMQC spectrum of 5-methoxy-8,9-dihydroxy-8,9-deoxyaspyrone (**21**)

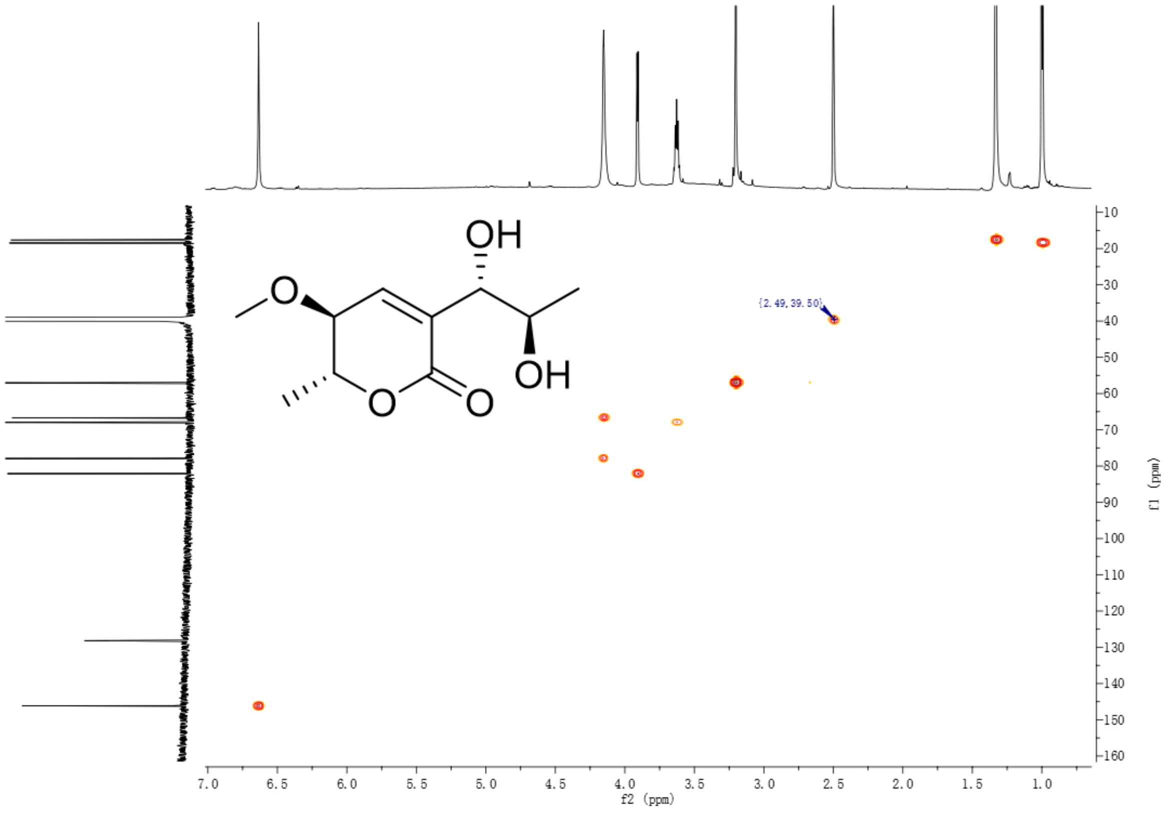

Figure S46. COSY spectrum of 5-methoxy-8,9-dihydroxy-8,9-deoxyaspyrone (**21**)

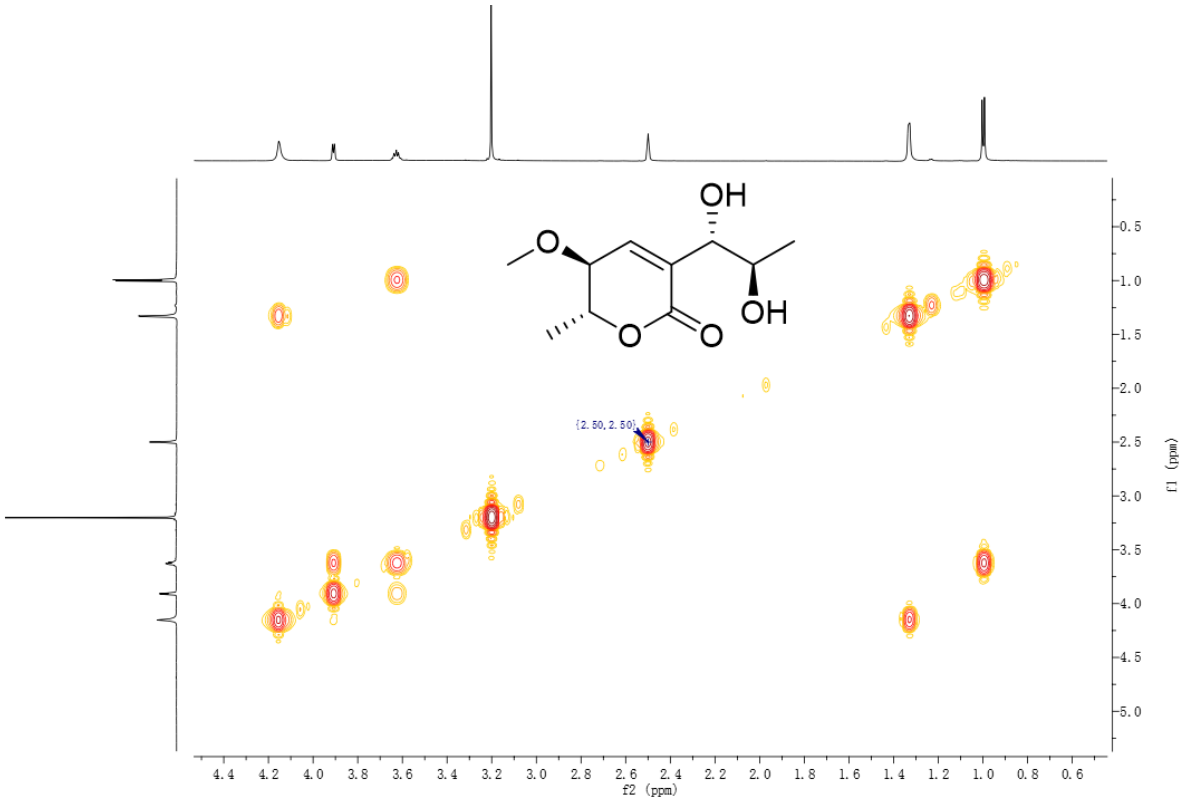

Figure S47. HMBC spectrum of 5-methoxy-8,9-dihydroxy-8,9-deoxyaspyrone (**21**)

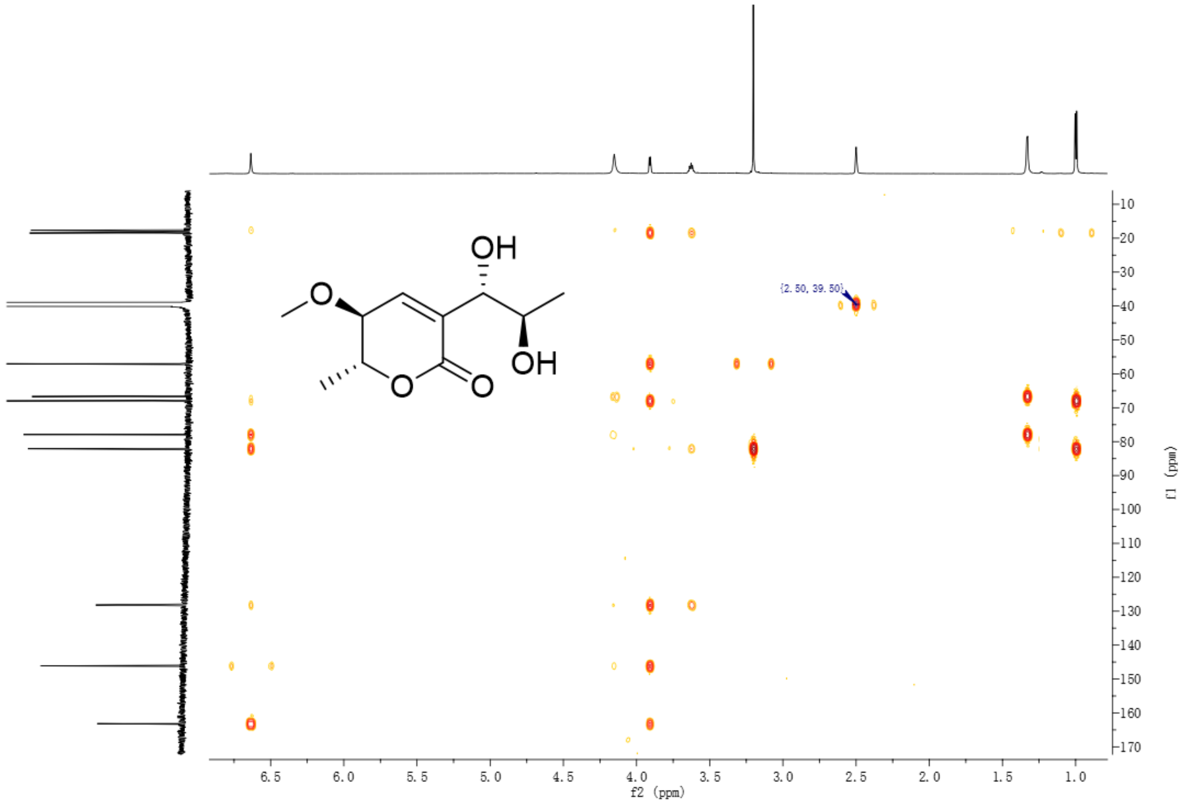

Figure S48. HMBC spectrum of 5-methoxy-8,9-dihydroxy-8,9-deoxyaspyrone (**21**)

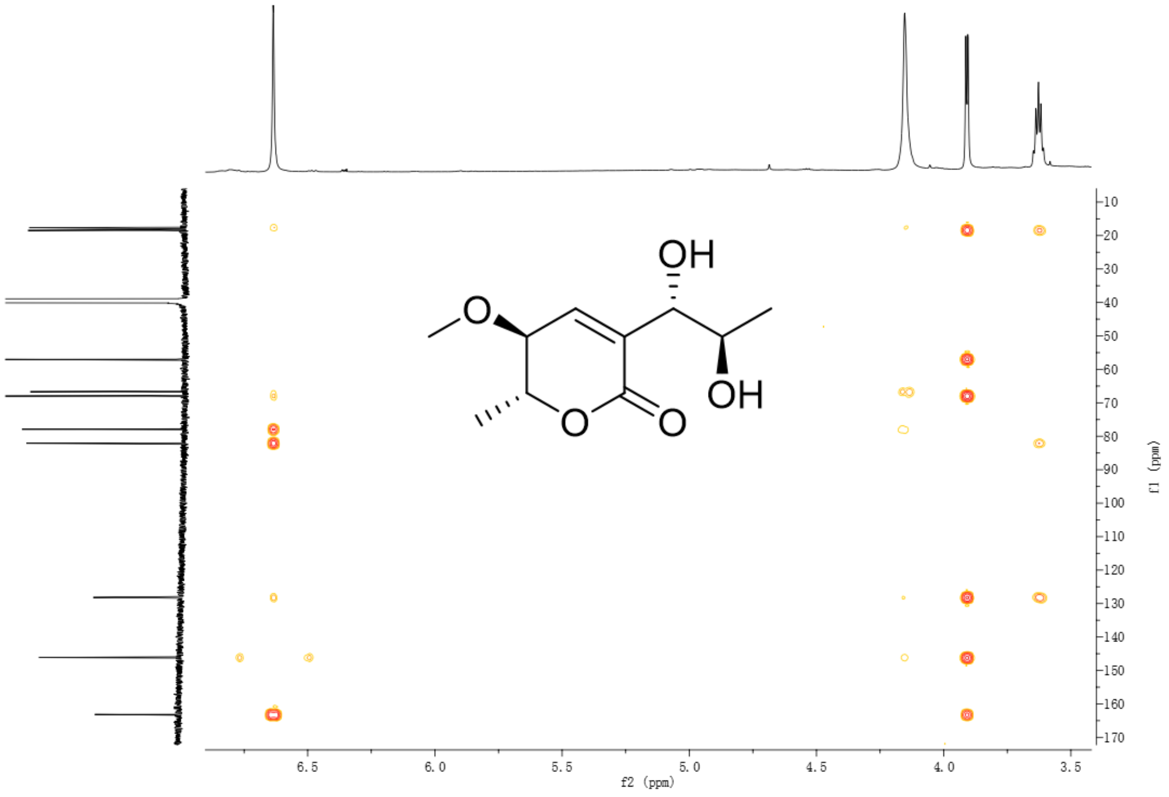

Figure S49. HMBC spectrum of 5-methoxy-8,9-dihydroxy-8,9-deoxyaspyrone (**21**)

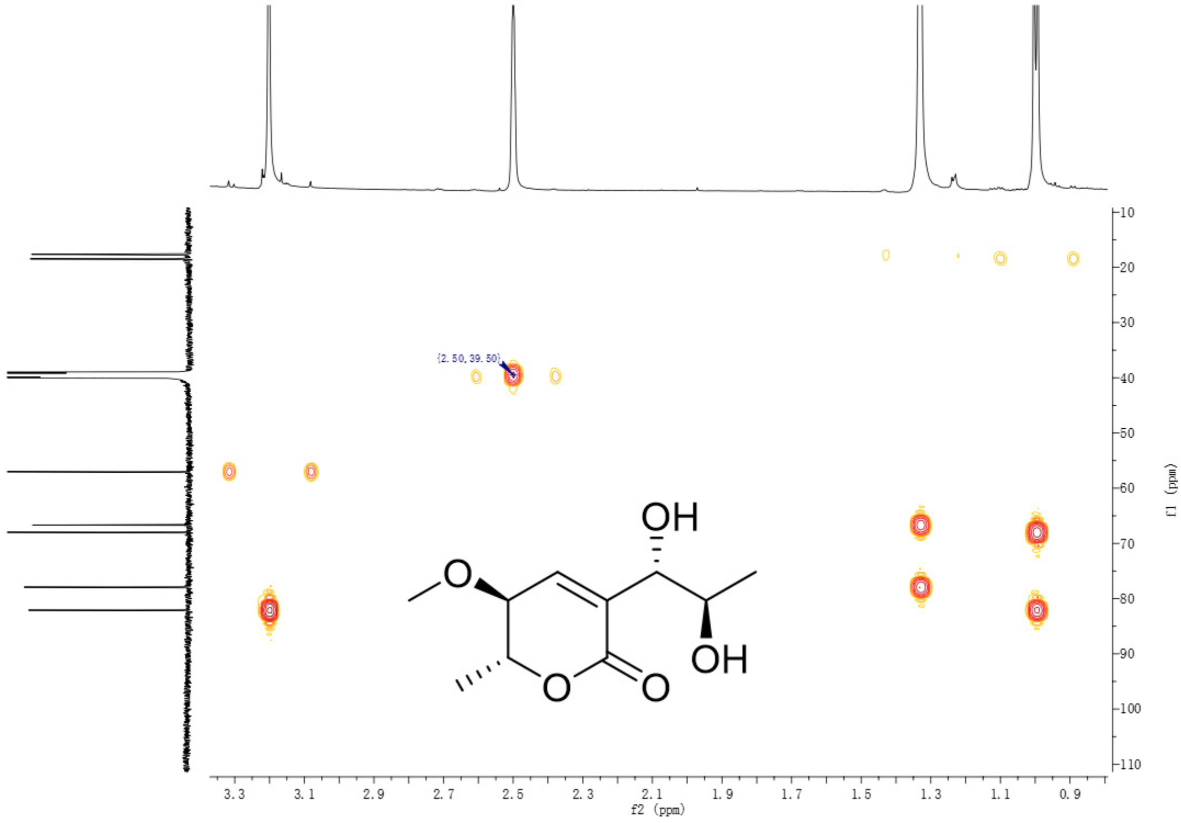

Figure S50. HRESIMS spectrum of 5-methoxy-8,9-dihydroxy-8,9-deoxyaspyrone (**21**)

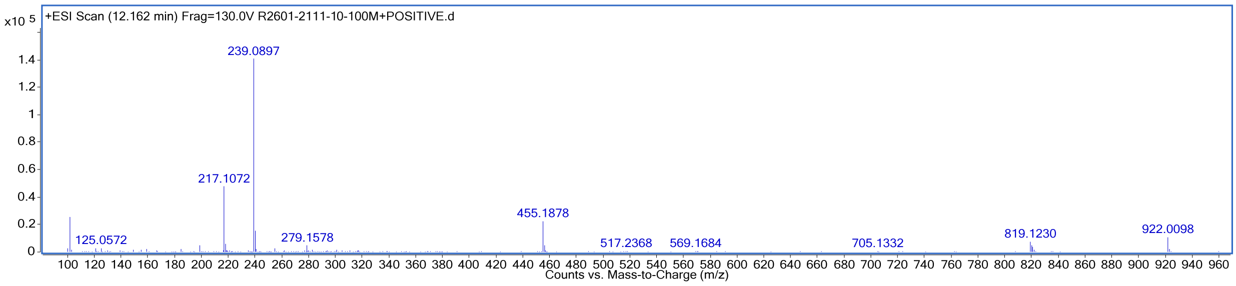

$[M+H]^+$: 217.1072 (calcd for $C_{10}H_{17}O_5$, 217.1076), $[M+Na]^+$: 239.0897 (calcd for $C_{10}H_{16}NaO_5$, 239.0895)

Figure S51. IR spectrum of 5-methoxy-8,9-dihydroxy-8,9-deoxyaspyrone (**21**)

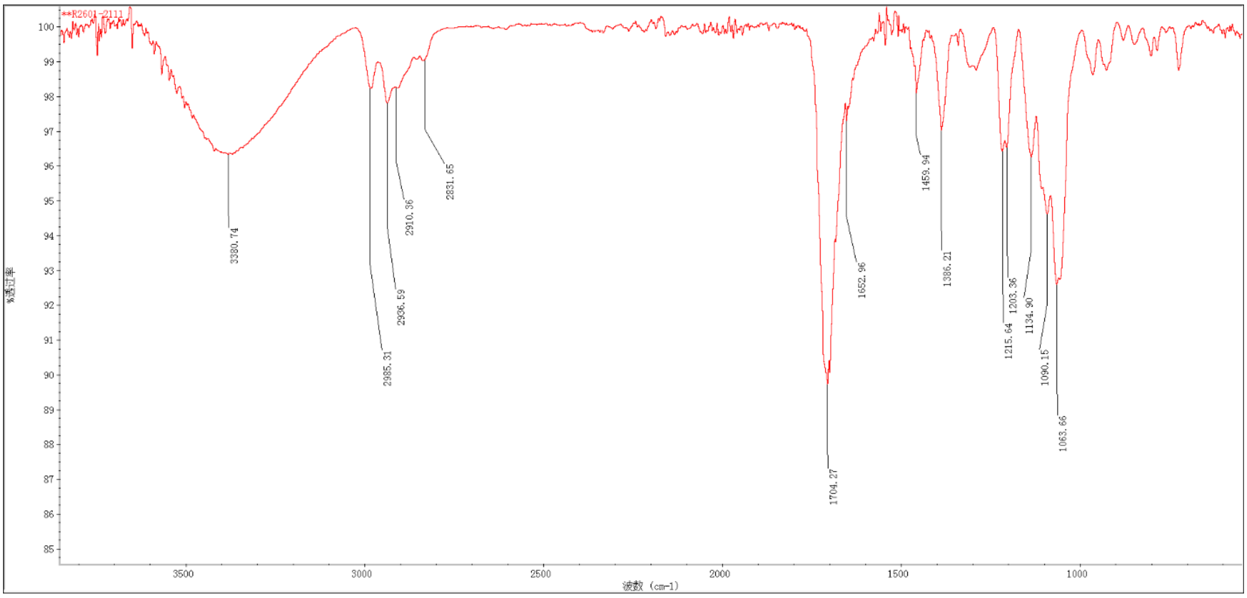

Figure S52. UV spectrum of 5-methoxy-8,9-dihydroxy-8,9-deoxyaspyrone (**21**)

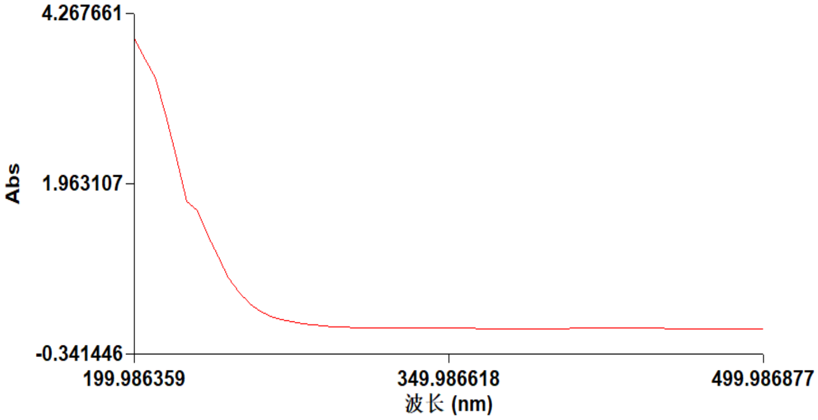

Figure S53. ^{1}H NMR spectrum of 12*S*-aspertetranone D (**26**)

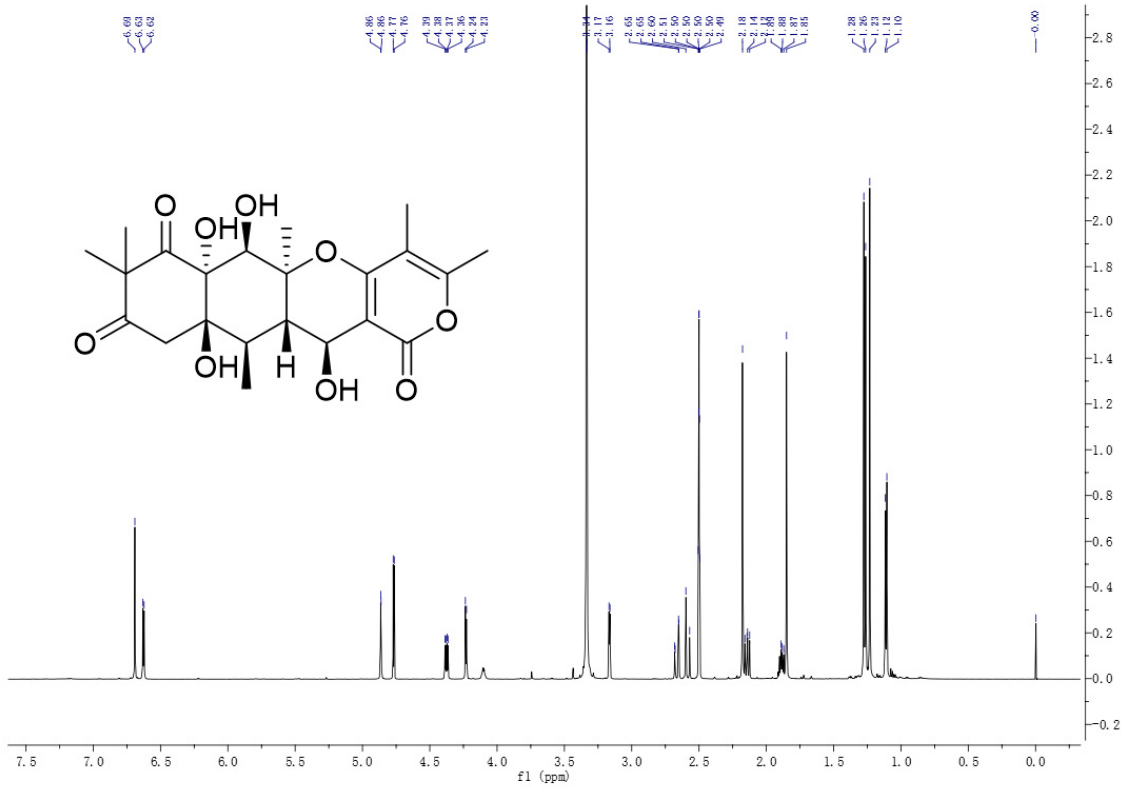

Figure S54. ^{1}H NMR spectrum of 12*S*-aspertetranone D (**26**)

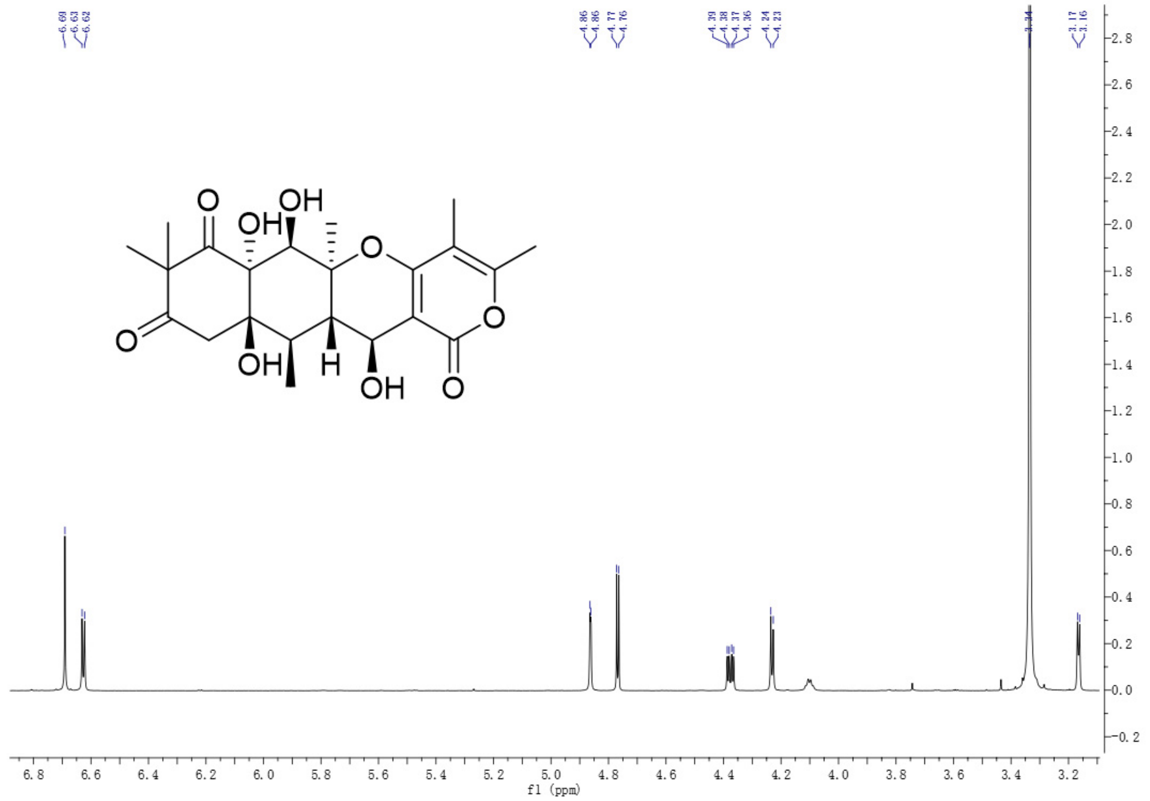

Figure S55. ^{1}H NMR spectrum of 12*S*-aspertetranone D (**26**)

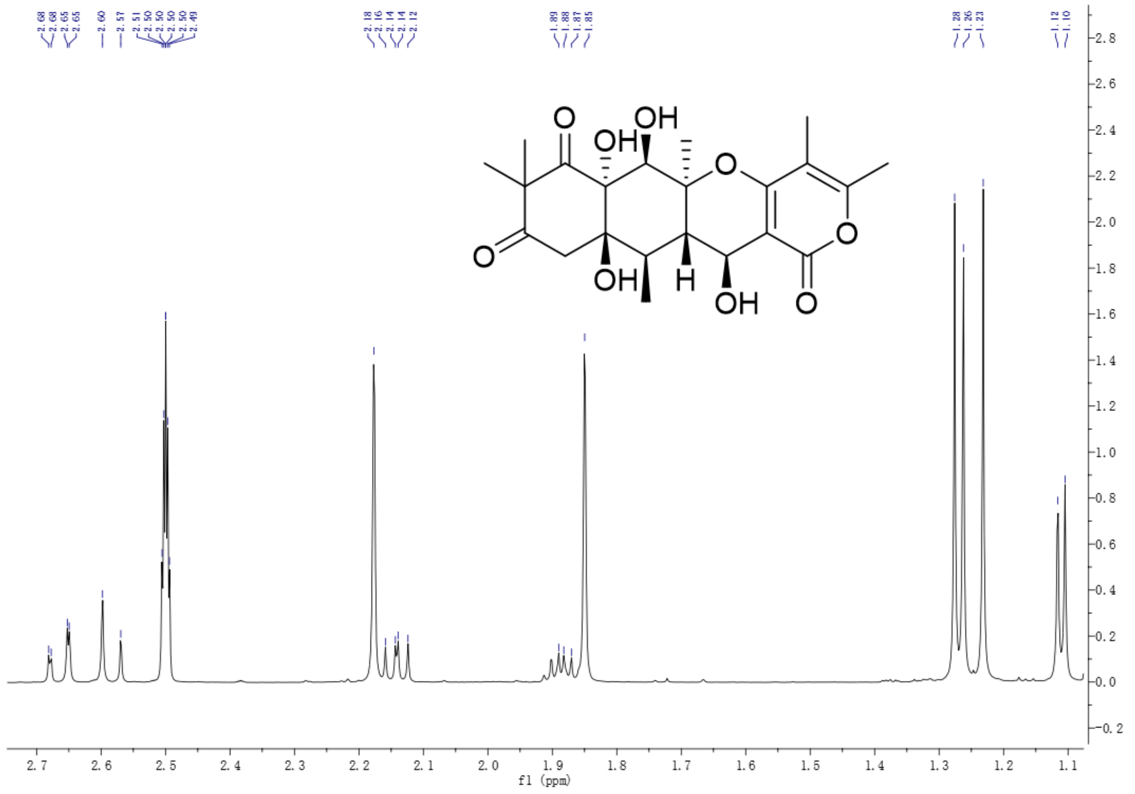

Figure S56. ^{13}C NMR spectrum of 12*S*-aspertetranone D (**26**)

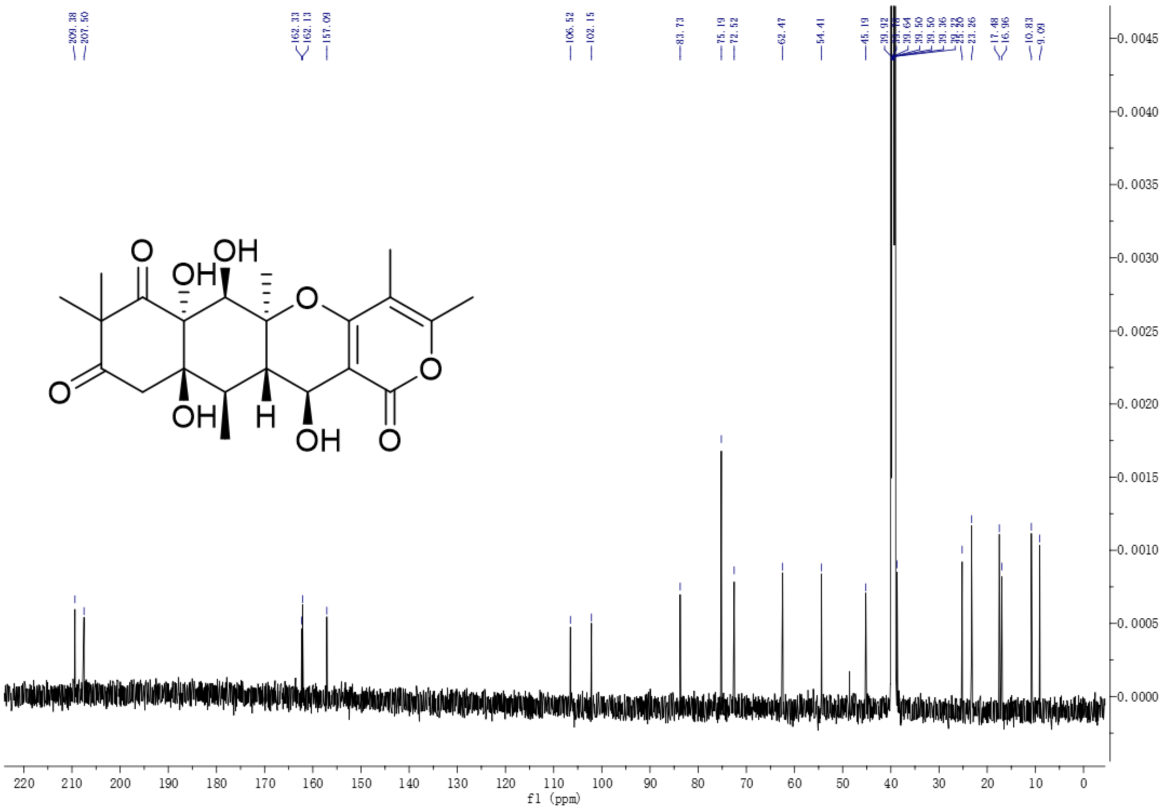

Figure S57. ^{13}C NMR spectrum of 12*S*-aspertetranone D (**26**)

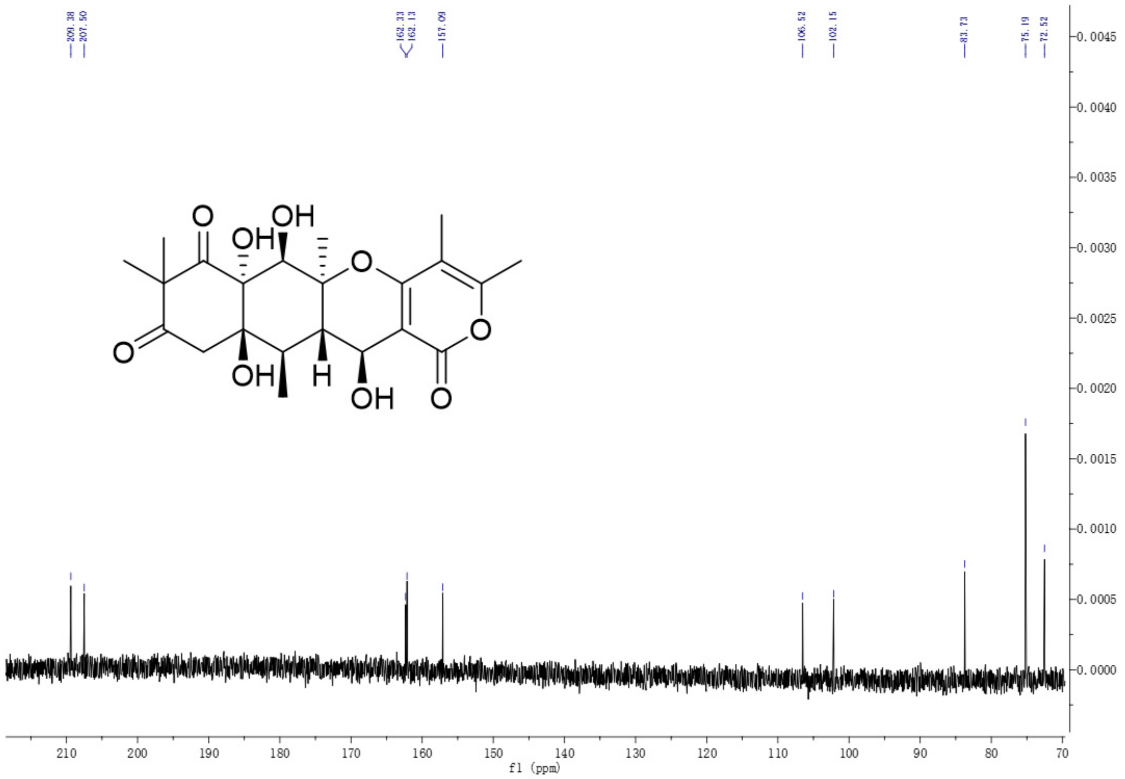

Figure S58. ^{13}C NMR spectrum of 12*S*-aspertetranone D (**26**)

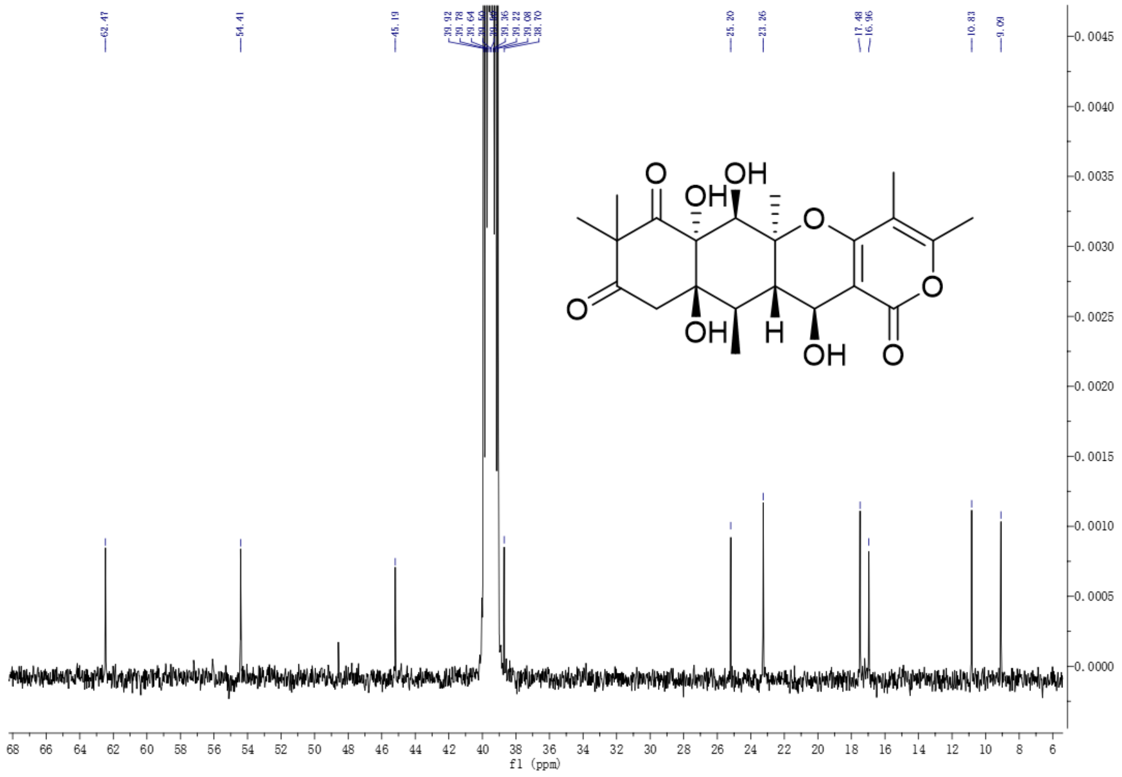

Figure S59. COSY spectrum of 12*S*-aspertetranone D (**26**)

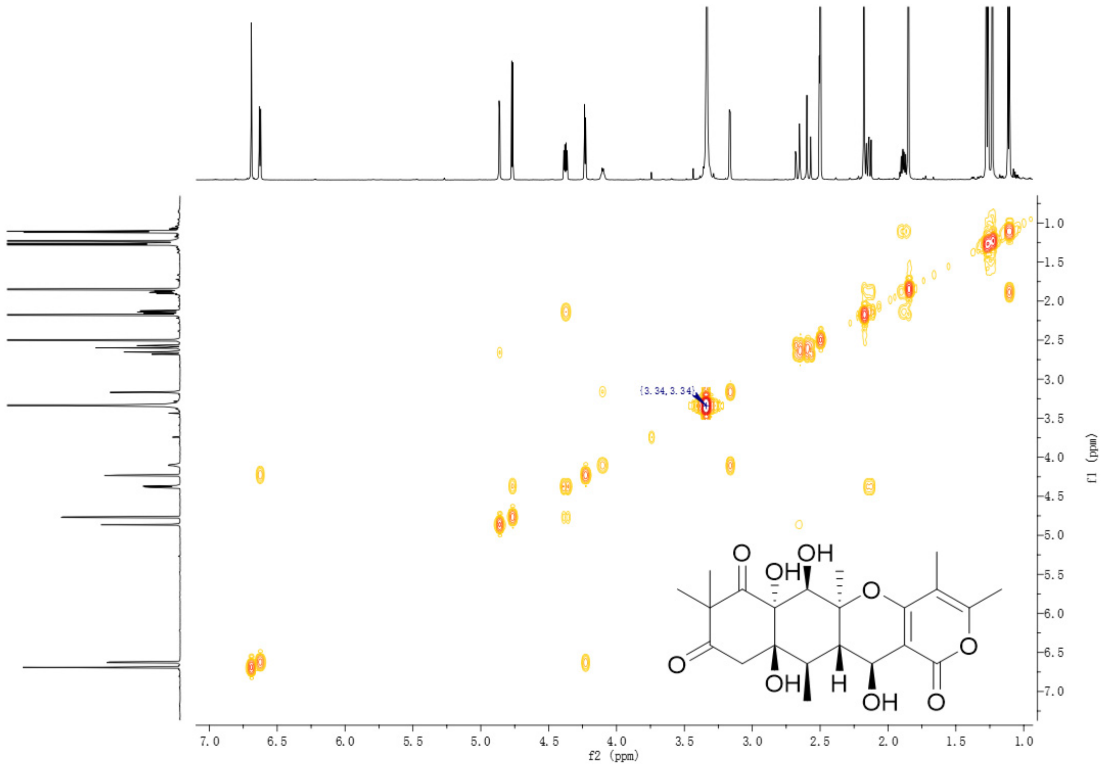

Figure S60. HMQC spectrum of 12*S*-aspertetranone D (**26**)

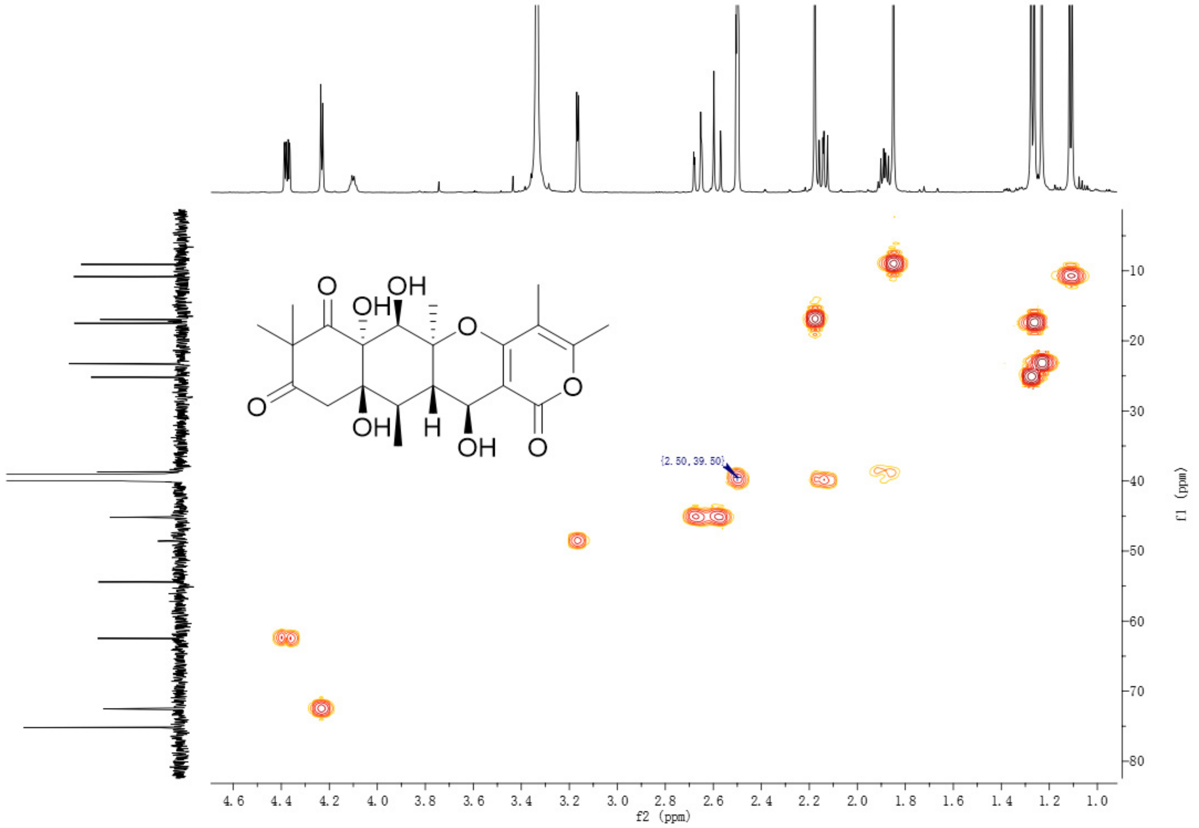

Figure S61. HRESIMS spectrum of 12*S*-aspertetranone D (**26**)

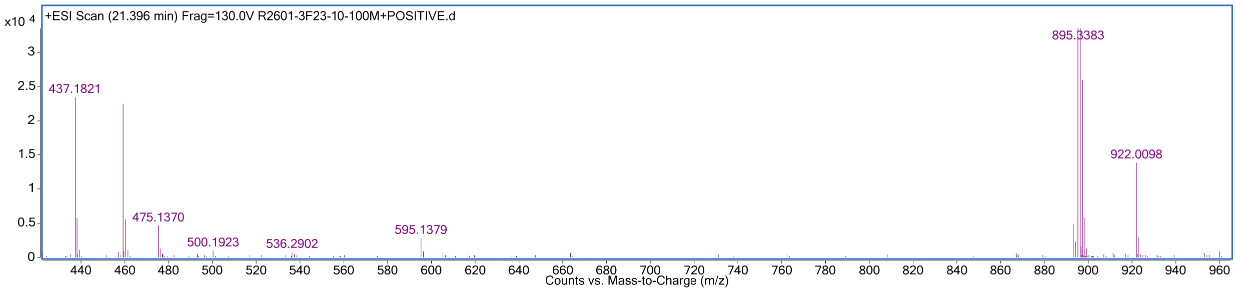

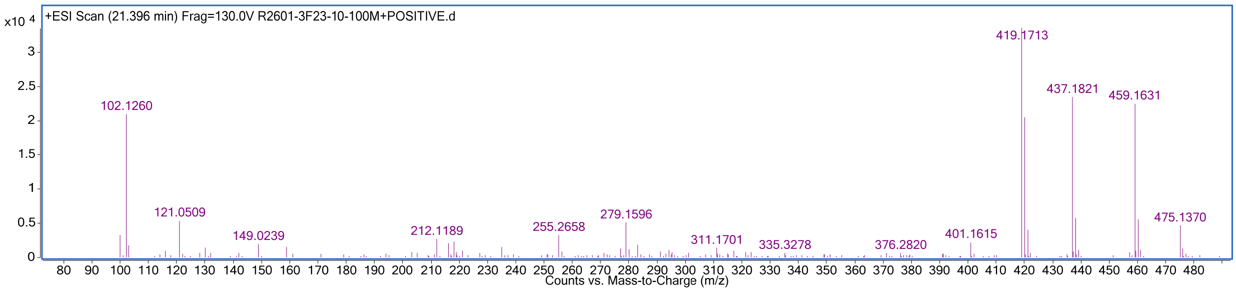

[M+H]$^+$: 437.1821 (calcd for $C_{22}H_{29}O_9$, 437.1812), [M+Na]$^+$ 459.1631 (calcd for $C_{22}H_{28}NaO_9$, 459.1631).

Figure S62. IR spectrum of 12*S*-aspertetranone D (**26**)

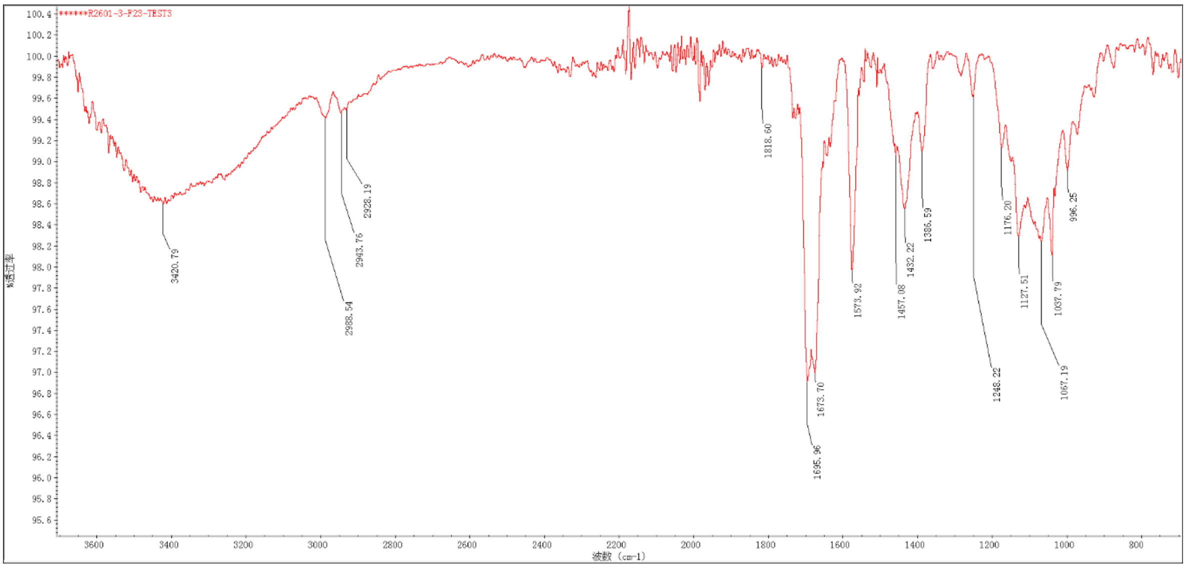

Figure S63. UV spectrum of 12*S*-aspertetranone D (**26**)

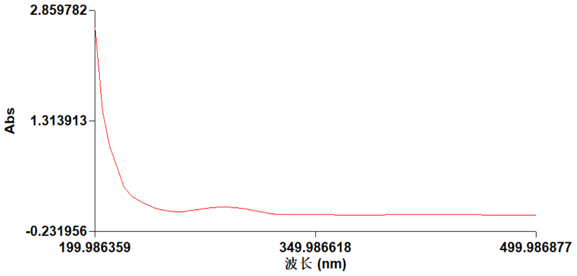

Table S1. Sequences producing significant alignments of strain *Aspergillus* sp. SY2601

Accession	Description	Max score	Total score	Query coverage (%)	E-value	Identity (%)
MT371256.1	*Aspergillus* sp. isolate 151304 small subunit ribosomal RNA gene, partial sequence	1072	1309	100	0	99.83
OP237490.1	*Aspergillus ochraceopetaliformis* strain 93N small subunit ribosomal RNA gene, partial sequence;	1072	1309	100	0	99.83
MN110110.1	*Aspergillus* sp. isolate 5S1 small subunit ribosomal RNA gene, partial sequence	1070	1309	100	0	99.83
MH857406.1	*Aspergillus ochraceopetaliformis* culture CBS:123.55 strain CBS 123.55 small subunit ribosomal RNA gene, partial sequence	1070	1309	100	0	99.83
MH141440.1	*Aspergillus ochraceopetaliformis* strain MF4 small subunit ribosomal RNA gene, partial sequence	1070	1309	100	0	99.83
OR234391.1	*Aspergillus* sp. isolate 2104NT-1.5 small subunit ribosomal RNA gene, partial sequence	1070	1309	100	0	99.83
OW988382.1	*Aspergillus ochraceopetaliformis* genomic DNA sequence contains 18S rRNA gene, ITS1, 5.8S rRNA gene, ITS2, 28S rRNA gene	1070	1309	100	0	99.83
OL772690.1	Aspergillus flocculosus strain CBS 112785 small subunit ribosomal RNA gene, partial sequence	1070	1309	100	0	99.83
MZ568132.1	*Aspergillus insulicola* isolate GP-22-1 small subunit ribosomal RNA gene, partial sequence	1070	1309	100	0	99.83
HQ649845.1	*Aspergillus* sp. r089 18S ribosomal RNA gene, partial sequence	1070	1309	100	0	99.83
HQ649847.1	*Aspergillus* sp. r192 18S ribosomal RNA gene, partial sequence	1068	1307	99	0	99.83

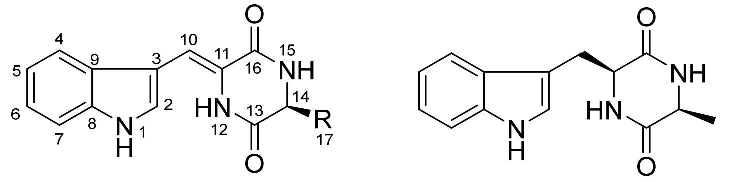

4 R = CH_3; **5** R = H **6**

Table S2. ^{13}C and ^{1}H NMR data (150 and 600 MHz) of 2-deisoprenyl-neoechinulin A (**4**), dipodazine (**5**), and cyclo-L-tryptophan-L-alanine (**6**)

No.	**4**[a]		**5**[a]		**6**[b]	
	δ_C, type	δ_H, J in Hz	δ_C, type	δ_H, J in Hz	δ_C, type	δ_H, J in Hz
1	–	11.67, br s	–	11.64, s	–	–
2	126.4, CH	7.94, s	126.2, CH	8.12, s	126.0, CH	7.07, s
3	107.9, C	–	107.8, C	–	109.5, C	–
4	118.1, CH	7.63, d (8.2)	118.0, CH	7.63, d (8.0)	120.1, CH	7.59, d (8.2)
5	119.9, CH	7.09, t (8.2)	119.9, CH	7.09, t (8.0)	120.3, CH	6.98, t (8.2)
6	122.1, CH	7.16, t (8.2)	122.0, CH	7.16, t (8.0)	122.6, CH	7.06, t (8.2)
7	111.8, CH	7.42, d (8.2)	111.8, CH	7.42, d (8.0)	112.3, CH	7.31, d (8.2)
8	135.7, C	–	135.6, C	–	138.0, C	–
9	127.0, C	–	127.0, C	–	129.4, C	–
10	107.6, CH	7.00, s	107.5, CH	7.00, s	30.9, CH_2	3.44, dd (14.8, 4.2);<br>3.13, dd (14.8, 4.4)
11	122.6, C	–	122.5, C	–	57.5, CH	4.26, t (4.4)
12	–	9.46, s	–	9.49, s	–	–
13	167.5, C	–	164.4, C	–	170.8, C	–
14	50.4, CH	4.12, qd (7.2, 1.6)	44.9, CH_2	4.00, d (2.7)	51.9, CH	3.68, q (7.3)
15	–	8.27, br s	–	7.93, d (2.7)	–	–
16	161.0, C	–	160.7, C	–	169.7, C	–
17	19.4, CH_3	1.34, d (7.2)	–	–	20.2, CH_3	0.35, d (7.3)

[a,b] The data were recorded in in DMSO-d_6 and in MeOH-d_4, respectively.

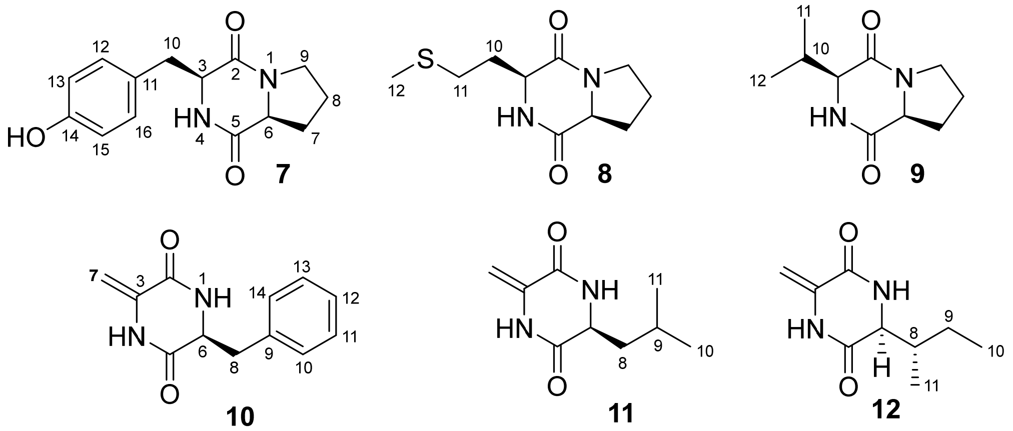

Table S3. ^{13}C NMR data (150 MHz) of cyclo-L-proline-L-tyrosine (**7**), cyclo-L-proline-L-methionine (**8**), cyclo-L-proline-L-valine (**9**), (6*S*)-3-methylene-6-benzyl-2,5-piperazinedione (**10**), (6*S*)-3-methylene-6-(2-methylpropyl)-2,5-piperazinedione (**11**), and (6*S*,8*S*)-3-methylene-6-(1-methylpropyl)-2,5-piperazinedione (**12**)

No.	**7**^{a}	**8**^{a}	**9**^{a}	**10**^{a}	**11**^{b}	**12**^{a}
2	167.1, C	168.0, C	167.7, C	161.4, C	158.3, C	161.7, C
3	58.0, CH	55.5, CH	60.2, CH	135.0, C	134.7, C	135.6, C
5	171.0, C	172.7, C	172.7, C	167.9, C	166.5, C	168.0, C
6	60.2, CH	60.5, CH	61.7, CH	58.4, CH	53.7, CH	61.9, CH
7	29.5, CH_2	29.4, CH_2	29.7, CH_2	101.6, CH_2	99.0, CH_2	101.8, CH_2
8	22.9, CH_2	23.6, CH_2	23.4, CH_2	41.4, CH_2	43.6, CH_2	42.4, CH
9	46.1, CH_2	46.6, CH_2	46.3, CH_2	136.0, C	23.4, CH	25.6, CH_2
10	37.7, CH_2	30.4 CH_2	30.0, CH	131.6, CH	22.2^c, CH_3	12.3, CH_3
11	127.8, C	30.5, CH_2	19.0^c, CH_3	129.6, CH	22.7^c, CH_3	15.4, CH_3
12	132.1, CH	15.3, CH_3	16.8^c, CH_3	128.4, CH_3		
13	116.3, CH			129.6, CH		
14	157.8, C			131.6, CH		
15	116.3, CH					
16	132.1, CH					

^{a,b} The data were recorded in MeOH-d_4 and DMSO-d_6, respectively. ^{c} The data with the same label in each column may be interchanged.

Table S4. ^{1}H NMR data (600 MHz) of cyclo-L-proline-L-tyrosine (**7**), cyclo-L-proline-L-methionine (**8**), cyclo-L-proline-L-valine (**9**), (6*S*)-3-methylene-6-benzyl-2,5-piperazinedione (**10**), (6*S*)-3-methylene-6-(2-methylpropyl)-2,5-piperazinedione (**11**), and (6*S,8S*)-3-methylene-6-(1-methylpropyl)-2,5-piperazinedione (**12**)

No.	**7**[a]	**8**[a]	**9**[a]	**10**[a]	**11**[b]	**12**[a]
1	–	–	–	–	8.46, 1H, br s	–
3	5.84, 1H, t (4.7)	4.27, 1H, t (5.5)	4.04, 1H, t (2.8)	–	–	–
4	–	–	–	–	10.50, 1H, br s	–
6	5.51, 1H, m	4.23, 1H, t (7.7)	4.19, 1H, t (7.5)	4.41, 1H, t (4.6)	3.96, 1H, dd (6.5, 2.5)	4.02, 1H, d (3.7)
7	3.56, 1H, m; 2.73, 1H, m	2.29, 2H, m	2.30, 1H, m; 2.01, 1H, m	5.07, 1H, s; 4.57, 1H, s	5.17, 1H, s; 4.77, 1H, s	5.42, 1H, s; 4.91, 1H, s
8	3.26, 2H, m	2.02, 2H, m	1.92, 2H, m	3.23, 1H, dd (13.7, 4.6); 3.00, 1H, dd (13.7, 4.6)	1.56, 2H, m	1.92, 1H, m
9	5.01, 1H, m; 4.81, 1H, m	3.50, 2H, m	3.54, 1H, m; 3.48, 1H, m	–	1.78, 1H, m	1.47, 1H, m; 1.26, 1H, m
10	4.56, 1H, dd (14.3, 5.3); 4.52, 1H, dd (14.3, 4.7)	2.19, 1H, m; 2.07, 1H, m	2.46, 1H, m	7.15, 2H, dd (8.3, 2.3)	0.85[c], 3H, d (6.5)	0.93, 3H, t (7.2)
11	–	2.60, 2H, m	1.09[c], 3H, d (6.9)	7.22–7.25, 3H, m	0.86[c], 3H, d (6.8)	0.99, 3H, d (7.2)
12	8.53, 1H, d (8.3)	2.10, 3H, s	0.93[c], 3H, d (7.3)	7.22–7.25, 3H, m		
13	8.20, 1H, d (8.3)			7.22–7.25, 3H, m		
14	–			7.15, 2H, dd (8.3, 2.3)		
15	8.20, 1H, d (8.3)					
16	8.53, 1H, d (8.3)					

[a,b] The data were recorded in MeOH-d_4 and DMSO-d_6, respectively. [c] The data with the same label in each column may be interchanged.

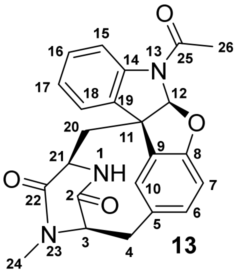

13

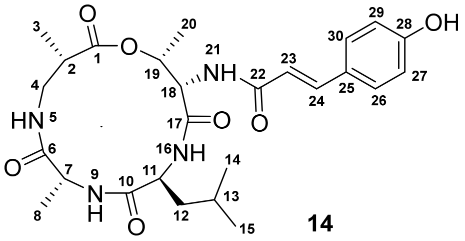

14

Table S5. ^{13}C and ^{1}H NMR data (150 and 600 MHz, in DMSO-d_6) of azonazine (**13**) and aspergillipeptide A (**14**)

No.	**13**		**14**	
	δ_C, type	δ_H, J in Hz	δ_C, type	δ_H, J in Hz
1	–	8.77, 1H, s	171.8, C	–
2	168.1, C	–	39.8, CH	2.65, 1H, m
3	64.8, CH	4.15, 1H, d (7.0)	15.0, CH_3	1.04, 3H, d (7.3)
4	37.7, CH_2	3.37, 1H, d (13.6); 3.04, 1H, dd (13.6, 7.0)	41.2, CH_2	3.23, 2H, m
5	131.7, C	–	–	7.34, 1H, t (6.3)
6	130.3, CH	6.96, 1H, dd (8.0, 1.5)	172.0, C	–
7	109.5, CH	6.70, 1H, d (8.0)	49.2, CH	3.91, 1H, m
8	157.1, C	–	16.5, CH_3	1.23, 3H, d (7.6)
9	130.5, C	–	–	8.82, 1H, d (7.2)
10	124.6, CH	7.35, 1H, d (1.5)	171.8, C	–
11	57.3, C	–	52.0, CH	4.18, 1H, dd (14.2, 7.3)
12	105.3, CH	6.72, 1H, s	38.3, CH_2	1.45, 2H, m
13	–	–	24.1, CH	1.53, 1H, m
14	141.0, C	–	22.4[a], CH_3	0.93[a], 3H, d (6.5)
15	115.6, CH	7.62, 1H, d (8.0)	22.0[a], CH_3	0.86[a], 3H, d (6.5)
16	128.3, CH	7.19, 1H, t (8.0)	–	8.79, 1H, d (6.4)
17	124.4, CH	7.27, 1H, t (8.0)	171.0, C	–
18	123.1, CH	8.05, 1H, d (8.0)	54.0, CH	4.80, 1H, dd (9.5, 2.0)
19	134.2, C	–	71.5, CH	5.04, 1H, qd (6.5, 2.0)
20	41.1, CH_2	2.80, 1H, dd (16.1, 5.3); 2.47, 1H, dd (16.1, 2.5)	16.0, CH_3	1.08, 1H, d (6.5)
21	53.4, CH	4.24, 1H, d (5.3)	–	8.06, 1H, d (9.5)
22	164.2, C	–	165.9, C	–
23	–	–	117.5, CH	6.82, 1H, d (15.5)
24	31.7, CH_3	2.36, 3H, s	139.8, CH	7.35, 1H, d (15.5)
25	170.1, C	–	125.1, C	–
26	23.6, CH_3	2.41, 3H, s	129.3, CH	7.42, 1H, d (8.5)
27			115.8, CH	6.78, 1H, d (8.5)
28			159.8, C	–
29			115.8, CH	6.78, 1H, d (8.5)
30			129.3, CH	7.42, 1H, d (8.5)

[a] The data with the same label in each column may be interchanged.

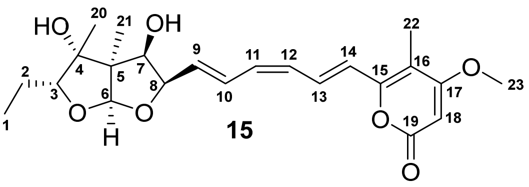

15

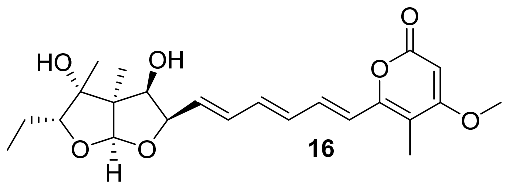

16

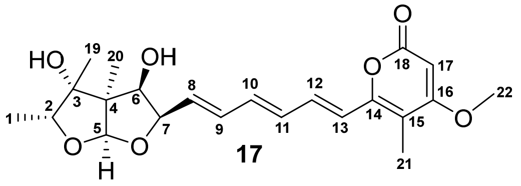

17

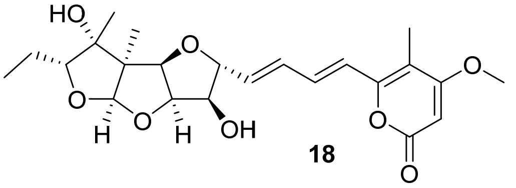

18

Table S6. ^{13}C NMR data (150 MHz) of isoasteltoxin (**15**), asteltoxin (**16**), and asteltoxins C (**17**) and B (**18**)

No.	**15**^{a}	**16**^{b}	**17**^{a}	**18**^{b}
1	11.7, CH_3	11.5, CH_3	13.3, CH_3	11.4, CH_3
2	22.8, CH_2	21.5, CH_2	85.1, CH	22.1, CH_2
3	91.3, CH	89.0, CH	81.8, C	90.4, CH
4	81.9, C	79.4, C	62.7, C	80.5, C
5	63.0, C	61.3, C	113.3, CH	61.7, C
6	113.4, CH	111.5, CH	80.3, CH	113.3, CH
7	80.6, CH	78.5, CH	86.0, CH	86.7, CH
8	86.3, CH	84.0, CH	133.3, CH	86.1, CH
9	134.3, CH	134.8, CH	133.4, CH	76.7, CH
10	129.7, CH	131.7, CH	138.6, CH	78.8, CH
11	135.0, CH	137.4, CH	134.4, CH	137.7, CH
12	129.8, CH	132.1, CH	136.9, CH	130.6, CH
13	131.7, CH	134.1, CH	120.9, CH	133.9, CH
14	121.5, CH	120.2, CH	155.8, C	120.7, CH
15	155.7, C	153.7, C	110.0, C	153.5, C
16	110.5, C	108.0, C	173.1, C	108.2, C
17	173.1, C	170.2, C	89.4, CH	170.1, C
18	89.4, CH	88.8, CH	166.3, C	88.9, CH
19	166.4, C	162.1, C	17.4, CH_3	162.0, C
20	17.8, CH_3	17.8, CH_3	16.8, CH_3	18.4, CH_3
21	16.6, CH_3	16.0, CH_3	8.9, CH_3	14.7, CH_3
22	8.9, CH_3	8.7, CH_3	57.3, CH_3	8.6, CH_3
23	57.3, CH_3	56.8, CH_3		56.7, CH_3

^{a,b} The data were recorded in MeOH-d_4 and DMSO-d_6, respectively.

Table S7. ^{1}H NMR data (600 MHz) of isoasteltoxin (**15**), asteltoxin (**16**), and asteltoxins C (**17**) and B (**18**)

No.	**15**[a]	**16**[b]	**17**[a]	**18**[b]
1	1.03, 3H, t (7.7)	0.89, 3H, t (7.5)	1.14, 3H, d (6.4)	0.90, 3H, t (7.2)
2	1.55, 2H, m	1.35, 2H, m	4.62, 1H, q (6.4)	1.39, 2H, m
3	4.39, 1H, dd (9.2, 3.5)	4.15, 1H, dd (9.0, 3.6)	–	3.88, 1H, dd (9.1, 3.8)
5	–	–	5.21, 1H, s	–
6	5.24, 1H, s	5.03, 1H, s	3.74, 1H, d (3.1)	5.26, 1H, s
7	3.76, 1H, d (2.8)	3.59, 1H, d (2.8)	4.64, 1H, dd (7.0, 3.1)	4.15, 1H, d (3.8)
8	4.69, 1H, dd (7.5, 2.8)	4.51, 1H, dd (7.4, 2.8)	6.04, 1H, dd (15.1, 7.0)	4.60, 1H, t (3.8)
9	6.07, 1H, dd (15.4, 7.5)	5.98, 1H, dd (15.2, 7.4)	6.49, 1H, dd (15.1, 10.7)	3.71, 1H, br t (8.2)
10	7.02, 1H, dd (15.4, 11.3)	6.65, 1H, dd (15.2, 11.2)	6.61, 1H, dd (14.9, 10.7)	4.24, 1H, t (7.7)
11	6.34, 1H, t (11.3)	6.48, 1H, dd (14.9, 11.2)	6.51, 1H, dd (14.9, 11.1)	6.06, 1H, dd (15.1, 7.0)
12	6.28, 1H, t (11.5)	6.34, 1H, dd (14.9, 11.1)	7.13, 1H, dd (14.9, 11.1)	6.46, 1H, dd (15.1, 11.0)
13	7.64, 1H, dd (14.8, 11.3)	6.98, 1H, dd (15.0, 11.1)	6.59, 1H, d (14.9)	6.92, 1H, dd (15.1, 11.0)
14	6.62, 1H, d (14.8)	6.66, 1H, d (15.0)	–	6.73, 1H, d (15.0)
17	–	–	5.64, 1H, s	–
18	5.68, 1H, s	5.62, 1H, s	–	5.64, 1H, s
19	–	–	1.34, 3H, s	–
20	1.37, 3H, s	1.19, 3H, s	1.20, 3H, s	1.20, 3H, s
21	1.21, 3H, s	1.03, 3H, s	2.02, 3H, s	0.94, 3H, s
22	2.04, 3H, s	1.93, 3H, s	3.92, 3H, s	1.94, 3H, s
23	3.93, 3H, s	3.83, 3H, s	–	3.84, 3H, s
OH-9	–	–	–	5.17, d (8.2)

[a,b] The data were recorded in MeOH-d_4 and DMSO-d_6, respectively.

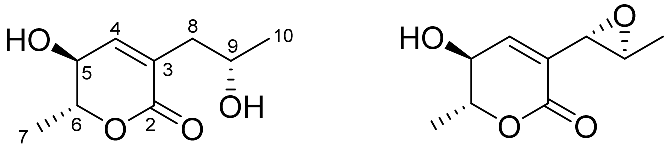

19 **20**

Table S8. ^{13}C and ^{1}H NMR data (150 and 600 MHz) of dihydroaspyrone (**19**) and aspyrone (**20**)

No.	**19**[a]		**20**[b]	
	δ_C, type	δ_H, J in Hz	δ_C, type	δ_H, J in Hz
2	165.2, C	–	162.8, C	–
3	129.3, C	–	127.3, C	–
4	144.2, CH	6.64, d (2.7)	143.5, CH	6.53, d (2.1)
5	67.6, CH	4.19, dd (7.5, 2.7)	66.2, CH	4.07, d (9.1)
6	79.4, CH	4.38, dq (7.5, 6.5)	78.9, CH	4.22, dq (9.1, 6.5)
7	18.1, CH_3	1.46, d (6.5)	17.8, CH_3	1.31, d (6.5)
8	39.7, CH_2	2.44, dd (13.8, 4.0); 2.40, dd (13.8, 7.0)	54.1, CH	3.37, d (1.8)
9	66.9, CH	4.01, m	57.6, CH	2.87, qd (4.7, 1.8)
10	23.3, CH_3	1.23, d (6.1)	17.4, CH_3	1.29, d (4.7)
OH-5	–	3.21, d (7.5)	–	5.78, br s
OH-9	–	2.59, br s	–	–

[a,b] The data were recorded in $CDCl_3$ and DMSO-d_6, respectively.

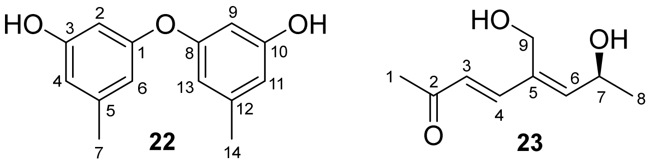

Table S9. ^{13}C and ^{1}H NMR data (150 and 600 MHz) of diorcinol (**22**) and aspinonediol (**23**) (in DMSO-d_6)

No.	**22**		**23**	
	δ_C, type	δ_H, J in Hz	δ_C, type	δ_H, J in Hz
1	158.4, C	–	26.9, CH_3	2.23, s
2	102.9, CH	6.15, s	198.3, C	–
3	157.6, C	–	127.2, CH	6.25, d (16.2)
4	111.1, CH	6.24, s	146.1, CH	7.10, d (16.2)
5	140.1, C	–	135.0, C	–
6	110.0, CH	6.33, s	147.7, CH	5.99, d (8.2)
7	21.1, CH_3	2.18, s	62.6, CH	4.61, dq (8.2, 6.4)
8	158.4, C	–	23.8, CH_3	1.15, d (6.4)
9	102.9, CH	6.15, s	55.4, CH^2	4.14, s
10	157.6, C	–		
11	111.1, CH	6.24, s		
12	140.1, C	–		
13	110.0, CH	6.33, s		
14	21.1, CH_3	2.18, s		
OH-3	–	9.44, s		

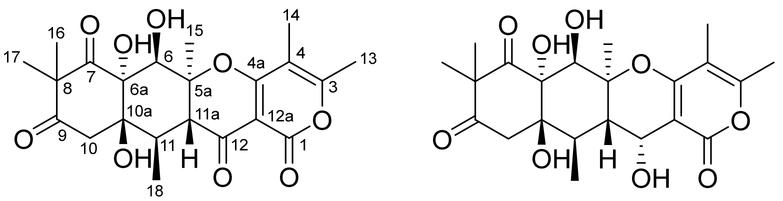

24 **25**

Table S10. ^{13}C and ^{1}H NMR data (150 and 600 MHz) of aspertetranones A (**24**) and D (**25**)

No.	**24**[a]		**25**[a]		**25**[b]	
	δ_C, type	δ_H, J in Hz	δ_C, type	δ_H, J in Hz	δ_C, type	δ_H, J in Hz
1	156.5, C	–	162.8, C	–	163.2, C	–
3	163.9, C	–	156.7, C	–	157.1, C	–
4	106.1, C	–	106.3, C	–	106.6 C	–
4a	170.8, C	–	162.2, C	–	162.2, C	–
5a	87.3, C	–	81.8, C	–	82.1, C	–
6	71.8, CH	4.43, d (5.5)	73.1, CH	4.24, d (6.0)	74.5, CH	4.54, d (4.5)
6a	75.0, C	–	75.3, C	–	76.0, C	–
7	207.4, C	–	207.7, C	–	208.2, C	–
8	54.5, C	–	54.5, C	–	55.0, C	–
9	209.2, C	–	209.5, C	–	208.7, C	–
10	44.9, CH_2	2.71, dd (16.8, 2.8); 2.60, d (16.8)	45.4, CH_2	2.74, dd (16.5, 1.7); 2.61, d (16.5)	45.4, CH_2	2.86, dd (17.5, 2.7); 2.73, d (17.5)
10a	74.5, C	–	75.1, C	–	75.3, C	–
11	33.5, CH	1.94, dd (11.8, 6.6)	34.2, CH	2.01, dd (11.8, 6.6)	34.5, CH	2.31, dd (11.8, 6.4)
11a	47.7, CH	3.17, d (11.8)	38.9, CH	1.92, d (11.8, 3.9)	39.2, CH	2.21, d (11.8, 4.0)
12	189.7, C	–	57.7, CH	4.48, dd (5.4, 3.9)	59.0, CH	4.71, t (4.0)
12a	99.6, C	–	101.0, C	–	101.6, C	–
13	17.8, CH_3	2.23, s	17.0, CH_3	2.17, s	16.5, CH_3	2.20, s
14	9.1, CH_3	1.91, s	9.3, CH_3	1.85, s	8.7, CH_3	1.90, s
15	17.9, CH_3	1.34, s	20.5, CH_3	1.43, s	20.1, CH_3	1.64, s
16	23.0, CH_3	1.22, s	23.2, CH_3	1.22, s	22.9, CH_3	1.29, s
17	25.3, CH_3	1.29, s	25.4, CH_3	1.29, s	25.2, CH_3	1.31, s
18	11.3, CH_3	1.11, d (6.6)	9.9, CH_3	1.01, d (6.6)	9.6, CH_3	1.20, d (6.4)
OH-6	–	7.12, d (5.5)	–	6.54, d (6.0)	–	5.67, d (4.5)
OH-6a	–	6.77, s	–	6.62, s	–	5.69, s
OH-10a	–	4.88, d (2.8)	–	4.79, d (1.7)	–	4.66, d (2.7)
OH-12	–	–	–	5.07, d (5.4)	–	3.94, d (4.0)

[a,b] The data were recorded in DMSO-d_6 and acetone-d_6, respectively.

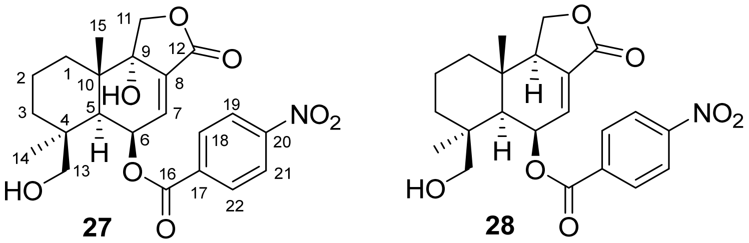

Table S11. ^{13}C and ^{1}H NMR data (150 and 600 MHz) of insolicolide A (**27**) and 9-deoxyinsolicolide (**28**)

No.	**27**		**28**	
	δ_C, type	δ_H, *J* in Hz	δ_C, type	δ_H, *J* in Hz
1	31.8, CH_2	2.07[a], m; 1.26, d (13.5)	34.2, CH_2	1.67, m; 1.38, m
2	17.2, CH_2	1.52, m; 1.41, m	17.6, CH_2	1.53, m; 1.34, m
3	35.6, CH_2	2.05[a], m; 0.78, dd (13.3, 3.1)	39.5, CH_2	2.08, br d (13.0); 0.86, dd (13.0, 2.5)
4	38.7, C	–	40.3, C	–
5	45.7, CH	2.38, d (4.5)	53.5, CH	1.84, d (4.5)
6	67.1, CH	5.97, t (4.5)	67.5, CH	6.04, m
7	131.2, CH	6.65, d (4.5)	129.1, CH	6.61, dd (4.5, 3.7)
8	133.4, C	–	131.4, C	–
9	75.9, C	–	50.8, CH	2.98, m
10	38.9, C	–	35.7, C	–
11	74.6, CH_2	4.48, d (10.0); 4.17, d (10.0)	67.5, CH_2	4.51, t (9.0); 4.15, t (9.0)
12	168.6, C	–	169.1, C	–
13	62.9, CH_2	3.86, dd (10.9, 5.5); 3.12, dd (10.9, 5.5)	63.0, CH_2	3.84, dd (10.6, 5.7); 3.23, dd (10.6, 5.7)
14	26.9, CH_3	0.99, s	26.5, CH_3	0.99, s
15	20.9, CH_3	1.23, s	16.4, CH_3	1.14, s
16	163.4, C	–	163.3, C	–
17	134.8, C	–	134.0, C	–
18	130.9, CH	8.16, d (8.3)	130.9, CH	8.16, d (8.5)
19	124.1, CH	8.38, d (8.3)	124.1, CH	8.39, d (8.5)
20	150.5, C	–	150.4, C	–
21	124.1, CH	8.38, d (8.3)	124.1, CH	8.39, d (8.5)
22	130.9, CH	8.16, d (8.3)	130.9, CH	8.16, d (8.5)
OH-13	–	4.29, d (5.5)	–	4.29, d (5.7)

[a] The data were overlapped.

Table S12. Gibbs free energies[a] and equilibrium populations[b] of low-energy conformers of 14*R*-**1**

Conformers	In MeOH	
	E	Optical rotation (OR)
14*R*-**1a**	-1008.8250036	85.15
14*R*-**1b**	-1008.8252284	80.05
14*R*-**1c**	-1008.8231179	140.31
Average		85.56

[a] B3LYP/6-31+G (d, p), in kcal/mol; [b] from Δ*G* values at 298.15K.

Table S13. Cartesian coordinates for the low-energy reoptimized MMFF conformers of 14*R*-**1** at B3LYP/6-311+G (d, p) level of theory in MeOH

14*R*-**1a**			Standard Orientation (Ångstroms)		
No.	Atom	Type	X	Y	Z
1	6	0	-4.553044	-1.992839	-0.371113

2	6	0	-4.995085	-0.739996	0.045454
3	6	0	-4.045127	0.282558	0.130817
4	6	0	-2.667384	0.08046	-0.168026
5	6	0	-2.259752	-1.188827	-0.623704
6	6	0	-3.201114	-2.210344	-0.712242
7	7	0	-4.208524	1.618538	0.451597
8	6	0	-3.006655	2.264616	0.359592
9	6	0	-2.008269	1.365724	0.005789
10	7	0	0.405816	-0.283508	0.70753
11	6	0	0.476139	0.979845	0.087067
12	6	0	-0.61923	1.736879	-0.157125
13	6	0	1.417702	-1.180539	0.788408
14	6	0	2.658104	-0.811281	-0.025812
15	7	0	2.846907	0.61874	-0.101471
16	6	0	1.835344	1.521847	-0.184897
17	6	0	3.98913	-1.302239	0.544186
18	6	0	5.004295	-0.353571	-0.116425
19	6	0	4.268776	0.997061	-0.189564
20	8	0	1.332601	-2.244014	1.406729
21	8	0	2.042774	2.718631	-0.433806
22	8	0	2.435651	-1.282977	-1.370886
23	1	0	-5.262528	-2.810588	-0.448661
24	1	0	-6.038279	-0.558942	0.283595
25	1	0	-1.232062	-1.370864	-0.916336
26	1	0	-2.891193	-3.191893	-1.057092
27	1	0	-5.08148	2.056289	0.70779
28	1	0	-2.926972	3.321197	0.571035
29	1	0	-0.455308	-0.531659	1.182895
30	1	0	-0.408179	2.753075	-0.475818
31	1	0	3.977144	-1.173729	1.63078
32	1	0	4.162849	-2.357542	0.327104
33	1	0	5.933695	-0.280039	0.450842
34	1	0	5.24658	-0.705766	-1.122187
35	1	0	4.511893	1.654921	0.650815
36	1	0	4.462861	1.542494	-1.115972
37	1	0	2.440405	-2.252597	-1.363122

	14*R*-**1b**		Standard Orientation (Ångstroms)		
No.	Atom	Type	X	Y	Z
1	6	0	-4.574507	-1.985139	-0.375301
2	6	0	-5.0085	-0.732252	0.049483
3	6	0	-4.052835	0.284625	0.137253
4	6	0	-2.677125	0.077344	-0.167175
5	6	0	-2.27782	-1.191816	-0.630268

6	6	0	-3.224835	-2.207875	-0.721518
7	7	0	-4.208857	1.619853	0.465547
8	6	0	-3.005115	2.260864	0.371955
9	6	0	-2.011627	1.359023	0.010337
10	7	0	0.406747	-0.304798	0.677943
11	6	0	0.474251	0.96947	0.077407
12	6	0	-0.623111	1.728679	-0.153227
13	6	0	1.431279	-1.188333	0.785066
14	6	0	2.673139	-0.826607	-0.033099
15	7	0	2.844682	0.614247	-0.136384
16	6	0	1.831559	1.518042	-0.190869
17	6	0	4.00005	-1.284508	0.564745
18	6	0	5.01424	-0.334022	-0.095086
19	6	0	4.264331	1.005789	-0.209116
20	8	0	1.35359	-2.231619	1.434878
21	8	0	2.034544	2.718907	-0.424761
22	8	0	2.552321	-1.444821	-1.322914
23	1	0	-5.288564	-2.798645	-0.455236
24	1	0	-6.04992	-0.546817	0.291912
25	1	0	-1.251975	-1.378493	-0.926007
26	1	0	-2.920934	-3.189169	-1.072335
27	1	0	-5.078917	2.060252	0.727117
28	1	0	-2.919679	3.315983	0.588405
29	1	0	-0.449382	-0.553166	1.162734
30	1	0	-0.412079	2.749478	-0.45695
31	1	0	3.968696	-1.138396	1.64801
32	1	0	4.182829	-2.339512	0.359761
33	1	0	5.930983	-0.236081	0.489071
34	1	0	5.28169	-0.70293	-1.088283
35	1	0	4.484054	1.683861	0.621452
36	1	0	4.469142	1.534655	-1.142766
37	1	0	1.832174	-1.025582	-1.817765

	14*R*-**1c**		Standard Orientation (Ångstroms)		
No.	Atom	Type	X	Y	Z
1	6	0	-4.545312	-1.984344	-0.294325
2	6	0	-4.977427	-0.725584	0.114807
3	6	0	-4.023126	0.294661	0.175198
4	6	0	-2.650586	0.084922	-0.141395
5	6	0	-2.253322	-1.190655	-0.588256
6	6	0	-3.199021	-2.210011	-0.652004
7	7	0	-4.177946	1.634731	0.483599
8	6	0	-2.976045	2.276198	0.365723
9	6	0	-1.985456	1.370302	0.00733

10	7	0	0.440228	-0.279282	0.67847
11	6	0	0.499378	0.978415	0.045115
12	6	0	-0.598951	1.736524	-0.182825
13	6	0	1.442154	-1.191904	0.713512
14	6	0	2.636227	-0.831006	-0.181209
15	7	0	2.857441	0.598165	-0.180457
16	6	0	1.855052	1.51549	-0.252035
17	6	0	3.991028	-1.433273	0.246955
18	6	0	4.885979	-0.226077	0.59541
19	6	0	4.285489	0.927238	-0.216557
20	8	0	1.373449	-2.245703	1.348931
21	8	0	2.065282	2.711293	-0.500636
22	8	0	2.262822	-1.205812	-1.523464
23	1	0	-5.258456	-2.800497	-0.35292
24	1	0	-6.016561	-0.538557	0.365754
25	1	0	-1.23036	-1.379073	-0.892713
26	1	0	-2.897019	-3.19636	-0.990155
27	1	0	-5.045913	2.07812	0.746911
28	1	0	-2.890351	3.334925	0.56369
29	1	0	-0.400751	-0.511546	1.195552
30	1	0	-0.391986	2.750827	-0.510287
31	1	0	3.863742	-2.128272	1.077466
32	1	0	4.398267	-1.987152	-0.602808
33	1	0	4.814768	0.004538	1.662605
34	1	0	5.935734	-0.403251	0.35267
35	1	0	4.443682	1.914224	0.218176
36	1	0	4.651035	0.936556	-1.250665
37	1	0	2.257312	-2.173382	-1.585866

Table S14. Gibbs free energies[a] and equilibrium populations[b] of low-energy conformers of 14*S*-**1**

Conformers	In MeOH	
	E	Optical rotation (OR)
14*S*-**1a**	-1008.8250036	–85.15
14*S*-**1b**	-1008.8252284	–80.05
14*S*-**1c**	-1008.8231179	–140.31
Average		–85.56

[a] B3LYP/6-31+G (d, p), in kcal/mol; [b] from Δ*G* values at 298.15K.

Table S15. Cartesian coordinates for the low-energy reoptimized MMFF conformers of 14*S*-**1** at B3LYP/6-311+G (d, p) level of theory in MeOH

14*S*-**1a**			Standard Orientation (Ångstroms)		
No.	Atom	Type	X	Y	Z
1	6	0	4.553043	-1.992839	-0.371113

2	6	0	4.995085	-0.739996	0.045454
3	6	0	4.045127	0.282558	0.130817
4	6	0	2.667384	0.08046	-0.168026
5	6	0	2.259752	-1.188827	-0.623704
6	6	0	3.201114	-2.210344	-0.712242
7	7	0	4.208524	1.618538	0.451598
8	6	0	3.006655	2.264616	0.359593
9	6	0	2.008269	1.365724	0.005789
10	7	0	-0.405816	-0.283507	0.70753
11	6	0	-0.476139	0.979845	0.087066
12	6	0	0.61923	1.736879	-0.157126
13	6	0	-1.417701	-1.180538	0.788408
14	6	0	-2.658104	-0.811281	-0.025811
15	7	0	-2.846907	0.61874	-0.101471
16	6	0	-1.835344	1.521847	-0.184897
17	6	0	-3.98913	-1.302239	0.544187
18	6	0	-5.004295	-0.353571	-0.116424
19	6	0	-4.268776	0.997061	-0.189563
20	8	0	-1.3326	-2.244013	1.406729
21	8	0	-2.042774	2.718631	-0.433808
22	8	0	-2.435651	-1.282977	-1.370886
23	1	0	5.262527	-2.810589	-0.448662
24	1	0	6.038279	-0.558942	0.283595
25	1	0	1.232062	-1.370864	-0.916336
26	1	0	2.891192	-3.191892	-1.057093
27	1	0	5.08148	2.056288	0.707791
28	1	0	2.926972	3.321196	0.571037
29	1	0	0.455308	-0.531659	1.182894
30	1	0	0.408179	2.753075	-0.475819
31	1	0	-4.162849	-2.357542	0.327105
32	1	0	-3.977144	-1.173729	1.630781
33	1	0	-5.24658	-0.705766	-1.122186
34	1	0	-5.933695	-0.280039	0.450842
35	1	0	-4.462861	1.542493	-1.115971
36	1	0	-4.511893	1.654921	0.650815
37	1	0	-2.440405	-2.252598	-1.363121

14*S*-**1b**			Standard Orientation (Ångstroms)		
No.	Atom	Type	X	Y	Z
1	6	0	4.574506	-1.985139	-0.375302
2	6	0	5.0085	-0.732252	0.049483
3	6	0	4.052835	0.284625	0.137253
4	6	0	2.677125	0.077344	-0.167175

5	6	0	2.27782	-1.191815	-0.630269
6	6	0	3.224834	-2.207875	-0.721519
7	7	0	4.208857	1.619853	0.465547
8	6	0	3.005115	2.260864	0.371955
9	6	0	2.011627	1.359023	0.010338
10	7	0	-0.406747	-0.304797	0.677943
11	6	0	-0.474251	0.96947	0.077407
12	6	0	0.623111	1.728679	-0.153227
13	6	0	-1.431279	-1.188333	0.785067
14	6	0	-2.673139	-0.826607	-0.033099
15	7	0	-2.844682	0.614247	-0.136385
16	6	0	-1.831559	1.518042	-0.19087
17	6	0	-4.00005	-1.284507	0.564746
18	6	0	-5.01424	-0.334022	-0.095085
19	6	0	-4.264331	1.005789	-0.209116
20	8	0	-1.35359	-2.231619	1.434878
21	8	0	-2.034544	2.718907	-0.424762
22	8	0	-2.552321	-1.444822	-1.322913
23	1	0	5.288564	-2.798646	-0.455237
24	1	0	6.04992	-0.546818	0.291912
25	1	0	1.251975	-1.378492	-0.926008
26	1	0	2.920934	-3.189169	-1.072337
27	1	0	5.078917	2.060252	0.727118
28	1	0	2.919679	3.315982	0.588406
29	1	0	0.449382	-0.553165	1.162734
30	1	0	0.412079	2.749479	-0.45695
31	1	0	-4.182829	-2.339512	0.359763
32	1	0	-3.968695	-1.138394	1.64801
33	1	0	-5.28169	-0.70293	-1.088282
34	1	0	-5.930983	-0.23608	0.489071
35	1	0	-4.469142	1.534654	-1.142768
36	1	0	-4.484054	1.683862	0.621451
37	1	0	-1.832175	-1.025582	-1.817765

	14*S*-**1c**		Standard Orientation (Ångstroms)		
No.	Atom	Type	X	Y	Z
1	6	0	4.545314	-1.984343	-0.294325
2	6	0	4.977428	-0.725583	0.114807
3	6	0	4.023126	0.294661	0.175198
4	6	0	2.650587	0.084921	-0.141395
5	6	0	2.253324	-1.190655	-0.588256
6	6	0	3.199022	-2.210011	-0.652005
7	7	0	4.177946	1.634731	0.4836
8	6	0	2.976045	2.276198	0.365724

9	6	0	1.985456	1.370302	0.00733
10	7	0	-0.440228	-0.279282	0.678469
11	6	0	-0.499378	0.978415	0.045114
12	6	0	0.598951	1.736523	-0.182825
13	6	0	-1.442155	-1.191905	0.713512
14	6	0	-2.636227	-0.831006	-0.181209
15	7	0	-2.857441	0.598165	-0.180456
16	6	0	-1.855052	1.51549	-0.252035
17	6	0	-3.991028	-1.433273	0.246955
18	6	0	-4.885979	-0.226077	0.59541
19	6	0	-4.28549	0.927238	-0.216555
20	8	0	-1.37345	-2.245703	1.348932
21	8	0	-2.065282	2.711292	-0.500637
22	8	0	-2.262823	-1.205811	-1.523465
23	1	0	5.258459	-2.800495	-0.35292
24	1	0	6.016562	-0.538555	0.365755
25	1	0	1.230361	-1.379074	-0.892713
26	1	0	2.897022	-3.19636	-0.990155
27	1	0	5.045913	2.078121	0.746911
28	1	0	2.89035	3.334925	0.56369
29	1	0	0.40075	-0.511546	1.195552
30	1	0	0.391986	2.750826	-0.510287
31	1	0	-4.398268	-1.987152	-0.602809
32	1	0	-3.863743	-2.128272	1.077466
33	1	0	-5.935734	-0.403251	0.352669
34	1	0	-4.814769	0.004537	1.662605
35	1	0	-4.651036	0.936559	-1.250663
36	1	0	-4.443682	1.914224	0.218179
37	1	0	-2.257311	-2.173381	-1.585867

Table S16. Gibbs free energies[a] and equilibrium populations[b] of low-energy conformers of 5*S*,6*R*,8*S*,9*S*-**21**

Conformers	In MeOH	
	G [a]	*P* (%) [b]
5S,6*R*,8*S*,9*S*-**21a**	-766.2454173	28.51
5S,6*R*,8*S*,9*S*-**21b**	-766.2448880	16.27
5S,6*R*,8*S*,9*S*-**21c**	-766.2431475	2.57
5S,6*R*,8*S*,9*S*-**21d**	-766.2459811	51.83
5S,6*R*,8*S*,9*S*-**21e**	-766.2419866	0.75
5S,6*R*,8*S*,9*S*-**21f**	-766.2398624	0.08

[a] B3LYP/6-31+G (d, p), in kcal/mol; [b] from Δ*G* values at 298.15K.

Table S17. Cartesian coordinates for the low-energy reoptimized MMFF conformers of 5*S*,6*R*,8*S*,9*S*-**21** at B3LYP/6-31+G (d, p) level of theory in MeOH

5S,6*R*,8*S*,9*S*-**21a**			Standard Orientation (Ångstroms)		
Center number	Atomic number	Atomic type	X	Y	Z
1	6	0	-2.782751	-1.890864	-0.912468

2	6	0	-2.465450	-0.830554	0.131897
3	8	0	-1.426666	-1.347208	1.028795
4	6	0	-2.076901	0.522859	-0.452171
5	8	0	-2.263165	1.511757	0.569097
6	6	0	-2.360130	2.847166	0.074711
7	6	0	-0.643483	0.516022	-0.922696
8	6	0	0.275834	-0.277178	-0.350784
9	6	0	1.747279	-0.230649	-0.714781
10	6	0	2.621750	0.563857	0.286124
11	8	0	3.982930	0.460737	-0.144650
12	6	0	2.273845	2.040835	0.370740
13	8	0	2.299226	-1.544976	-0.876844
14	6	0	-0.125912	-1.196808	0.741568
15	8	0	0.689642	-1.819738	1.418295
16	1	0	-1.945263	-2.043916	-1.599625
17	1	0	-3.659224	-1.587182	-1.492825
18	1	0	-3.008809	-2.839759	-0.418772
19	1	0	-3.321162	-0.683047	0.794381
20	1	0	-2.750004	0.735863	-1.293185
21	1	0	-3.163576	2.937898	-0.669283
22	1	0	-2.595079	3.481044	0.933274
23	1	0	-1.420951	3.195443	-0.373054
24	1	0	-0.349847	1.215423	-1.701134
25	1	0	1.845874	0.252299	-1.692528
26	1	0	2.510574	0.100270	1.277825
27	1	0	4.106881	-0.472623	-0.386841
28	1	0	2.989286	2.550653	1.023803
29	1	0	2.318673	2.510309	-0.618434
30	1	0	1.273226	2.187568	0.785482
31	1	0	2.129178	-2.012929	-0.039459

5S,6*R*,8*S*,9*S*-**21b**			Standard Orientation (Ångstroms)		
Center number	Atomic number	Atomic type	X	Y	Z
1	6	0	-3.244650	-1.686384	0.642235
2	6	0	-1.954625	-0.897629	0.565135
3	8	0	-0.961511	-1.781201	-0.046573
4	6	0	-2.048234	0.393711	-0.249590
5	8	0	-2.867503	1.288337	0.495884
6	6	0	-3.519272	2.276403	-0.301545
7	6	0	-0.664474	0.956441	-0.446205
8	6	0	0.423324	0.171485	-0.478312
9	6	0	1.838123	0.718679	-0.579640
10	6	0	2.534353	0.739275	0.803174
11	8	0	2.523917	-0.553425	1.422703
12	6	0	3.948980	1.304798	0.727262
13	8	0	2.615506	0.025786	-1.559861
14	6	0	0.231018	-1.297535	-0.435463
15	8	0	1.081967	-2.108258	-0.795986
16	1	0	-3.103137	-2.592474	1.238109
17	1	0	-3.587368	-1.970466	-0.357773
18	1	0	-4.015684	-1.075255	1.118246
19	1	0	-1.589924	-0.645237	1.567788
20	1	0	-2.510422	0.179736	-1.227102
21	1	0	-4.188440	1.812704	-1.038689
22	1	0	-4.107632	2.892505	0.382526
23	1	0	-2.801955	2.918742	-0.828843
24	1	0	-0.570238	2.035064	-0.552442
25	1	0	1.772252	1.759047	-0.917091

26	1	0	1.921878	1.369787	1.458383
27	1	0	3.046371	-1.148211	0.862824
28	1	0	4.575048	0.693246	0.069612
29	1	0	3.942762	2.328411	0.337072
30	1	0	4.399585	1.318851	1.724260
31	1	0	2.410644	-0.921829	-1.448523

5S,6*R*,8*S*,9*S*-**21c**			Standard Orientation (Ångstroms)		
Center number	Atomic number	Atomic type	X	Y	Z
1	6	0	-2.911417	-1.773608	-0.812369
2	6	0	-2.553079	-0.545940	0.014809
3	8	0	-1.594011	-0.949244	1.049761
4	6	0	-2.040706	0.624847	-0.829901
5	8	0	-2.344511	1.921235	-0.297478
6	6	0	-1.850765	2.215159	1.011474
7	6	0	-0.572932	0.484159	-1.134439
8	6	0	0.251794	-0.273695	-0.394659
9	6	0	1.738024	-0.401825	-0.678284
10	6	0	2.638145	0.488108	0.214936
11	8	0	4.001043	0.156952	-0.066553
12	6	0	2.467218	1.977332	-0.036927
13	8	0	2.176018	-1.764547	-0.578266
14	6	0	-0.276176	-0.973130	0.802661
15	8	0	0.451746	-1.542741	1.613155
16	1	0	-2.066043	-2.105691	-1.422992
17	1	0	-3.749761	-1.539370	-1.475352
18	1	0	-3.211097	-2.592231	-0.152587
19	1	0	-3.422270	-0.214913	0.587645
20	1	0	-2.594607	0.603978	-1.773691
21	1	0	-2.115127	3.257852	1.204006
22	1	0	-2.318942	1.586587	1.778751
23	1	0	-0.761463	2.110637	1.080252
24	1	0	-0.185272	1.055325	-1.974808
25	1	0	1.916520	-0.112610	-1.719216
26	1	0	2.410018	0.259447	1.267200
27	1	0	4.022461	-0.813171	-0.128320
28	1	0	3.182133	2.539227	0.572537
29	1	0	2.653384	2.215648	-1.090165
30	1	0	1.460996	2.313477	0.226751
31	1	0	1.916156	-2.065876	0.311225

5S,6*R*,8*S*,9*S*-**21d**			Standard Orientation (Ångstroms)		
Center number	Atomic number	Atomic type	X	Y	Z
1	6	0	2.581328	-1.932387	1.120624
2	6	0	2.458834	-0.929298	-0.015962
3	8	0	1.393912	-1.358046	-0.928198
4	6	0	2.231184	0.510265	0.426836
5	8	0	2.535139	1.348772	-0.693467
6	6	0	2.593658	2.739188	-0.380387
7	6	0	0.804688	0.687409	0.889001
8	6	0	-0.198020	-0.021408	0.345537
9	6	0	-1.637715	0.145210	0.813775
10	6	0	-2.619706	0.728671	-0.245097
11	8	0	-3.151180	-0.329875	-1.049067
12	6	0	-3.801687	1.423101	0.417448
13	8	0	-2.155280	-1.088010	1.313562
14	6	0	0.120883	-0.984522	-0.740220
15	8	0	-0.715586	-1.451516	-1.511750
16	1	0	1.698671	-1.923613	1.766351

17	1	0	3.460815	-1.696342	1.727453
18	1	0	2.706202	-2.938813	0.712174
19	1	0	3.352233	-0.957365	-0.644057
20	1	0	2.921501	0.734206	1.251542
21	1	0	2.950222	3.246830	-1.279998
22	1	0	1.609855	3.145943	-0.113326
23	1	0	3.294671	2.934084	0.443083
24	1	0	0.592981	1.422071	1.661706
25	1	0	-1.599488	0.847163	1.653665
26	1	0	-2.073796	1.446087	-0.873104
27	1	0	-2.381985	-0.767965	-1.469601
28	1	0	-4.304579	0.742937	1.113084
29	1	0	-3.470714	2.306767	0.971232
30	1	0	-4.523851	1.740662	-0.340897
31	1	0	-2.689453	-1.450629	0.582715

5S,6*R*,8*S*,9*S*-**21e**			Standard Orientation (Ångstroms)		
Center number	Atomic number	Atomic type	X	Y	Z
1	6	0	-3.453218	-1.319125	0.621836
2	6	0	-2.113094	-0.633502	0.464496
3	8	0	-1.121915	-1.692264	0.250897
4	6	0	-2.043114	0.347268	-0.713098
5	8	0	-2.945414	1.439780	-0.579605
6	6	0	-2.723327	2.314055	0.532385
7	6	0	-0.615321	0.783693	-0.915402
8	6	0	0.419922	-0.010474	-0.601344
9	6	0	1.869991	0.423005	-0.750830
10	6	0	2.463078	0.875702	0.606327
11	8	0	2.321924	-0.136088	1.611932
12	6	0	3.913469	1.329977	0.477317
13	8	0	2.674111	-0.578939	-1.378421
14	6	0	0.132180	-1.384558	-0.122388
15	8	0	0.964218	-2.289703	-0.113765
16	1	0	-3.712734	-1.875933	-0.284223
17	1	0	-4.228659	-0.569914	0.803167
18	1	0	-3.434215	-2.010134	1.469279
19	1	0	-1.824890	-0.123142	1.389629
20	1	0	-2.380938	-0.175028	-1.619610
21	1	0	-2.997629	1.848826	1.486419
22	1	0	-1.681784	2.653333	0.587834
23	1	0	-3.367167	3.181339	0.368086
24	1	0	-0.449469	1.761964	-1.362306
25	1	0	1.892432	1.295559	-1.413271
26	1	0	1.846845	1.710783	0.959721
27	1	0	2.847413	-0.901123	1.331160
28	1	0	4.290065	1.658198	1.450817
29	1	0	4.544452	0.511307	0.116718
30	1	0	4.002882	2.164680	-0.226470
31	1	0	2.406594	-1.429249	-0.981047

5S,6*R*,8*S*,9*S*-**21f**			Standard Orientation (Ångstroms)		
Center number	Atomic number	Atomic type	X	Y	Z
1	6	0	-2.998164	-1.767439	-0.621978
2	6	0	-2.556716	-0.494322	0.087734
3	8	0	-1.540096	-0.849768	1.084537
4	6	0	-2.073010	0.598010	-0.869063
5	8	0	-2.282108	1.937725	-0.398806
6	6	0	-1.670402	2.291696	0.845017
7	6	0	-0.636862	0.386837	-1.263482

8	6	0	0.225271	-0.312721	-0.510114
9	6	0	1.705772	-0.421991	-0.854716
10	6	0	2.509952	0.763248	-0.257246
11	8	0	2.192942	1.008428	1.120027
12	6	0	4.012840	0.596964	-0.457213
13	8	0	2.246343	-1.697073	-0.509100
14	6	0	-0.238017	-0.899937	0.770836
15	8	0	0.524727	-1.406991	1.593608
16	1	0	-3.272235	-2.527323	0.114721
17	1	0	-2.203768	-2.166562	-1.260044
18	1	0	-3.873606	-1.558168	-1.244076
19	1	0	-3.377194	-0.098817	0.691007
20	1	0	-2.700460	0.543354	-1.763916
21	1	0	-0.586450	2.126856	0.842768
22	1	0	-1.862032	3.359269	0.978666
23	1	0	-2.112545	1.747182	1.688157
24	1	0	-0.293986	0.882061	-2.169356
25	1	0	1.803769	-0.344884	-1.942726
26	1	0	2.168343	1.663062	-0.782017
27	1	0	2.332444	0.184611	1.612847
28	1	0	4.538121	1.475812	-0.071273
29	1	0	4.379610	-0.289096	0.069837
30	1	0	4.258469	0.487108	-1.519254
31	1	0	1.983078	-1.865096	0.413875

Table S18. Gibbs free energies[a] and equilibrium populations[b] of low-energy conformers of 5*S*,6*R*,8*R*,9*R*-**21**

Conformers	In MeOH	
	G [a]	*P* (%) [b]
5*S*,6*R*,8*R*,9*R*-**21a**	-766.2463708	82.62
5*S*,6*R*,8*R*,9*R*-**21b**	-766.2444642	10.95
5*S*,6*R*,8*R*,9*R*-**21c**	-766.2409553	0.27
5*S*,6*R*,8*R*,9*R*-**21d**	-766.2432615	3.06
5*S*,6*R*,8*R*,9*R*-**21e**	-766.2431714	2.78
5*S*,6*R*,8*R*,9*R*-**21f**	-766.2411516	0.33

[a] B3LYP/6-31+G (d, p), in kcal/mol; [b] from Δ*G* values at 298.15K.

Table S19. Cartesian coordinates for the low-energy reoptimized MMFF conformers of 5*S*,6*R*,8*R*,9*R*-**21** at B3LYP/6-31+G (d, p) level of theory in MeOH

5*S*,6*R*,8*R*,9*R*-**21a**			Standard Orientation (Ångstroms)		
Center number	Atomic number	Atomic type	X	Y	Z
1	6	0	1.730369	2.445923	-0.725876
2	6	0	2.116881	1.241133	0.118354
3	8	0	1.236205	1.172320	1.289530
4	6	0	2.128307	-0.082748	-0.637679
5	8	0	2.923378	-1.000842	0.121949
6	6	0	3.274198	-2.190316	-0.583909
7	6	0	0.725094	-0.607737	-0.823316
8	6	0	-0.248915	-0.315374	0.051991
9	6	0	-1.663996	-0.845186	-0.073093
10	6	0	-2.710486	0.235815	-0.436005
11	8	0	-4.004840	-0.370172	-0.382784
12	6	0	-2.527704	0.815879	-1.829046
13	8	0	-2.082113	-1.518505	1.124172
14	6	0	0.065721	0.523039	1.236303
15	8	0	-0.690897	0.620095	2.201141
16	1	0	2.472165	2.595190	-1.516315

17	1	0	1.708255	3.342835	-0.101127
18	1	0	0.747983	2.317613	-1.189650
19	1	0	3.105920	1.389624	0.557458
20	1	0	2.597739	0.084062	-1.616955
21	1	0	2.403824	-2.829251	-0.779700
22	1	0	3.766342	-1.956508	-1.538133
23	1	0	3.973301	-2.736557	0.054016
24	1	0	0.522392	-1.266751	-1.663470
25	1	0	-1.682452	-1.603659	-0.862928
26	1	0	-2.647043	1.040558	0.313224
27	1	0	-3.994916	-0.922629	0.417567
28	1	0	-2.542899	0.022130	-2.584304
29	1	0	-3.340870	1.514580	-2.049745
30	1	0	-1.582870	1.359904	-1.911964
31	1	0	-1.897545	-0.896886	1.853609
5*S*,6*R*,8*R*,9*R*-**21b**			Standard Orientation (Ångstroms)		
Center number	Atomic number	Atomic type	X	Y	Z
1	6	0	1.909820	2.435210	0.529463
2	6	0	2.234222	0.949847	0.445836
3	8	0	1.442203	0.242672	1.459057
4	6	0	2.045314	0.362622	-0.957639
5	8	0	2.945735	-0.706275	-1.278248
6	6	0	2.874478	-1.877255	-0.460066
7	6	0	0.615210	-0.032476	-1.208410
8	6	0	-0.259400	-0.258382	-0.215525
9	6	0	-1.703772	-0.650241	-0.459837
10	6	0	-2.722234	0.457705	-0.097167
11	8	0	-4.035973	-0.089102	-0.240105
12	6	0	-2.631449	1.683530	-0.991369
13	8	0	-2.052302	-1.853934	0.239881
14	6	0	0.203774	-0.195248	1.192892
15	8	0	-0.478945	-0.594819	2.134684
16	1	0	2.610397	3.000842	-0.092201
17	1	0	2.008260	2.780069	1.562246
18	1	0	0.892130	2.643532	0.185253
19	1	0	3.264877	0.774845	0.762853
20	1	0	2.320412	1.146586	-1.670457
21	1	0	3.163938	-1.672415	0.577710
22	1	0	1.876682	-2.331731	-0.470762
23	1	0	3.586912	-2.586021	-0.889114
24	1	0	0.312900	-0.166745	-2.244474
25	1	0	-1.825605	-0.879233	-1.523826
26	1	0	-2.553878	0.748732	0.951482
27	1	0	-3.983145	-0.982688	0.140230
28	1	0	-2.750953	1.404677	-2.044301
29	1	0	-3.425267	2.391192	-0.732135
30	1	0	-1.671578	2.193082	-0.870963
31	1	0	-1.787609	-1.704937	1.167235
5*S*,6*R*,8*R*,9*R*-**21c**			Standard Orientation (Ångstroms)		
Center number	Atomic number	Atomic type	X	Y	Z
1	6	0	3.086982	-1.564286	0.873594
2	6	0	2.601242	-0.430014	-0.020100
3	8	0	1.713902	-0.994391	-1.040268
4	6	0	1.951199	0.716987	0.762964
5	8	0	2.137545	2.011529	0.171761
6	6	0	1.519940	2.235742	-1.098969
7	6	0	0.499610	0.446368	1.043796

8	6	0	-0.223739	-0.424755	0.324729
9	6	0	-1.707991	-0.658291	0.547393
10	6	0	-2.572964	0.121840	-0.471622
11	8	0	-2.351046	1.530596	-0.344546
12	6	0	-4.056438	-0.217800	-0.345331
13	8	0	-2.122009	-0.217982	1.838435
14	6	0	0.394265	-1.141168	-0.815171
15	8	0	-0.250989	-1.824882	-1.600942
16	1	0	3.884353	-1.203460	1.530399
17	1	0	3.485832	-2.377090	0.260662
18	1	0	2.276064	-1.958312	1.494352
19	1	0	3.435383	-0.027571	-0.600041
20	1	0	2.489105	0.797876	1.713041
21	1	0	1.772687	3.262091	-1.376736
22	1	0	1.905541	1.558132	-1.870421
23	1	0	0.428634	2.142306	-1.050355
24	1	0	0.026043	1.019521	1.835547
25	1	0	-1.918427	-1.727625	0.414750
26	1	0	-2.223008	-0.140695	-1.473448
27	1	0	-2.573291	1.770282	0.568907
28	1	0	-4.630295	0.347604	-1.085821
29	1	0	-4.227757	-1.285513	-0.519609
30	1	0	-4.433774	0.032081	0.651276
31	1	0	-1.668956	-0.756329	2.504279

5*S*,6*R*,8*R*,9*R*-**21d**			Standard Orientation (Ångstroms)		
Center number	Atomic number	Atomic type	X	Y	Z
1	6	0	1.506688	2.288763	-1.183311
2	6	0	2.014299	1.299994	-0.144481
3	8	0	1.139996	1.351080	1.031518
4	6	0	2.159697	-0.132893	-0.643927
5	8	0	3.011721	-0.819896	0.279934
6	6	0	3.456620	-2.095290	-0.178845
7	6	0	0.805839	-0.792926	-0.747414
8	6	0	-0.207059	-0.430946	0.054069
9	6	0	-1.601148	-1.026986	-0.065279
10	6	0	-2.528809	-0.098608	-0.889399
11	8	0	-2.517223	1.247103	-0.393224
12	6	0	-3.950598	-0.641194	-0.985321
13	8	0	-2.158784	-1.359624	1.207112
14	6	0	0.029864	0.603620	1.093654
15	8	0	-0.721363	0.781780	2.052224
16	1	0	2.208568	2.340884	-2.021158
17	1	0	1.430122	3.283813	-0.736747
18	1	0	0.522733	2.002934	-1.565986
19	1	0	2.981095	1.624213	0.247028
20	1	0	2.637219	-0.107390	-1.633212
21	1	0	4.182057	-2.455580	0.554655
22	1	0	2.636150	-2.820897	-0.247784
23	1	0	3.946365	-2.017928	-1.159577
24	1	0	0.666413	-1.586718	-1.476953
25	1	0	-1.520600	-1.967435	-0.621793
26	1	0	-2.093791	-0.028671	-1.893255
27	1	0	-2.872185	1.230828	0.509374
28	1	0	-3.961113	-1.636828	-1.442145
29	1	0	-4.403754	-0.718062	0.008010
30	1	0	-4.563892	0.025890	-1.598573
31	1	0	-1.942520	-0.613804	1.799130

5*S*,6*R*,8*R*,9*R*-**21e**			Standard Orientation (Ångstroms)		
Center number	Atomic number	Atomic type	X	Y	Z
1	6	0	1.562947	2.201548	-0.998624
2	6	0	2.013079	1.092817	-0.057305
3	8	0	1.147190	1.089560	1.125649
4	6	0	2.061012	-0.297653	-0.693693
5	8	0	2.814903	-1.226913	0.093700
6	6	0	4.216947	-1.208181	-0.175982
7	6	0	0.677418	-0.871457	-0.824916
8	6	0	-0.312428	-0.510924	0.004846
9	6	0	-1.738121	-1.017944	-0.148277
10	6	0	-2.610520	0.025181	-0.892835
11	8	0	-2.489240	1.334860	-0.319649
12	6	0	-4.070340	-0.404489	-0.990781
13	8	0	-2.308849	-1.419393	1.097951
14	6	0	-0.015167	0.424022	1.119890
15	8	0	-0.763263	0.579720	2.085252
16	1	0	2.262434	2.283443	-1.836297
17	1	0	1.550896	3.156817	-0.466702
18	1	0	0.561345	2.012276	-1.395420
19	1	0	2.995116	1.332966	0.355840
20	1	0	2.515152	-0.200726	-1.688696
21	1	0	4.672805	-1.947416	0.487247
22	1	0	4.424916	-1.482856	-1.219046
23	1	0	4.665594	-0.227249	0.028130
24	1	0	0.506689	-1.616796	-1.597741
25	1	0	-1.713978	-1.914858	-0.777161
26	1	0	-2.189063	0.124180	-1.900038
27	1	0	-2.792274	1.284878	0.600387
28	1	0	-4.162496	-1.367568	-1.504794
29	1	0	-4.512583	-0.506952	0.004986
30	1	0	-4.641231	0.341440	-1.551966
31	1	0	-2.058226	-0.731763	1.744108

5*S*,6*R*,8*R*,9*R*-**21f**			Standard Orientation (Ångstroms)		
Center number	Atomic number	Atomic type	X	Y	Z
1	6	0	1.669925	2.484804	-0.421293
2	6	0	2.141556	1.115805	0.051204
3	8	0	1.363515	0.743800	1.237627
4	6	0	2.081296	0.036785	-1.035016
5	8	0	3.062595	-0.999221	-0.898530
6	6	0	3.002722	-1.793740	0.288753
7	6	0	0.690273	-0.520800	-1.170088
8	6	0	-0.226068	-0.425868	-0.194119
9	6	0	-1.662326	-0.892213	-0.379143
10	6	0	-2.579790	0.299793	-0.754757
11	8	0	-2.438533	1.393926	0.161504
12	6	0	-4.039409	-0.117409	-0.898317
13	8	0	-2.150090	-1.623015	0.746446
14	6	0	0.167446	0.150606	1.116375
15	8	0	-0.517751	0.043359	2.133141
16	1	0	2.320849	2.846511	-1.222884
17	1	0	1.713927	3.197161	0.407002
18	1	0	0.641592	2.446111	-0.793678
19	1	0	3.165063	1.179894	0.427630
20	1	0	2.353860	0.519604	-1.979165
21	1	0	2.028826	-2.282220	0.413065
22	1	0	3.768500	-2.564667	0.172720

23	1	0	3.226947	-1.207547	1.188095
24	1	0	0.445024	-1.025597	-2.102002
25	1	0	-1.683778	-1.585192	-1.227715
26	1	0	-2.213164	0.689057	-1.711754
27	1	0	-2.723575	1.083456	1.034922
28	1	0	-4.152413	-0.897255	-1.659340
29	1	0	-4.426156	-0.507150	0.048524
30	1	0	-4.645536	0.743952	-1.194880
31	1	0	-1.851590	-1.133476	1.536354

Table S20. Gibbs free energies[a] and equilibrium populations[b] of low-energy conformers of 5*S*,6*R*,8*S*,9*R*-**21**

Conformers	In MeOH	
	G [a]	*P* (%) [b]
5*S*,6*R*,8*S*,9*R*-21**a**	-766.2448846	25.72
5*S*,6*R*,8*S*,9*R*-21**b**	-766.2458611	72.4
5*S*,6*R*,8*S*,9*R*-21**c**	-766.2420743	1.31
5*S*,6*R*,8*S*,9*R*-21**d**	-766.2405191	0.25
5*S*,6*R*,8*S*,9*R*-21**e**	-766.2407526	0.32

[a] B3LYP/6-31+G (d, p), in kcal/mol; [b] from Δ*G* values at 298.15K.

Table S21. Cartesian coordinates for the low-energy reoptimized MMFF conformers of 5*S*,6*R*,8*S*,9*R*-**21** at B3LYP/6-31+G (d, p) level of theory in MeOH

5*S*,6*R*,8*S*,9*R*-**21a**			Standard Orientation (Ångstroms)		
Center number	Atomic number	Atomic type	X	Y	Z
1	6	0	3.323383	1.546963	0.768286
2	6	0	1.995469	0.829755	0.646728
3	8	0	1.024887	1.826057	0.190426
4	6	0	2.002323	-0.355504	-0.320795
5	8	0	2.795256	-1.371475	0.284753
6	6	0	3.373038	-2.282528	-0.649348
7	6	0	0.589320	-0.826210	-0.547111
8	6	0	-0.458426	0.005979	-0.451538
9	6	0	-1.901370	-0.423952	-0.645278
10	6	0	-2.713708	-0.366774	0.672710
11	8	0	-2.055531	-1.130053	1.690144
12	6	0	-4.154105	-0.826391	0.476791
13	8	0	-2.537848	0.336033	-1.676519
14	6	0	-0.203537	1.443897	-0.203025
15	8	0	-1.047699	2.318453	-0.381158
16	1	0	3.644441	1.937833	-0.202438
17	1	0	4.081045	0.849830	1.135030
18	1	0	3.245289	2.377477	1.475562
19	1	0	1.653611	0.470817	1.624645
20	1	0	2.450307	-0.046071	-1.279272
21	1	0	4.049837	-1.764349	-1.341993
22	1	0	3.942137	-3.010467	-0.066309
23	1	0	2.609571	-2.814330	-1.231785
24	1	0	0.441087	-1.874692	-0.796588
25	1	0	-1.900101	-1.464616	-0.990894
26	1	0	-2.703789	0.666261	1.039334
27	1	0	-2.099906	-2.062958	1.428248
28	1	0	-4.182727	-1.865702	0.125713
29	1	0	-4.698212	-0.768253	1.424639
30	1	0	-4.669910	-0.204156	-0.259239
31	1	0	-2.409527	1.269844	-1.430340

5*S*,6*R*,8*S*,9*R*-**21b**			Standard Orientation (Ångstroms)		
Center number	Atomic number	Atomic type	X	Y	Z
1	6	0	-2.339634	-2.237050	-0.539242
2	6	0	-2.202527	-0.948108	0.258878
3	8	0	-1.079429	-1.075016	1.192140
4	6	0	-2.048235	0.308516	-0.598014
5	8	0	-2.271246	1.507927	0.153298
6	6	0	-3.641335	1.906365	0.213327
7	6	0	-0.650926	0.403903	-1.144233
8	6	0	0.402291	-0.101183	-0.482424
9	6	0	1.812458	-0.019260	-1.055061
10	6	0	2.854015	0.811012	-0.256954
11	8	0	3.444263	0.012171	0.775192
12	6	0	2.314201	2.132504	0.275892
13	8	0	2.333861	-1.330981	-1.276774
14	6	0	0.169501	-0.749126	0.833670
15	8	0	1.059821	-0.976081	1.651878
16	1	0	-2.396869	-3.091062	0.141134
17	1	0	-1.494114	-2.385112	-1.217193
18	1	0	-3.259632	-2.207205	-1.131160
19	1	0	-3.062027	-0.830102	0.922751
20	1	0	-2.767154	0.248964	-1.425307
21	1	0	-4.037995	2.118538	-0.788759
22	1	0	-4.273117	1.146571	0.691280
23	1	0	-3.675038	2.819241	0.813021
24	1	0	-0.505483	0.937926	-2.079920
25	1	0	1.708618	0.460449	-2.034460
26	1	0	3.674291	1.009947	-0.956020
27	1	0	2.705208	-0.286598	1.345374
28	1	0	3.123964	2.703219	0.740677
29	1	0	1.890022	2.737069	-0.533282
30	1	0	1.533994	1.970509	1.026992
31	1	0	2.918313	-1.501951	-0.516040

5*S*,6*R*,8*S*,9*R*-**21c**			Standard Orientation (Ångstroms)		
Center number	Atomic number	Atomic type	X	Y	Z
1	6	0	3.523997	-1.194925	-0.677736
2	6	0	2.148662	-0.586691	-0.506073
3	8	0	1.196852	-1.702166	-0.472607
4	6	0	1.988962	0.248637	0.770605
5	8	0	2.820484	1.404839	0.777828
6	6	0	2.554808	2.380239	-0.237095
7	6	0	0.535178	0.577845	0.985173
8	6	0	-0.448655	-0.213535	0.532588
9	6	0	-1.924463	0.093461	0.712666
10	6	0	-2.584955	0.530276	-0.620696
11	8	0	-1.888311	1.659804	-1.161841
12	6	0	-4.072390	0.821235	-0.457801
13	8	0	-2.624910	-0.999478	1.310298
14	6	0	-0.088507	-1.482681	-0.142810
15	8	0	-0.899666	-2.377953	-0.367885
16	1	0	4.271666	-0.398776	-0.730272
17	1	0	3.573902	-1.778858	-1.601041
18	1	0	3.768128	-1.846236	0.167592
19	1	0	1.878842	0.018576	-1.377953
20	1	0	2.341691	-0.345996	1.625385
21	1	0	3.077837	3.289189	0.069779
22	1	0	2.936330	2.069644	-1.217060

23	1	0	1.483878	2.600171	-0.326769
24	1	0	0.306996	1.473367	1.559669
25	1	0	-2.012832	0.931819	1.414044
26	1	0	-2.445735	-0.271233	-1.355759
27	1	0	-2.066136	2.416672	-0.581574
28	1	0	-4.499825	1.130014	-1.416866
29	1	0	-4.611139	-0.061885	-0.105088
30	1	0	-4.230578	1.629850	0.267037
31	1	0	-2.404437	-1.779842	0.769606

5*S*,6*R*,8*S*,9*R*-**21d**			Standard Orientation (Ångstroms)		
Center number	Atomic number	Atomic type	X	Y	Z
1	6	0	-3.026940	-1.703162	-0.725747
2	6	0	-2.566396	-0.486767	0.066587
3	8	0	-1.592585	-0.934358	1.068800
4	6	0	-2.028731	0.642741	-0.819387
5	8	0	-2.268549	1.960168	-0.304981
6	6	0	-1.639258	2.293179	0.936202
7	6	0	-0.576261	0.444513	-1.155780
8	6	0	0.239809	-0.326727	-0.421783
9	6	0	1.728540	-0.457767	-0.702554
10	6	0	2.585662	0.444132	0.217394
11	8	0	2.117693	1.784514	0.008900
12	6	0	4.072328	0.331003	-0.100563
13	8	0	2.160617	-1.815650	-0.631811
14	6	0	-0.283430	-1.010157	0.784671
15	8	0	0.441710	-1.611161	1.575157
16	1	0	-3.877930	-1.431431	-1.357641
17	1	0	-3.343321	-2.495767	-0.042417
18	1	0	-2.226993	-2.089031	-1.365482
19	1	0	-3.390890	-0.097287	0.668345
20	1	0	-2.611877	0.625128	-1.745571
21	1	0	-1.931227	3.324119	1.151170
22	1	0	-1.983171	1.652462	1.757319
23	1	0	-0.546019	2.242037	0.872857
24	1	0	-0.183559	1.006801	-1.999730
25	1	0	1.905138	-0.134571	-1.734314
26	1	0	2.408045	0.144063	1.259624
27	1	0	2.612823	2.362276	0.607754
28	1	0	4.648913	0.989630	0.558516
29	1	0	4.429634	-0.691716	0.046092
30	1	0	4.267100	0.625153	-1.138186
31	1	0	1.920301	-2.125891	0.258916

5*S*,6*R*,8*S*,9*R*-**21e**			Standard Orientation (Ångstroms)		
Center number	Atomic number	Atomic type	X	Y	Z
1	6	0	-3.015861	-1.717088	-0.723257
2	6	0	-2.560036	-0.499231	0.069751
3	8	0	-1.581562	-0.943929	1.068515
4	6	0	-2.032104	0.635054	-0.817026
5	8	0	-2.292108	1.950760	-0.308941
6	6	0	-1.668012	2.298101	0.930883
7	6	0	-0.576453	0.453123	-1.148980
8	6	0	0.244850	-0.319319	-0.422076
9	6	0	1.732594	-0.441281	-0.704274
10	6	0	2.589488	0.447455	0.236984
11	8	0	2.138364	1.806059	0.193391
12	6	0	4.075840	0.349508	-0.088678
13	8	0	2.166599	-1.801735	-0.662375

14	6	0	-0.273180	-1.015474	0.779555
15	8	0	0.456123	-1.621294	1.562161
16	1	0	-3.870350	-1.449585	-1.352230
17	1	0	-3.325213	-2.512832	-0.040343
18	1	0	-2.215367	-2.096974	-1.365976
19	1	0	-3.385123	-0.115361	0.674254
20	1	0	-2.610707	0.606723	-1.745912
21	1	0	-1.975640	3.325189	1.142358
22	1	0	-2.001799	1.654845	1.754248
23	1	0	-0.574294	2.262707	0.867371
24	1	0	-0.188599	1.022223	-1.990805
25	1	0	1.907978	-0.100506	-1.731623
26	1	0	2.414906	0.123069	1.268431
27	1	0	2.332251	2.154108	-0.691013
28	1	0	4.650466	0.981290	0.595720
29	1	0	4.433577	-0.679024	0.005186
30	1	0	4.269546	0.686483	-1.114779
31	1	0	1.936284	-2.126317	0.225802

Table S22. Gibbs free energies[a] and equilibrium populations[b] of low-energy conformers of 5*S*,6*R*,8*R*,9*S*-**21**

Conformers	In MeOH	
	G [a]	*P* (%) [b]
5*S*,6*R*,8*R*,9*S*-**21a**	-766.2434453	16.48
5*S*,6*R*,8*R*,9*S*-**21b**	-766.2439478	28.08
5*S*,6*R*,8*R*,9*S*-**21c**	-766.2435173	17.79
5*S*,6*R*,8*R*,9*S*-**21d**	-766.2434690	16.90
5*S*,6*R*,8*R*,9*S*-**21e**	-766.2435679	18.77
5*S*,6*R*,8*R*,9*S*-**21f**	-766.2414446	1.98

[a] B3LYP/6-31+G (d, p), in kcal/mol; [b] from Δ*G* values at 298.15K.

Table S23. Cartesian coordinates for the low-energy reoptimized MMFF conformers of 5*S*,6*R*,8*R*,9*S*-**21** at B3LYP/6-31+G (d, p) level of theory in MeOH

5*S*,6*R*,8*R*,9*S*-**21a**			Standard Orientation (Ångstroms)		
Center number	Atomic number	Atomic type	X	Y	Z
1	6	0	1.497190	2.491048	-0.602159
2	6	0	2.028830	1.284646	0.158201
3	8	0	1.208892	1.072506	1.356854
4	6	0	2.114826	0.003783	-0.664702
5	8	0	2.968960	-0.899530	0.045811
6	6	0	3.344449	-2.054002	-0.703110
7	6	0	0.734808	-0.574672	-0.871106
8	6	0	-0.233049	-0.384523	0.037036
9	6	0	-1.650022	-0.901887	-0.129295
10	6	0	-2.658328	0.245471	-0.367670
11	8	0	-2.157855	0.991151	-1.487087
12	6	0	-4.063187	-0.279084	-0.638305
13	8	0	-2.054375	-1.707251	0.977909
14	6	0	0.074806	0.360864	1.283103
15	8	0	-0.653672	0.330364	2.273164
16	1	0	2.169830	2.732484	-1.430842
17	1	0	1.451291	3.355628	0.065600
18	1	0	0.497108	2.306023	-1.005082
19	1	0	3.019383	1.497649	0.566164
20	1	0	2.565688	0.246903	-1.636983
21	1	0	2.493297	-2.720551	-0.892121
22	1	0	3.798618	-1.775891	-1.664389

23	1	0	4.082655	-2.590140	-0.101666
24	1	0	0.537419	-1.159926	-1.765414
25	1	0	-1.669935	-1.552969	-1.010215
26	1	0	-2.671151	0.882102	0.529221
27	1	0	-2.773388	1.721477	-1.647087
28	1	0	-4.427674	-0.870763	0.205923
29	1	0	-4.756501	0.555203	-0.792857
30	1	0	-4.072302	-0.906742	-1.536668
31	1	0	-1.881866	-1.168282	1.771267
5*S*,6*R*,8*R*,9*S*-**21b**			Standard Orientation (Ångstroms)		
Center number	Atomic number	Atomic type	X	Y	Z
1	6	0	-3.514271	-1.455112	-0.480161
2	6	0	-2.227639	-0.965438	0.150336
3	8	0	-1.154620	-1.803283	-0.386252
4	6	0	-1.916006	0.508148	-0.114709
5	8	0	-2.867025	1.262036	0.631324
6	6	0	-3.113741	2.564005	0.101801
7	6	0	-0.508388	0.813478	0.323432
8	6	0	0.454130	-0.121377	0.326346
9	6	0	1.898426	0.162450	0.704636
10	6	0	2.771386	0.507133	-0.532561
11	8	0	2.221794	1.635938	-1.220675
12	6	0	4.230177	0.744616	-0.157706
13	8	0	2.465386	-0.906711	1.462490
14	6	0	0.134219	-1.474515	-0.179543
15	8	0	0.990424	-2.311833	-0.452991
16	1	0	-4.354099	-0.875205	-0.088986
17	1	0	-3.679656	-2.509470	-0.241240
18	1	0	-3.482496	-1.339431	-1.568161
19	1	0	-2.242280	-1.119509	1.235913
20	1	0	-2.024043	0.721736	-1.190814
21	1	0	-2.206406	3.181690	0.088120
22	1	0	-3.518871	2.507096	-0.917480
23	1	0	-3.850688	3.034401	0.757070
24	1	0	-0.288054	1.833327	0.630636
25	1	0	1.908619	1.037978	1.365046
26	1	0	2.704314	-0.323553	-1.244466
27	1	0	2.362308	2.415307	-0.660414
28	1	0	4.316156	1.575968	0.553464
29	1	0	4.671117	-0.143111	0.302537
30	1	0	4.807806	0.996036	-1.052697
31	1	0	2.378843	-1.702772	0.909077
5*S*,6*R*,8*R*,9*S*-**21c**			Standard Orientation (Ångstroms)		
Center number	Atomic number	Atomic type	X	Y	Z
1	6	0	1.506053	2.487866	-0.618524
2	6	0	2.031534	1.284468	0.150755
3	8	0	1.207389	1.080476	1.348060
4	6	0	2.116830	-0.001338	-0.664418
5	8	0	2.963675	-0.903335	0.056156
6	6	0	3.334330	-2.066742	-0.681103
7	6	0	0.735236	-0.575396	-0.873896
8	6	0	-0.234290	-0.380289	0.031485
9	6	0	-1.650889	-0.897304	-0.131797
10	6	0	-2.664843	0.254742	-0.355388
11	8	0	-2.215889	1.124700	-1.402093
12	6	0	-4.068231	-0.271037	-0.633407
13	8	0	-2.045737	-1.716882	0.970605

14	6	0	0.073702	0.368156	1.276210
15	8	0	-0.655334	0.339344	2.265672
16	1	0	1.460751	3.356772	0.043629
17	1	0	0.506650	2.303833	-1.023363
18	1	0	2.182433	2.721926	-1.446351
19	1	0	3.021297	1.497208	0.560735
20	1	0	2.573654	0.234225	-1.635760
21	1	0	3.793514	-1.800267	-1.643319
22	1	0	4.067125	-2.602096	-0.072349
23	1	0	2.479596	-2.729663	-0.866888
24	1	0	0.539482	-1.164219	-1.766381
25	1	0	-1.673048	-1.543685	-1.017417
26	1	0	-2.676218	0.878276	0.545740
27	1	0	-2.237168	0.622434	-2.231710
28	1	0	-4.420615	-0.899340	0.189125
29	1	0	-4.765596	0.563182	-0.757722
30	1	0	-4.079928	-0.871495	-1.551727
31	1	0	-1.881347	-1.180251	1.767066

5*S*,6*R*,8*R*,9*S*-**21d**			Standard Orientation (Ångstroms)		
Center number	Atomic number	Atomic type	X	Y	Z
1	6	0	1.554152	2.401995	0.203251
2	6	0	2.023108	0.980063	0.483069
3	8	0	1.204665	0.407794	1.557741
4	6	0	2.016357	0.065467	-0.744208
5	8	0	2.763648	-1.136801	-0.528500
6	6	0	4.156492	-1.009894	-0.815099
7	6	0	0.611910	-0.354351	-1.081233
8	6	0	-0.336347	-0.428775	-0.136775
9	6	0	-1.777577	-0.804150	-0.427797
10	6	0	-2.731772	0.398663	-0.244468
11	8	0	-2.198445	1.460617	-1.048795
12	6	0	-4.159809	0.060999	-0.655174
13	8	0	-2.208564	-1.913720	0.360556
14	6	0	0.022486	-0.158792	1.277361
15	8	0	-0.704179	-0.474695	2.218108
16	1	0	1.559419	2.983493	1.129305
17	1	0	0.543198	2.418262	-0.214912
18	1	0	2.233760	2.882511	-0.507352
19	1	0	3.025080	0.996190	0.917180
20	1	0	2.445883	0.623900	-1.586512
21	1	0	4.609708	-1.981166	-0.601999
22	1	0	4.323661	-0.758342	-1.871262
23	1	0	4.641245	-0.249622	-0.189049
24	1	0	0.390515	-0.625304	-2.110345
25	1	0	-1.838680	-1.124262	-1.473750
26	1	0	-2.714062	0.688800	0.816307
27	1	0	-2.782090	2.226033	-0.941252
28	1	0	-4.198839	-0.218508	-1.714141
29	1	0	-4.551741	-0.767987	-0.059377
30	1	0	-4.813460	0.927020	-0.502204
31	1	0	-2.004586	-1.673926	1.282913

5*S*,6*R*,8*R*,9*S*-**21e**			Standard Orientation (Ångstroms)		
Center number	Atomic number	Atomic type	X	Y	Z
1	6	0	1.555521	2.398046	0.234648
2	6	0	2.023469	0.972486	0.496697
3	8	0	1.204287	0.386749	1.563696
4	6	0	2.016144	0.073914	-0.742348

5	8	0	2.761690	-1.132086	-0.542647
6	6	0	4.155430	-1.002722	-0.823816
7	6	0	0.611045	-0.339761	-1.084860
8	6	0	-0.337438	-0.427191	-0.141577
9	6	0	-1.779607	-0.794380	-0.435605
10	6	0	-2.729799	0.416246	-0.236850
11	8	0	-2.245361	1.558657	-0.953939
12	6	0	-4.165278	0.083967	-0.626998
13	8	0	-2.211948	-1.916451	0.336564
14	6	0	0.022087	-0.175662	1.276314
15	8	0	-0.705002	-0.503094	2.212687
16	1	0	1.556680	2.966856	1.168543
17	1	0	0.546490	2.420140	-0.187774
18	1	0	2.238422	2.888058	-0.466286
19	1	0	3.025232	0.982438	0.931371
20	1	0	2.446612	0.642999	-1.577062
21	1	0	4.640730	-0.256341	-0.181663
22	1	0	4.605974	-1.979156	-0.629443
23	1	0	4.325420	-0.730376	-1.874380
24	1	0	0.390277	-0.597379	-2.117633
25	1	0	-1.842660	-1.102800	-1.486077
26	1	0	-2.692862	0.707699	0.818937
27	1	0	-2.307144	1.358939	-1.901246
28	1	0	-4.812140	0.949464	-0.453271
29	1	0	-4.224250	-0.184266	-1.689334
30	1	0	-4.546085	-0.757212	-0.041494
31	1	0	-2.009473	-1.688912	1.262293

5*S*,6*R*,8*R*,9*S*-**21f**			Standard Orientation (Ångstroms)		
Center number	Atomic number	Atomic type	X	Y	Z
1	6	0	1.649213	2.468107	0.470134
2	6	0	2.141687	1.026666	0.427787
3	8	0	1.419276	0.257462	1.448143
4	6	0	2.025592	0.387752	-0.960369
5	8	0	2.993623	-0.633840	-1.230819
6	6	0	2.966165	-1.790219	-0.390209
7	6	0	0.618645	-0.080346	-1.220987
8	6	0	-0.248661	-0.333667	-0.229329
9	6	0	-1.696747	-0.718679	-0.467437
10	6	0	-2.663819	0.428355	-0.070005
11	8	0	-2.258621	1.660199	-0.679578
12	6	0	-4.112520	0.092734	-0.404502
13	8	0	-2.044684	-1.935089	0.196293
14	6	0	0.211113	-0.259772	1.179282
15	8	0	-0.444151	-0.711685	2.116569
16	1	0	1.706142	2.849216	1.493373
17	1	0	0.614260	2.547509	0.122742
18	1	0	2.281220	3.094431	-0.166658
19	1	0	3.181571	0.972484	0.757711
20	1	0	2.271069	1.167373	-1.688714
21	1	0	3.712954	-2.475491	-0.798827
22	1	0	3.235933	-1.552729	0.645821
23	1	0	1.989475	-2.288614	-0.401279
24	1	0	0.320543	-0.218761	-2.257798
25	1	0	-1.822557	-0.908590	-1.540170
26	1	0	-2.563920	0.598858	1.007974
27	1	0	-2.373962	1.566869	-1.638374
28	1	0	-4.431273	-0.821149	0.103978
29	1	0	-4.769352	0.910884	-0.093151

30	1	0	-4.234557	-0.056578	-1.484690
31	1	0	-1.785104	-1.807672	1.126999

Table S24. Experimental ^{13}C NMR data of **21** and calculated ^{13}C NMR data of 5*S*,6*R*,8*S*,9*S*-**21**, 5*S*,6*R*,8*R*,9*R*-**21**, 5*S*,6*R*,8*S*,9*R*-**21**, and 5*S*,6*R*,8*R*,9*S*-**21** and the results analyzed by the improved probability DP4^{+} method based on their ^{13}C NMR data (ppm)

No.	Exp. ^{13}C NMR data	Cal. ^{13}C NMR data			
	**21**	5*S*,6*R*,8*S*,9*S*-**21**	5*S*,6*R*,8*R*,9*R*-**21**	5*S*,6*R*,8*S*,9*R*-**21**	5*S*,6*R*,8*R*,9*S*-**21**
2	163.2	175.393	176.126	176.718	176.59
3	128.2	141.343	141.012	141.131	141.621
4	146.2	154.793	154.671	157.959	154.975
5	82.1	79.3558	79.1205	81.2252	80.4549
6	68.0	89.0485	88.0939	87.1695	87.4071
7	18.5	20.6708	20.6317	19.9885	20.0945
8	77.9	87.7132	89.0215	86.2951	87.5914
9	66.7	77.2489	76.0955	77.0825	77.6157
10	17.6	20.6792	20.3655	22.9713	23.6744
11	57.0	59.9828	59.8379	60.2478	60.252
DP4^{+}		0.22%	0.36%	98.97%	0.46%

Table S25. Gibbs free energies[a] and equilibrium populations[b] of low-energy conformers of **26a**

Conformers	In MeOH	
	G^{a}	P (%) [b]
**26aa**	-1531.1217671	69.92
**26ab**	-1531.1195394	6.59
**26ac**	-1531.1196724	7.59
**26ad**	-1531.1203699	15.90

[a] B3LYP/6-31+G (d, p), in kcal/mol; [b] from ΔG values at 298.15K.

Table S26. Cartesian coordinates for the low-energy reoptimized MMFF conformers of **26a** at B3LYP/6-31+G(d,p) level of theory in MeOH

**26aa**			Standard Orientation (Ångstroms)		
Center number	Atomic number	Atomic type	X	Y	Z
1	8	0	2.062644	2.700095	-0.848964
2	6	0	1.862831	1.701071	0.158750
3	6	0	3.036778	0.736206	0.138084
4	6	0	4.350704	1.283502	0.111181
5	8	0	5.406560	0.396647	0.019194
6	6	0	5.250351	-0.960977	-0.071569
7	6	0	6.582807	-1.624526	-0.172028
8	6	0	4.013674	-1.528481	-0.056580
9	6	0	3.748765	-3.008118	-0.145780
10	8	0	4.648561	2.483130	0.152953
11	6	0	2.890899	-0.630252	0.069671
12	8	0	1.691210	-1.227921	0.120524
13	6	0	0.553844	0.951377	-0.152091
14	6	0	0.543754	-0.422080	0.561265
15	6	0	0.643157	-0.363499	2.090736
16	6	0	-0.664057	-1.251453	0.079935
17	8	0	-0.498653	-1.473598	-1.325733
18	1	0	0.588769	0.750248	-1.229397
19	6	0	-0.755279	1.731685	0.163620
20	6	0	-0.798906	3.135101	-0.461658
21	6	0	-2.013876	0.911156	-0.265681

22	6	0	-2.008836	-0.527093	0.328175
23	8	0	-2.327831	-0.413285	1.714433
24	6	0	-3.187440	-1.292968	-0.312391
25	6	0	-4.579023	-0.675855	-0.141160
26	6	0	-5.226845	-0.529126	-1.545468
27	6	0	-5.434013	-1.614842	0.734043
28	8	0	-3.044301	-2.375521	-0.862853
29	8	0	-2.085928	0.777266	-1.695414
30	6	0	-3.311479	1.620216	0.171898
31	6	0	-4.497922	0.721895	0.482018
32	8	0	-5.413662	1.146048	1.173859
33	1	0	2.930542	3.095954	-0.646974
34	1	0	1.798819	2.184581	1.145126
35	1	0	7.116671	-1.254910	-1.054921
36	1	0	7.189589	-1.374424	0.705668
37	1	0	6.498957	-2.707816	-0.242082
38	1	0	4.668163	-3.581442	-0.262304
39	1	0	3.235403	-3.365577	0.753614
40	1	0	3.097835	-3.233658	-0.997397
41	1	0	0.594042	-1.377549	2.497867
42	1	0	1.595915	0.076930	2.394655
43	1	0	-0.161398	0.221634	2.532644
44	1	0	-0.667915	-2.212152	0.610170
45	1	0	-1.161248	-2.145954	-1.563853
46	1	0	-0.822913	1.853234	1.250893
47	1	0	0.020091	3.752112	-0.091708
48	1	0	-1.733973	3.642674	-0.209537
49	1	0	-0.720877	3.089755	-1.551637
50	1	0	-2.260622	-1.288004	2.131171
51	1	0	-5.293473	-1.513200	-2.016894
52	1	0	-4.642012	0.127991	-2.193338
53	1	0	-6.235087	-0.118877	-1.440461
54	1	0	-5.483668	-2.605047	0.274576
55	1	0	-6.446924	-1.217002	0.825377
56	1	0	-5.010303	-1.714289	1.737717
57	1	0	-1.431837	0.101837	-1.961307
58	1	0	-3.150591	2.234024	1.060832
59	1	0	-3.620075	2.298393	-0.632315

**26ab**			Standard Orientation (Ångstroms)		
Center number	Atomic number	Atomic type	X	Y	Z
1	8	0	2.060003	2.712962	-0.818602
2	6	0	1.859625	1.704987	0.179560
3	6	0	3.031757	0.736627	0.143500
4	6	0	4.347947	1.282179	0.127057
5	8	0	5.400953	0.394022	0.019856
6	6	0	5.242969	-0.961433	-0.091880
7	6	0	6.574208	-1.625676	-0.201433
8	6	0	4.004781	-1.527164	-0.086226
9	6	0	3.738671	-3.005432	-0.193495
10	8	0	4.647315	2.479646	0.189375
11	6	0	2.886084	-0.627284	0.051641
12	8	0	1.678749	-1.220981	0.097400
13	6	0	0.549696	0.959360	-0.133986
14	6	0	0.541062	-0.413725	0.575594
15	6	0	0.664946	-0.373419	2.101413
16	6	0	-0.666105	-1.249147	0.087599
17	8	0	-0.568952	-1.473055	-1.321239

18	1	0	0.586730	0.756459	-1.210953
19	6	0	-0.765507	1.731388	0.170186
20	6	0	-0.807068	3.135932	-0.452368
21	6	0	-2.016031	0.902343	-0.276977
22	6	0	-2.008494	-0.529269	0.330019
23	8	0	-2.296905	-0.390165	1.723403
24	6	0	-3.203065	-1.338555	-0.228683
25	6	0	-4.587737	-0.672641	-0.169501
26	6	0	-5.123507	-0.539625	-1.623814
27	6	0	-5.535659	-1.567336	0.651764
28	8	0	-3.089047	-2.497693	-0.586224
29	8	0	-2.068720	0.767885	-1.705678
30	6	0	-3.315818	1.617577	0.141638
31	6	0	-4.514158	0.731592	0.432333
32	8	0	-5.439424	1.171428	1.102589
33	1	0	2.925099	3.111151	-0.610339
34	1	0	1.801950	2.177663	1.171421
35	1	0	6.488754	-2.707895	-0.284535
36	1	0	7.107226	-1.246464	-1.080765
37	1	0	7.182341	-1.386378	0.678273
38	1	0	4.660658	-3.581692	-0.266292
39	1	0	3.181490	-3.362411	0.679204
40	1	0	3.130249	-3.228835	-1.076942
41	1	0	-0.117463	0.232085	2.556111
42	1	0	0.590788	-1.388659	2.501938
43	1	0	1.633117	0.037181	2.398394
44	1	0	-0.669710	-2.210323	0.618553
45	1	0	0.342166	-1.749839	-1.511110
46	1	0	-0.846984	1.848872	1.256989
47	1	0	-1.745027	3.641851	-0.208420
48	1	0	-0.718435	3.092791	-1.541729
49	1	0	0.007345	3.753833	-0.073817
50	1	0	-2.228407	-1.258310	2.154470
51	1	0	-4.476925	0.089346	-2.238890
52	1	0	-6.127819	-0.106730	-1.603390
53	1	0	-5.177795	-1.533438	-2.077063
54	1	0	-6.543067	-1.145964	0.652773
55	1	0	-5.196939	-1.652640	1.688841
56	1	0	-5.572233	-2.567106	0.213452
57	1	0	-1.429268	0.076212	-1.963806
58	1	0	-3.164962	2.230020	1.033377
59	1	0	-3.606228	2.299156	-0.666436

**26ac**			Standard Orientation (Ångstroms)		
Center number	Atomic number	Atomic type	X	Y	Z
1	8	0	2.024402	2.738758	-0.723031
2	6	0	1.865557	1.703611	0.254898
3	6	0	3.033820	0.736932	0.136749
4	6	0	4.348430	1.283589	0.080824
5	8	0	5.393961	0.401109	-0.112307
6	6	0	5.229271	-0.948859	-0.271221
7	6	0	6.553038	-1.606050	-0.474114
8	6	0	3.992435	-1.515780	-0.225573
9	6	0	3.719328	-2.989157	-0.374682
10	8	0	4.651888	2.477781	0.176601
11	6	0	2.882873	-0.622657	0.001050
12	8	0	1.678416	-1.217958	0.083662
13	6	0	0.541175	0.968116	-0.022104
14	6	0	0.565882	-0.426945	0.641887

15	6	0	0.766077	-0.432179	2.160489
16	6	0	-0.662690	-1.251396	0.192050
17	8	0	-0.626776	-1.464575	-1.221576
18	1	0	0.529658	0.801839	-1.105996
19	6	0	-0.762890	1.727869	0.364562
20	6	0	-0.809868	3.164061	-0.179975
21	6	0	-2.015774	0.906435	-0.088671
22	6	0	-1.994944	-0.531717	0.500809
23	8	0	-2.203960	-0.424641	1.910615
24	6	0	-3.197798	-1.357371	-0.025755
25	6	0	-4.576801	-0.694487	-0.169035
26	6	0	-5.296024	-1.278418	-1.399303
27	6	0	-5.384840	-1.031736	1.121638
28	8	0	-3.080958	-2.559514	-0.199655
29	8	0	-2.087660	0.790991	-1.520787
30	6	0	-3.336009	1.580063	0.306249
31	6	0	-4.524322	0.835250	-0.261777
32	8	0	-5.467388	1.446701	-0.745644
33	1	0	2.895957	3.134318	-0.538794
34	1	0	1.853246	2.148179	1.261120
35	1	0	7.202751	-1.406661	0.385620
36	1	0	6.462785	-2.683352	-0.602820
37	1	0	7.043934	-1.186133	-1.359405
38	1	0	4.635430	-3.561927	-0.516754
39	1	0	3.207616	-3.378997	0.511730
40	1	0	3.065647	-3.179651	-1.233129
41	1	0	1.749228	-0.032168	2.420289
42	1	0	0.008945	0.160866	2.670683
43	1	0	0.709361	-1.458533	2.534342
44	1	0	-0.649524	-2.217654	0.712048
45	1	0	0.274811	-1.741646	-1.452820
46	1	0	-0.810488	1.789382	1.457906
47	1	0	-1.689813	3.695110	0.191175
48	1	0	-0.837891	3.176671	-1.273928
49	1	0	0.067110	3.727237	0.137748
50	1	0	-2.128396	-1.307224	2.309404
51	1	0	-6.296870	-0.850727	-1.481380
52	1	0	-5.381145	-2.362714	-1.305684
53	1	0	-4.745066	-1.049916	-2.317055
54	1	0	-4.916765	-0.616028	2.017250
55	1	0	-5.448718	-2.117756	1.233833
56	1	0	-6.395754	-0.624887	1.029548
57	1	0	-1.457804	0.097465	-1.798676
58	1	0	-3.429370	1.622817	1.396895
59	1	0	-3.389831	2.597838	-0.080134

**26ad**			Standard Orientation (Ångstroms)		
Center number	Atomic number	Atomic type	X	Y	Z
1	8	0	2.035565	2.725409	-0.768851
2	6	0	1.868253	1.701307	0.219874
3	6	0	3.041531	0.739142	0.131797
4	6	0	4.353394	1.289607	0.082047
5	8	0	5.406046	0.408744	-0.079634
6	6	0	5.247584	-0.944709	-0.217853
7	6	0	6.576186	-1.601242	-0.389840
8	6	0	4.012681	-1.514970	-0.180239
9	6	0	3.745346	-2.990835	-0.313799
10	8	0	4.651609	2.487339	0.159150
11	6	0	2.894725	-0.624183	0.019341

12	8	0	1.697937	-1.224793	0.092996
13	6	0	0.548475	0.958997	-0.065072
14	6	0	0.569256	-0.434930	0.606136
15	6	0	0.738655	-0.419532	2.131085
16	6	0	-0.656084	-1.254212	0.152099
17	8	0	-0.539373	-1.451769	-1.262751
18	1	0	0.544258	0.791343	-1.148729
19	6	0	-0.753623	1.724949	0.319702
20	6	0	-0.801271	3.155268	-0.240931
21	6	0	-2.015093	0.907411	-0.111835
22	6	0	-1.993412	-0.535022	0.470306
23	8	0	-2.220905	-0.445355	1.875375
24	6	0	-3.183476	-1.325809	-0.129978
25	6	0	-4.583994	-0.704118	-0.103088
26	6	0	-5.414988	-1.258578	-1.274081
27	6	0	-5.267327	-1.092270	1.245385
28	8	0	-3.019472	-2.461482	-0.555312
29	8	0	-2.109058	0.788575	-1.543118
30	6	0	-3.323807	1.585976	0.310314
31	6	0	-4.552045	0.832388	-0.156862
32	8	0	-5.543964	1.442764	-0.529869
33	1	0	2.907074	3.120508	-0.582198
34	1	0	1.838957	2.159513	1.219776
35	1	0	7.211734	-1.386398	0.476789
36	1	0	6.490352	-2.680890	-0.501494
37	1	0	7.080281	-1.194745	-1.273969
38	1	0	3.057012	-3.185252	-1.143318
39	1	0	4.659118	-3.556972	-0.493111
40	1	0	3.273015	-3.383555	0.593413
41	1	0	0.690986	-1.442808	2.514348
42	1	0	1.712405	-0.003541	2.400932
43	1	0	-0.033447	0.167384	2.624711
44	1	0	-0.641744	-2.224669	0.662188
45	1	0	-1.174008	-2.153326	-1.486816
46	1	0	-0.792332	1.799948	1.412590
47	1	0	0.074744	3.722787	0.071549
48	1	0	-1.682453	3.689338	0.122991
49	1	0	-0.828156	3.155515	-1.334900
50	1	0	-2.135890	-1.329537	2.267590
51	1	0	-5.497131	-2.344448	-1.198225
52	1	0	-4.951558	-1.011901	-2.234340
53	1	0	-6.417057	-0.827116	-1.251957
54	1	0	-5.285362	-2.181777	1.342776
55	1	0	-6.296631	-0.722886	1.237405
56	1	0	-4.746082	-0.672147	2.107147
57	1	0	-1.466451	0.108716	-1.827280
58	1	0	-3.367353	1.674486	1.401622
59	1	0	-3.397160	2.589172	-0.110440

Table S27. Gibbs free energies[a] and equilibrium populations[b] of low-energy conformers of **26b**

Conformers	In MeOH	
	G [a]	*P* (%) [b]
**26ba**	-1531.1126295	55.45
**26bb**	-1531.1124232	44.55

[a] B3LYP/6-31+G(d,p), in kcal/mol; [b] from Δ*G* values at 298.15K.

Table S28. Cartesian coordinates for the low-energy reoptimized MMFF conformers of **26b** at B3LYP/6-31+G(d,p) level of theory in MeOH

**26ba**			Standard Orientation (Ångstroms)		
Center number	Atomic number	Atomic type	X	Y	Z
1	8	0	-1.830150	2.771972	-0.652181
2	6	0	-1.799547	1.702757	0.300660
3	6	0	-3.049415	0.853184	0.125035
4	6	0	-4.304168	1.521575	0.033621
5	8	0	-5.419451	0.745195	-0.215518
6	6	0	-5.376552	-0.611863	-0.392957
7	6	0	-6.747239	-1.136243	-0.661741
8	6	0	-4.201666	-1.294262	-0.307916
9	6	0	-4.061681	-2.784793	-0.469794
10	8	0	-4.498628	2.737318	0.143944
11	6	0	-3.022248	-0.512983	-0.027487
12	8	0	-1.883635	-1.221253	0.088223
13	6	0	-0.545743	0.848792	0.037620
14	6	0	-0.718936	-0.544502	0.687454
15	6	0	-0.969957	-0.537448	2.198662
16	6	0	0.438490	-1.488584	0.270984
17	8	0	0.405710	-1.727551	-1.136706
18	1	0	-0.533020	0.691830	-1.047520
19	6	0	0.821024	1.474032	0.449423
20	6	0	1.020173	2.912246	-0.050413
21	6	0	1.977180	0.539854	-0.031614
22	6	0	1.820882	-0.880118	0.584758
23	8	0	1.930299	-0.890055	2.006984
24	6	0	2.937239	-1.801657	0.065093
25	6	0	5.734474	0.428219	-0.395859
26	6	0	6.759034	-0.603468	-0.793872
27	6	0	6.250943	1.843478	-0.367305
28	8	0	2.810700	-3.002409	-0.080079
29	8	0	1.999793	0.421104	-1.460917
30	6	0	3.370062	1.062060	0.342710
31	6	0	4.468154	0.116566	-0.075587
32	8	0	4.155782	-1.262999	-0.120913
33	1	0	-2.668490	3.240614	-0.485135
34	1	0	-1.773487	2.119205	1.318568
35	1	0	-7.174550	-0.622485	-1.530158
36	1	0	-7.400454	-0.929873	0.193967
37	1	0	-6.750342	-2.207712	-0.854530
38	1	0	-3.583657	-3.224200	0.411891
39	1	0	-5.025036	-3.273958	-0.610537
40	1	0	-3.430146	-3.026964	-1.332100
41	1	0	-1.007466	-1.565506	2.569618
42	1	0	-1.925839	-0.059401	2.425733
43	1	0	-0.185249	-0.009588	2.737386
44	1	0	0.338470	-2.434482	0.815592
45	1	0	-0.512473	-1.934510	-1.375716
46	1	0	0.870626	1.497414	1.543954
47	1	0	1.052612	2.956263	-1.143450
48	1	0	1.952429	3.331132	0.337847
49	1	0	0.208411	3.556920	0.285941
50	1	0	2.734119	-0.424830	2.285480
51	1	0	7.597190	-0.601218	-0.084769
52	1	0	7.182204	-0.353573	-1.775340
53	1	0	6.354259	-1.614440	-0.842290

54	1	0	7.100477	1.917651	0.324224
55	1	0	6.630241	2.130803	-1.356637
56	1	0	5.510888	2.583653	-0.060021
57	1	0	1.330660	-0.241021	-1.721963
58	1	0	3.414511	1.271830	1.419451
59	1	0	3.526709	2.014802	-0.161024

**26bb**			Standard Orientation (Ångstroms)		
Center number	Atomic number	Atomic type	X	Y	Z
1	8	0	-1.818237	2.766074	-0.654108
2	6	0	-1.797784	1.690475	0.293657
3	6	0	-3.052868	0.852198	0.116365
4	6	0	-4.301839	1.528875	0.033045
5	8	0	-5.426106	0.761099	-0.204912
6	6	0	-5.392541	-0.596632	-0.383548
7	6	0	-6.769494	-1.114457	-0.632407
8	6	0	-4.221093	-1.285454	-0.315649
9	6	0	-4.089601	-2.775598	-0.486289
10	8	0	-4.487248	2.747162	0.142519
11	6	0	-3.031711	-0.514954	-0.041091
12	8	0	-1.903176	-1.231060	0.055574
13	6	0	-0.547351	0.832800	0.027979
14	6	0	-0.725240	-0.575905	0.641807
15	6	0	-0.959209	-0.596757	2.158267
16	6	0	0.437180	-1.509577	0.200743
17	8	0	0.369741	-1.813807	-1.183818
18	1	0	-0.514355	0.702278	-1.060181
19	6	0	0.809496	1.451511	0.482579
20	6	0	1.006401	2.909927	0.037949
21	6	0	1.979428	0.534159	0.010310
22	6	0	1.814529	-0.901105	0.571830
23	8	0	2.004363	-0.813839	1.983430
24	6	0	2.951241	-1.805494	0.069141
25	6	0	5.720748	0.452439	-0.397051
26	6	0	6.741272	-0.564235	-0.842345
27	6	0	6.232220	1.868985	-0.348786
28	8	0	2.842129	-3.011814	-0.049496
29	8	0	1.953503	0.342485	-1.418698
30	6	0	3.368524	1.043871	0.418204
31	6	0	4.464828	0.124074	-0.052406
32	8	0	4.162340	-1.256006	-0.121797
33	1	0	-2.656781	3.235442	-0.489320
34	1	0	-1.768569	2.102838	1.313400
35	1	0	-7.414505	-0.882918	0.223003
36	1	0	-6.782098	-2.190314	-0.798661
37	1	0	-7.199653	-0.618166	-1.509679
38	1	0	-5.046288	-3.250143	-0.702997
39	1	0	-3.399647	-3.011935	-1.303577
40	1	0	-3.679805	-3.233985	0.420429
41	1	0	-1.896559	-0.094157	2.409977
42	1	0	-0.152704	-0.111739	2.705138
43	1	0	-1.030180	-1.633321	2.500963
44	1	0	0.327112	-2.462169	0.729213
45	1	0	0.763615	-1.062263	-1.665469
46	1	0	0.840159	1.448115	1.577219
47	1	0	1.938212	3.316687	0.438790
48	1	0	0.192901	3.534484	0.407057
49	1	0	1.023789	3.017367	-1.052319
50	1	0	1.840264	-1.686943	2.375974

51	1	0	7.134232	-0.295123	-1.831324
52	1	0	6.343200	-1.577592	-0.895765
53	1	0	7.599434	-0.566703	-0.157605
54	1	0	5.496533	2.599806	-0.010306
55	1	0	7.095610	1.931912	0.326499
56	1	0	6.590353	2.178774	-1.339298
57	1	0	2.025731	1.201847	-1.861470
58	1	0	3.398783	1.162740	1.507180
59	1	0	3.529919	2.027963	-0.020253

Table S29. Experimental ^{13}C NMR data of **26** and calculated ^{13}C NMR data of **26a** and **26b** and the results analyzed by the improved probability DP4^{+} method based on their ^{13}C NMR data (ppm)

No.	Exp. ^{13}C NMR data	Cal. ^{13}C NMR data	
	**26**	**26a**	**26b**
1	162.3	173.1250	173.1350
3	157.1	172.3540	172.3130
4	106.5	117.7660	117.7740
4a	162.1	173.4680	173.4960
5a	83.7	93.6201	93.4977
6	72.5	83.4806	83.3901
6a	75.2	85.1116	85.4291
7	207.5	231.0700	231.3080
8	54.4	64.7272	64.4261
9	209.4	228.9320	228.8030
10	45.2	53.7828	54.5642
10a	75.2	84.4812	84.1959
11	38.7	47.5264	48.0497
11a	39.6	46.1643	46.1687
12	62.5	71.1033	71.1162
12a	102.2	108.5440	108.4260
13	17	19.3637	19.3580
14	9.1	13.4636	13.4669
15	17.5	20.8077	20.8413
16	23.3	28.2063	26.8544
17	25.2	28.4972	29.6520
18	10.8	14.3049	14.2815
DP4^{+}		77.86%	22.14%
